# Structural basis of lenacapavir-induced HIV-1 capsid defects during virion maturation

**DOI:** 10.1038/s41467-026-77803-7

**Published:** 2026-09-22

**Authors:** Hiroki Tanaka, Reina Morita, Tomomasa Oka, Minoru Fukushima, Shunsuke Kita, Mina Sasaki, Katsumi Maenaka, Shinichi Machida

**Affiliations:** 1 Department of Structural Virology, National Institute of Global Health and Medicine, Japan Institute for Health Security, Tokyo, Japan; 2 Laboratory of Biomolecular Science, Faculty of Pharmaceutical Sciences, Hokkaido University, Sapporo, Japan; 3 Center for Research and Education on Drug Discovery, Faculty of Pharmaceutical Sciences, Hokkaido University, Sapporo, Japan; 4 Global Station for Biosurfaces and Drug Discovery, Hokkaido University, Sapporo, Japan; 5 Hokkaido University Institute for Vaccine Research & Development, Sapporo, Japan; 6 Graduate School of Medical Sciences, Kumamoto University, Kumamoto, Japan

**Keywords:** Retrovirus, Cryoelectron microscopy, Cryoelectron tomography

## Abstract

Long-acting lenacapavir (LEN) has emerged as a highly effective, potentially game-changing therapy for HIV treatment and prevention. Its mechanism of action in the early phase of HIV-1 replication, when the capsid directs key post-entry steps such as reverse transcription, nuclear import, and integration, has been well characterized. In contrast, its effects during the late phase of replication, when the capsid assembles and matures within budding virions, remain poorly understood. Here, we determine the cryo-electron microscopy structure of the mature HIV-1 capsid lattice assembled within virus-like particles in the presence of LEN. Our structural analyses reveal that LEN alters interhexamer interactions, perturbs the capsid lattice curvature, and thereby prevents the formation of a functional cone-shaped capsid. Biochemical analyses further demonstrate that LEN-containing cores lose reverse transcriptase because of compromised capsid integrity, whereas integrase and viral RNA remain associated. Functionally, viruses produced in the presence of LEN exhibit markedly reduced infectivity, low reverse transcription activity, and poor integration. Taken together, these findings provide mechanistic insights into the late-phase action of LEN and provide key directions for the design of future inhibitors.

## Abstract (web summary)

Long-acting lenacapavir is a HIV-1 capsid inhibitor. Here the authors reveal that lenacapavir alters capsid lattice curvature during virion maturation, preventing formation of a functional cone-shaped capsid.

## Introduction

The HIV-1 capsid is a multifunctional structural protein complex that not only encloses the viral RNA genome but also regulates reverse transcription, nuclear import, and integration site selection, as well as evasion of immune sensing by cytosolic DNA sensors^1–5^. The HIV-1 capsid is composed of capsid protein (CA), which is released following the cleavage of the Gag polyprotein during viral maturation^6–8^. CA contains two distinct domains, the N-terminal domain (NTD) and the C-terminal domain (CTD), connected by a flexible linker. In mature virions, CA monomers assemble into a fullerene-like cone in which approximately 200 CA hexamers and 12 CA pentamers are arranged in a continuous lattice to form the conical capsid^9–13^. The CA-NTD stabilizes each hexamer through interactions around the sixfold axis, whereas the CA-CTD mediates dimeric and trimeric contacts, involving helix 9 and primarily helix 10 of the CTD, respectively, to interconnect hexamers and build the lattice^13–15^. Inositol hexakisphosphate (IP6) further stabilizes the lattice by binding to a ring of arginine residues within the CA-NTD, thereby promoting proper capsid assembly^16,17^. The precise balance between the stability and instability of the capsid is critical for its function during the viral life cycle^18–22^. Accordingly, the capsid represents a pivotal target for HIV-1 therapeutic intervention.

Lenacapavir (LEN, also known as GS-6207) is a HIV capsid inhibitor that is highly potent and has long-acting activity, allowing it to be administered as infrequently as twice per year^23,24^. Furthermore, preexposure prophylaxis (PrEP) with LEN administered twice a year has been shown to markedly reduce the risk of HIV acquisition^25^. LEN has been shown to inhibit multiple steps of the viral replication cycle. Previous studies have demonstrated that LEN and GS-CA1, an archetypal predecessor of LEN, target the mature HIV-1 capsid and bind to the FG (phenylalanine–glycine motif)-binding pocket located at the interface between the NTD and the CTD of CA monomers, thereby altering the stability of the mature capsid, potentially modulating host factor binding to CA and inhibiting reverse transcription, nuclear import, and integration^26–30^. Although the inhibitory effects of LEN have been characterized primarily in the context of post-entry processes, its mechanistic impact during the late phase of HIV-1 replication, when the capsid assembles and matures within budding virions, remains poorly understood. Notably, LEN also binds to Gag monomers with high affinity, leading to the formation of an aberrant capsid in virions and a marked reduction in viral infectivity^26,27^. In addition, a previous study suggested that LEN preferentially stabilizes the hexameric dimer interface over the pentameric interface, thereby biasing assembly towards hexamer formation^31^. Furthermore, a previous structural study revealed that LEN binds to the immature HIV-1 Gag lattice^32^.

Despite these studies, how LEN-bound capsids reorganize during virion assembly and whether LEN incorporation alters the curvature parameters required for proper cone closure remain unclear. Although LEN has been shown to stabilize the CA hexamer lattice, LEN-treated capsids fail to form properly closed conical cores. Thus, determining whether and how LEN reshapes the capsid curvature during virion maturation is essential to fully understand its antiviral mechanism.

In this study, we determine the cryo-electron microscopy structure of the capsid lattice assembled within virus-like particles in the presence of LEN. Our structural analyses reveal that LEN enforces a flattened lattice geometry by rearranging the intrahexamer NTD–CTD interface and the interhexamer CTD–CTD trimer interface. This lattice remodeling compromises core integrity and prevents productive reverse transcription and infection.

## Results

### LEN perturbs capsid lattice curvature in mature HIV-1 VLPs

**Fig. 1:**
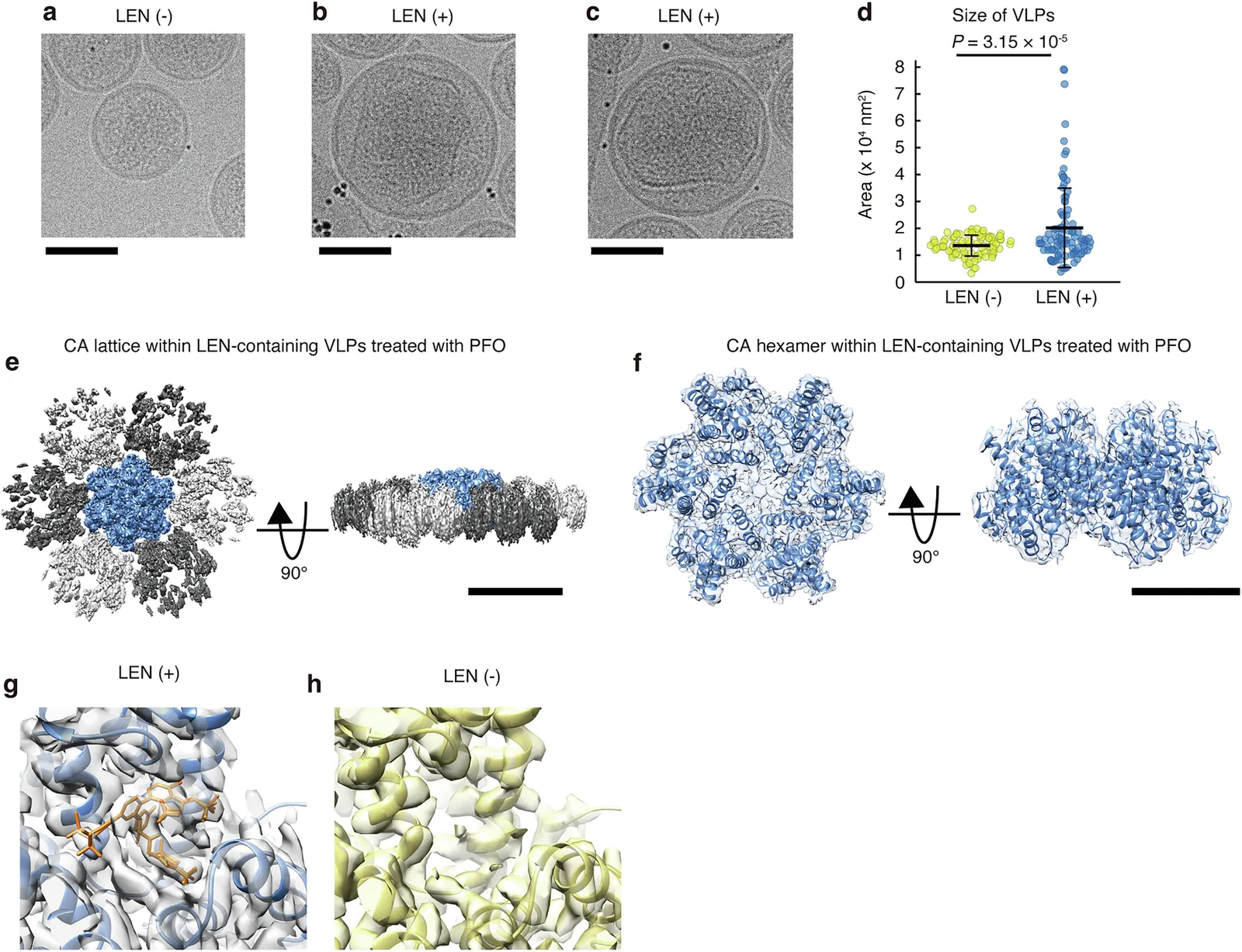
Cryo-EM structure of the HIV-1 capsid lattice within the VLPs produced in the presence of LEN. **a–c** Representative cryo-EM images of the VLPs produced in the absence (**a**) or presence of 50 nM LEN (**b**, **c**). Scale bar, 100 nm. More than three independent experiments were repeated, and the results were reproduced. **d** Size distribution of VLPs produced in the presence or absence of LEN. Scatter plot showing the area of the VLPs produced in the absence (yellow) or presence of 50 nM LEN (blue), as measured from cryo-EM micrographs. Each dot represents an individual VLP (*n* = 101 per group), and the horizontal bars indicate the means ± s.d. (*P* = 3.15 × 10⁻⁵, unpaired two-tailed Welch’s t test). **e** Cryo-EM density map of the CA lattice within the LEN-containing VLPs, shown from the top (left panel) and side (right panel) views. Scale bar, 100 Å. **f** Atomic model of a CA hexamer in the LEN-bound lattice fitted into the cryo-EM density map shown in (**e**). Scale bar, 50 Å. **g** Close-up view of the cryo-EM map of a LEN-bound CA lattice (**e**) and the atomic model (**f**) showing the density corresponding to a bound LEN. **h** Close-up view of the cryo-EM map of a LEN-unbound CA lattice (Supplementary Fig. 3 and 4) and its atomic model, showing the absence of a density corresponding to the bound LEN.

To investigate the mechanistic effects of LEN during the late phase of HIV-1 replication, we examined the in situ structure of the HIV-1 capsid lattice within Gag virus-like particles (VLPs) produced in the presence of LEN. The VLPs produced from 293T cells in the absence or presence of LEN were vitrified and imaged using cryo-electron microscopy (cryo-EM) (Fig. 1a–c). Consistent with the findings of previous studies^27^, the VLPs produced in the presence of LEN predominantly contained irregularly shaped capsids rather than typical cone-shaped capsids (Fig. 1a–c). Interestingly, VLPs produced in the presence of LEN also showed a broader size distribution, and larger particles appeared more frequently in the presence of LEN than in its absence (Fig. 1d and Supplementary Table 1). To obtain a higher-resolution view of the lattice architecture, we next treated the VLPs with perfringolysin O (PFO). PFO selectively perforated the viral membrane with a limited number of ~20–30 nm pores, thereby releasing excess free CA and increasing the signal-to-noise ratio in the cryo-EM images while maintaining overall membrane integrity and preserving the internal capsid structure in an apparently unaltered state^22,33,34^. For this structural analysis, we used VLPs produced in the presence of 50 nM LEN to ensure near-complete lattice occupancy. We then determined the capsid lattice structure within VLPs produced in the presence and absence of LEN using a single-particle cryo-EM approach (Fig. 1e and Supplementary Figs. 1–4, Supplementary Table 2). The reconstruction of the LEN-bound lattice clearly resolved the central CA hexamer (Fig. 1f), and neighboring hexamers were also discernible (Fig. 1e). A distinct density corresponding to LEN was observed at the expected FG binding pocket (Fig. 1g, h).

**Fig. 2:**
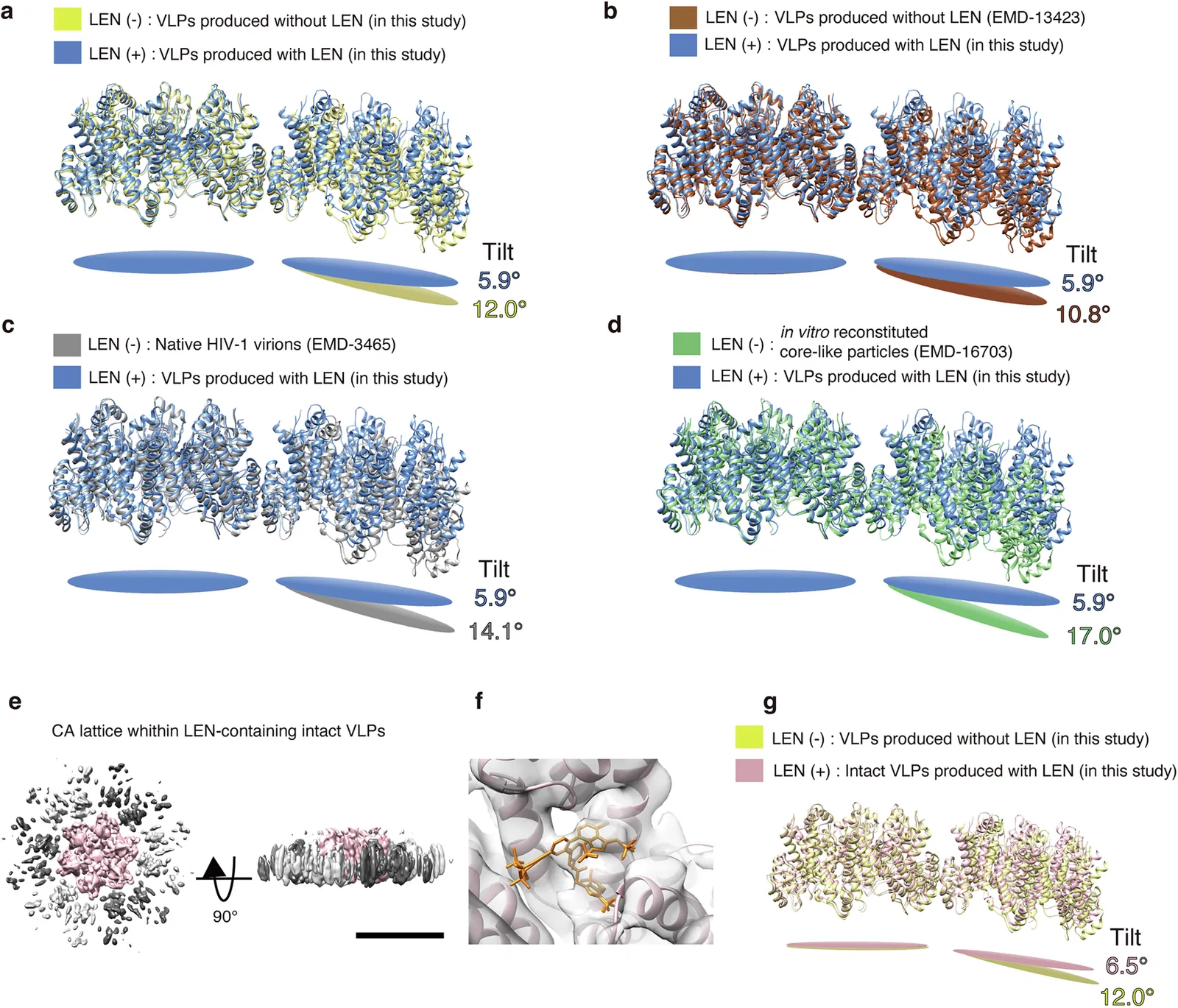
LEN reduces the interhexamer curvature in the HIV-1 capsid lattice. **a** Comparison of two adjacent CA hexamers from the LEN-bound (blue) and LEN-unbound (yellow) capsid lattices determined in this study. **b–d** Comparison of the LEN-bound CA lattice determined in this study with previously reported LEN-unbound lattices from VLPs produced without LEN (brown; EMD-13423) (**b**), HIV-1 virions produced without LEN (gray; EMD-3465) (**c**), and in vitro-reconstituted core-like particles (green; EMD-16703) (**d**). **e** Cryo-EM density map of the CA lattice within LEN-containing intact VLPs, shown from the top (left panel) and side (right panel) views. Scale bar, 50 Å. **f** Close-up view showing the density corresponding to the bound LEN in the CA lattice within intact LEN-containing VLPs and the fitted atomic model. **g** Comparison of two adjacent CA hexamers from the LEN-bound lattice within intact VLPs (pink) and the LEN-unbound lattice determined in this study (yellow).

Since proper cone formation requires a precise curvature between neighboring hexamers, we next quantified the effect of LEN on the lattice curvature. To this end, we first compared the hexameric CA lattice within VLPs produced in the absence and presence of LEN (Fig. 2a). The tilt angle between adjacent CA hexamers was 12.0° in the LEN-unbound structure, whereas the corresponding angle in LEN-containing VLPs was reduced to 5.9°, indicating that LEN binding induces a pronounced flattening of the lattice curvature (Fig. 2a). We further compared the hexameric CA lattice resolved in our LEN-containing VLPs with published structures of VLPs or virions produced without LEN, as well as to in vitro-reconstituted core-like particles (CLPs)^13,15,34^ (EMD-13423, 3465 and 16703), and calculated the interhexamer tilt angles (Fig. 2b–d). While the tilt angles between adjacent CA hexamers ranged from 10.8° to 17.0° in previously reported LEN-unbound structures, the corresponding angle in our LEN-containing VLPs was 5.9°. These comparisons support the conclusion that LEN reduces the interhexamer curvature and enforces a flattened lattice geometry. We also determined the capsid lattice structure of LEN-containing VLPs without PFO perforation to exclude the possibility that the observed flattening was caused by PFO treatment and confirmed that a similar flattened lattice conformation was present within intact VLPs (Fig. 2e–g and Supplementary Fig. 5–6, Supplementary Table 2). Taken together, these results suggest that LEN binding perturbs the local curvature necessary for the assembly of a functional conical capsid.

**Fig. 3:**
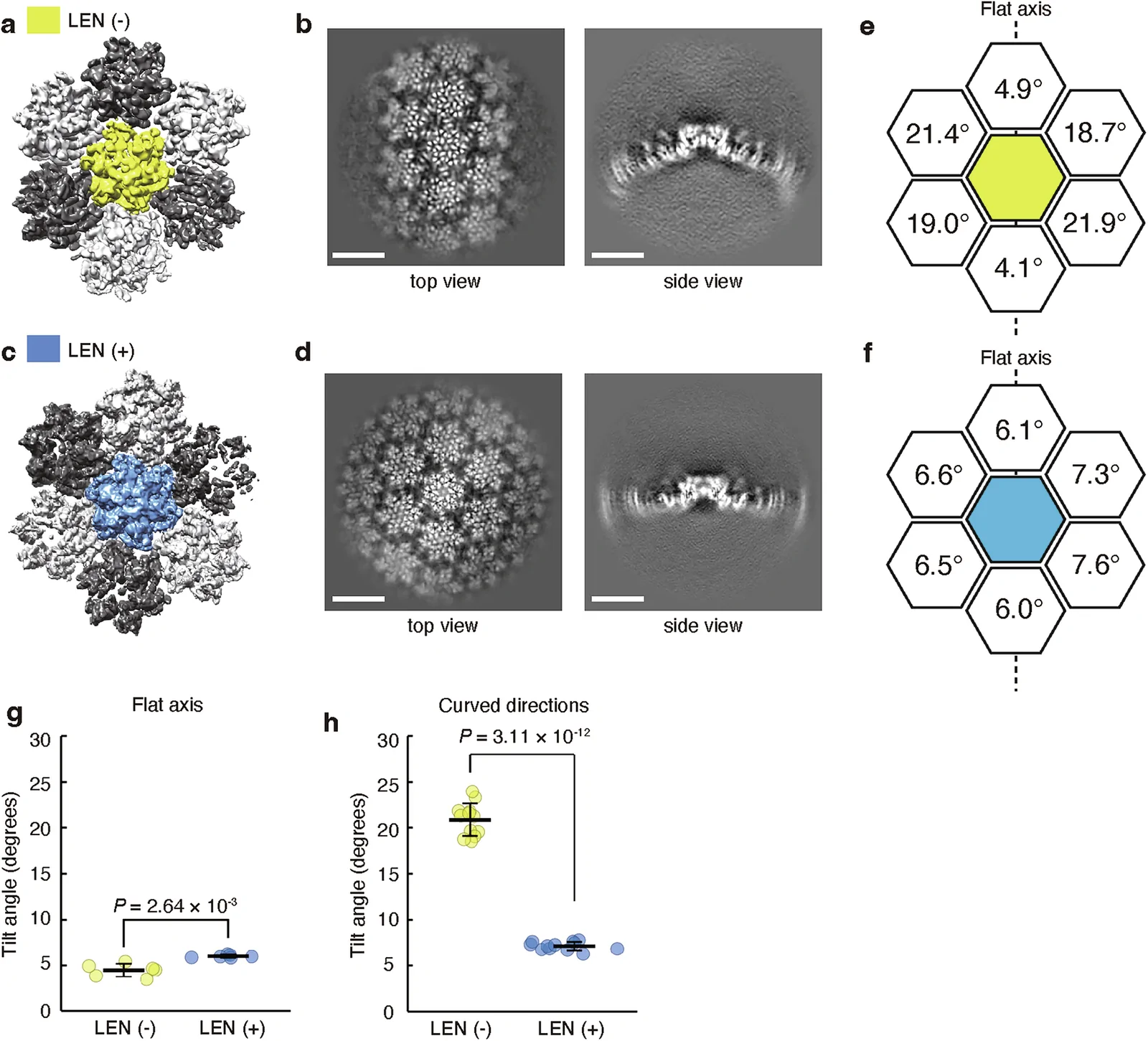
LEN-induced capsid lattice flattening is observed across heterogeneous capsid lattice conformations. **a–d** Cryo-EM density maps of CA lattices from VLPs produced in the absence (**a**) or presence (**c**) of LEN, refined without imposing symmetry. Orthoslices from the corresponding cryo-EM density maps are shown for VLPs produced in the absence (**b**) or presence (**d**) of LEN. Scale bar, 100 Å. **e**, **f** Schematic representations of interhexamer tilt angles around the central CA hexamer in the LEN-unbound (**a**) and LEN-bound (**c**) lattices. **g, h** Interhexamer tilt angle distributions after classification into three structural classes (Supplementary Figs. 2 and 4), comparing LEN-unbound and LEN-bound lattices along the relatively flat axis (**g**) and the more curved directions (**h**). Horizontal bars indicate the means ± s.d. from the three classified maps. *P* = 2.64 × 10⁻^3^ (**g**), and *P* = 3.11 × 10⁻^12^ (**h**); unpaired two-tailed Welch’s t test.

To further assess CA lattice heterogeneity and determine whether LEN-induced flattening was observed across different capsid lattice conformations, we analysed the CA lattice within VLPs produced in the absence or presence of LEN, by refining them without imposing symmetry (Fig. 3a–d and Supplementary Figs. 2 and 4). In the LEN-unbound lattice, the tilt angles around the central hexamer showed substantial directional variation, consistent with the curved geometry of the capsid lattice (Fig. 3a, b, e). In contrast, LEN-bound lattices showed reduced tilt angles in curved directions, whereas the tilt along the relatively flat axis remained largely unchanged (Fig. 3c, d, f). Because the consensus maps represent the averaged lattice conformations, we further classified the particles into three structural classes (Supplementary Figs. 2 and 4) and quantified the interhexamer tilt angles in each class along the relatively flat axis and the more curved directions (Fig. 3g, h). Along the relatively flat axis, the tilt angle distributions were largely similar between LEN-unbound and LEN-bound lattices (Fig. 3g). In contrast, in more curved directions, LEN-bound lattices consistently exhibited lower tilt angles than LEN-unbound lattices across the classified conformations (Fig. 3h). These results indicate that LEN-induced lattice flattening is not restricted to a single averaged reconstruction but represents a reproducible structural feature of LEN-bound capsid lattices.

### LEN remodels intra- and interhexamer capsid interfaces

**Fig. 4:**
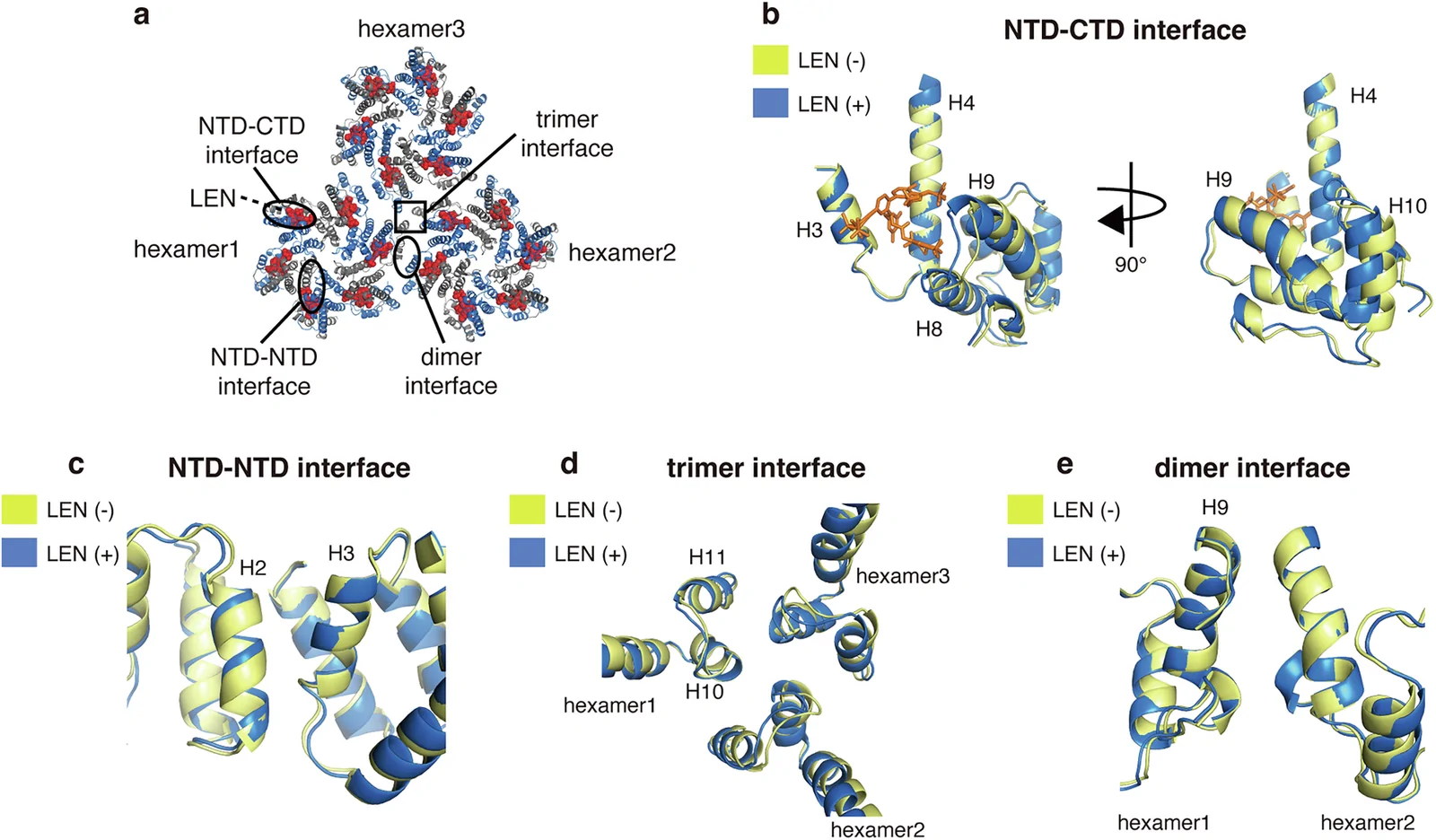
LEN rearranges the intrahexamer NTD–CTD interface and interhexamer trimer interface. **a** Overview of intra- and interhexamer interfaces within the HIV-1 capsid lattice. Bound LEN molecules are shown as red spheres. **b** Comparison of the NTD–CTD interface between CA molecules in the region surrounding the FG pocket based on the cryo-EM structures of the LEN-bound and LEN-unbound CA lattices. The LEN-bound CA lattice (blue) was superimposed onto the LEN-unbound CA lattice (yellow) to visualize structural differences at the NTD–CTD interface. Structures were superimposed by aligning one helix 4 of the CA-NTD. **c** Comparison of the NTD–NTD interface between CA molecules mediated by helices 2 and 3, based on the cryo-EM structures of the LEN-bound and LEN-unbound CA lattices. The LEN-bound CA lattice (blue) was superimposed onto the LEN-unbound CA lattice (yellow) to visualize structural differences at the NTD–NTD interface. Structures were superimposed by aligning one helix 3 of the CA-NTD. **d** Comparison of the CTD-mediated trimer interface between CA molecules mediated by primary helix 10, based on the cryo-EM structures of the LEN-bound and LEN-unbound CA lattices. The LEN-bound CA lattice (blue) was superimposed onto the LEN-unbound CA lattice (yellow) to visualize structural differences at the trimeric interface. Structures were superimposed by aligning one helix 10 of the CA-CTD of hexamer 1. **e** Comparison of the CTD–CTD dimer interface between CA molecules mediated by helix 9, based on the cryo-EM structures of the LEN-bound and LEN-unbound CA lattices. The LEN-bound CA lattice (blue) was superimposed onto the LEN-unbound CA lattice (yellow) to visualize structural differences at the dimeric interface. Structures were superimposed by aligning one helix 9 of the CA-CTD of hexamer 1.

Next, we examined how LEN binding alters the lattice geometry at the molecular level. In the CA lattice structure within LEN-containing VLPs, conformational changes were detected in the region surrounding the FG pocket, the binding site of LEN (Fig. 4a, b). Compared with the LEN-unbound lattice, the LEN-bound lattice showed closer positioning of NTD helix 4 and CTD helix 9 from neighboring CA subunits within the same hexamer, indicating a local rearrangement of the intrahexamer NTD–CTD interface (Fig. 4b and Supplementary Fig. 7a). In contrast, the NTD–NTD interface, which stabilizes each hexamer and coordinates IP6 binding at the central arginine ring, showed only minor changes (Fig. 4c and Supplementary Fig. 7b). Normally, interhexamer contacts in a curved lattice are accommodated by the outwards displacement of the CTDs relative to adjacent NTDs^13,14^; thus, we subsequently examined the local conformational changes at the CA interfaces that mediate hexamer–hexamer interactions within the lattice (Fig. 4d, e). LEN binding induced structural rearrangements at the CTD-mediated trimer interface formed primarily by helix 10 (Fig. 4d and Supplementary Fig. 7c), whereas the rearrangement of the CTD–CTD dimer interface formed by helix 9 was relatively small (Fig. 4e and Supplementary Fig. 7d). These results indicate that LEN-induced lattice flattening is associated with local remodeling of the intrahexamer NTD–CTD interface and structural rearrangements at the interhexamer trimer interface.

### LEN-containing cores exhibit reduced integrity

**Fig. 5:**
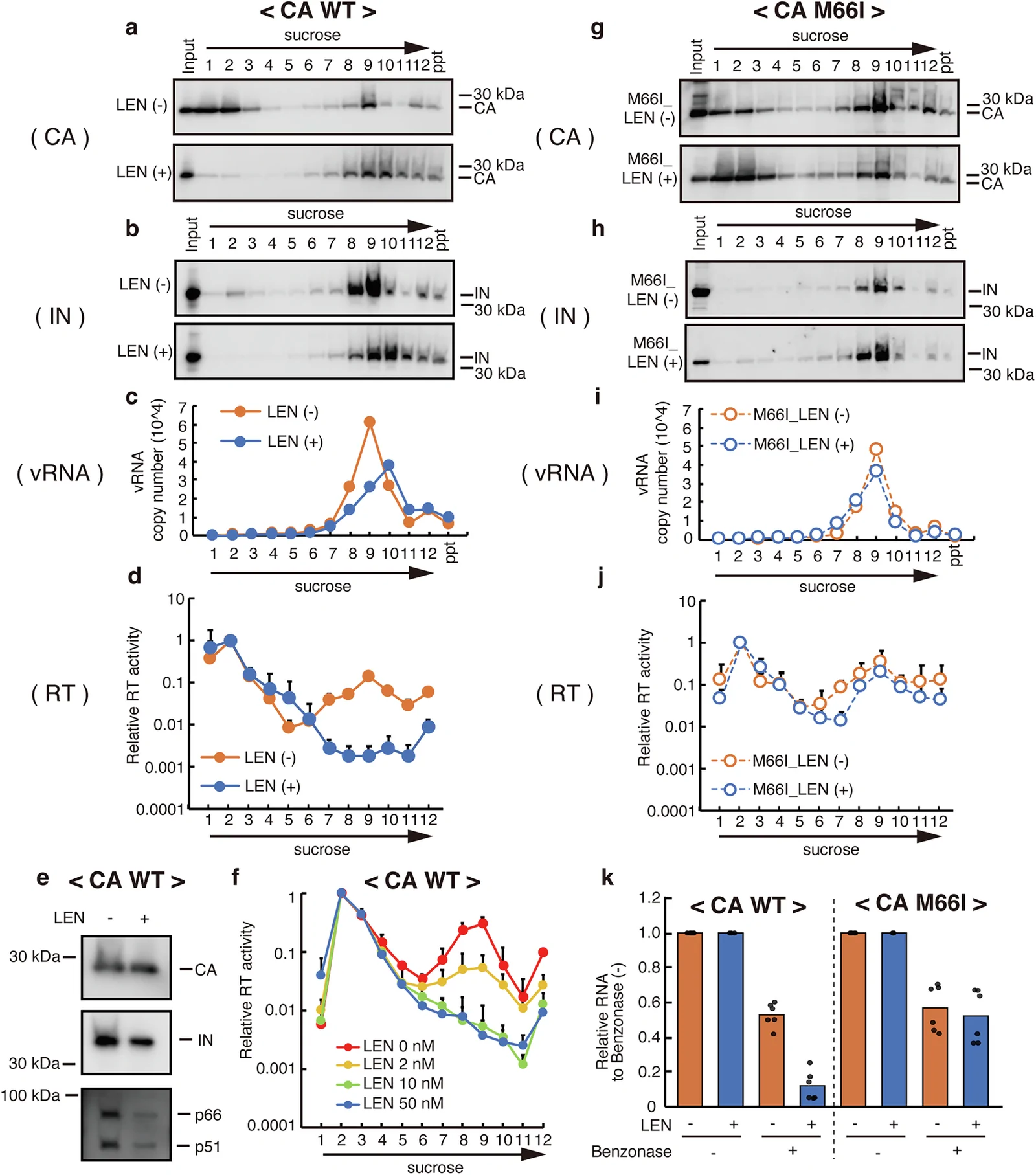
LEN-containing cores in HIV-1 virions exhibit reduced integrity and a loss of reverse transcriptase. **a**–**c** Sucrose gradient fractionation of HIV-GFP virions produced in the presence or absence of LEN. CA (**a**) and IN (**b**) were detected by western blotting. The amount of viral RNA in each fraction was quantified by RT–qPCR (**c**). Three independent experiments were repeated, and the results were reproduced. **d** Reverse transcriptase (RT) activity across sucrose gradient fractions. Red and blue lines indicate HIV-GFP produced without or with LEN, respectively. The data were normalized to fraction 2 of each virion sample. The data are presented as the means ± s.d.; Three independent experiments. **e** Western blot analysis of RT and IN levels in peak core fractions from LEN-free and LEN-containing virions. Peak fractions were loaded after normalization to equivalent p24 levels. Two independent experiments were repeated, and the results were reproduced. **f** Reverse transcriptase (RT) activity across sucrose gradient fractions of HIV-GFP virions in the absence or presence of LEN. Red, yellow, green, and blue lines represent HIV-GFP produced without LEN or with LEN (2, 10, or 50 nM), respectively; the data were normalized to fraction 2 of each virion sample and are shown as the means ± s.d. (Three independent experiments). **g**–**i** Sucrose gradient fractionation of M66I HIV-GFP virions produced in the presence or absence of LEN. CA (**g**) and IN (**h**) levels were detected by western blotting. The amount of viral RNA in each fraction was quantified by RT–qPCR (**i**). Three independent experiments were repeated, and the results were reproduced. **j** RT activity across sucrose gradient fractions. Red and blue dashed lines indicate the corresponding M66I mutants produced without or with LEN, respectively. The data were normalized to fraction 2 of each virion sample. The data are presented as the means ± s.d.; Three independent experiments. **k** Nuclease digestion assays using cores isolated from the peak fractions (**a**–**d**, **g**–**j**). Viral RNA levels are shown relative to those in samples that were not treated with benzonase. The experiment was performed twice independently, with three replicate reactions in each experiment.

Given the observed lattice-level perturbations, we next examined whether LEN altered the biochemical properties of the assembled HIV-1 cores. Cores were isolated from HIV-1 virions produced in the presence or absence of LEN using sucrose gradient ultracentrifugation containing IP6. Western blotting for CA revealed that cores extracted from the virion produced in the absence of LEN were deposited mainly in fraction 9, whereas those from LEN-containing virions peaked in fraction 10 (Fig. 5a). These results indicate that cores were formed under both conditions. In addition, the CA content in the light fractions (1–3), representing either CA that dissociated from the lattice during the assay or CA that was incorporated into virions but not assembled into the lattice, was markedly reduced in the presence of LEN compared with its absence (Fig. 5a). These findings indicate that LEN stabilizes the hexameric lattice of cores, which is consistent with previous reports^28,31^. The peaks of integrase (IN) and viral RNA overlapped with those of the cores under both conditions, indicating that IN and viral RNA were retained within the core of the virion produced in the presence of LEN (Fig. 5b, c and Supplementary Fig. 8). We next assessed the distribution of reverse transcriptase (RT) by measuring RT activity across gradient fractions. Unlike IN and viral RNA, RT was not present in LEN-containing cores, with RT activity in the peak fraction of untreated virions (fraction 9) being approximately 76-fold higher than that in the peak fraction of LEN-treated virions (fraction 10) (Fig. 5d and Supplementary Fig. 9). This marked reduction indicates that RT leaks from LEN-bound capsids. To further validate the loss of RT from LEN-containing cores at the protein level, we analysed the peak core fractions from LEN-free and LEN-containing virions by western blotting after normalizing the input to p24 levels. Under these p24-normalized conditions, IN was still readily detected in the LEN-containing core fraction, whereas RT was markedly depleted (Fig. 5e). These results corroborate the RT activity measurements and indicate that LEN-containing cores preferentially lose RT while retaining substantial amounts of IN. Furthermore, RT leakage from cores was already evident in virions produced in the presence of 2 nM LEN, with similar levels observed in the presence of 10 and 50 nM LEN (Fig. 5f). We next tested LEN in HIV-1 bearing the CA M66I mutation, which confers resistance to LEN^23,27,35^, to determine whether this LEN-dependent loss of RT is specifically mediated through the FG pocket of CA in the virion. Importantly, HIV-1 bearing the CA M66I mutation retained not only IN and viral RNA but also RT within the cores, even in the presence of LEN (Fig. 5g–j), demonstrating that RT leakage is specifically due to LEN binding to the FG pocket of CA within the virion. Previous studies have shown that IN binds tightly to viral RNA, serving as a key factor in virion maturation by ensuring proper viral RNA incorporation into the core^36,37^. Furthermore, destabilization of the capsid lattice has been reported to cause the loss of RT but not IN^38^. Together, these observations indicate that RT is more susceptible than IN to loss from cores lacking integrity and that the leakage of RT from LEN-containing cores is a clear indicator of compromised capsid integrity.

To further assess the integrity of LEN-containing cores, nuclease digestion assays were performed using cores isolated from the peak sucrose gradient fractions (Fig. 5a–d, g–j). These assays revealed that the viral RNA was more accessible within LEN-treated cores than in LEN-free cores, whereas M66I cores showed no such change (Fig. 5k).

### Cryo–ET reveals capsid defects in LEN-containing virions

**Fig. 6:**
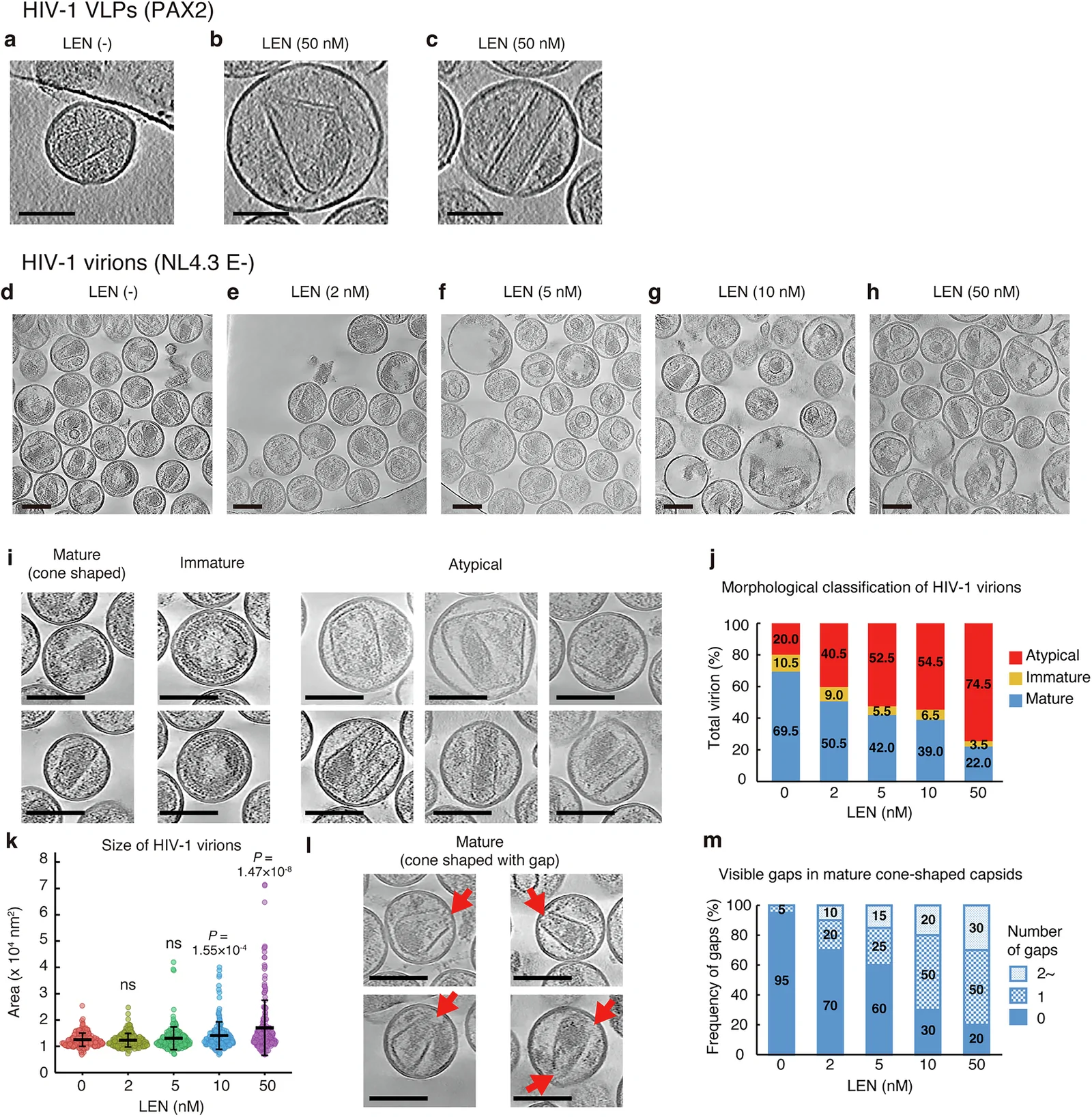
Cryo-electron tomography reveals capsid defects and impaired core integrity in LEN-containing virions. **a–c** Cryo-ET analysis of the Gag VLPs produced in the absence (**a**) or presence of 50 nM LEN (**b**, **c**). Representative tomogram slices of the Gag VLPs produced in the presence of 50 nM LEN (**b**, **c**) show partially disrupted cores, including sheet-like and tubular structures with unclosed ends. Scale bars, 100 nm. Two independent experiments were repeated, and the results were reproduced. **d–h** Cryo-ET analysis of HIV-1 virions produced in the absence (**d**) or presence of 2 nM (**e**), 5 nM (**f**), 10 nM (**g**), or 50 nM (**h**) LEN. Scale bars, 100 nm. **i** Representative tomogram slices of virions used for the morphological classification. Virions were classified into three morphological categories: mature, immature, and atypical capsid morphologies. The atypical category included particles with multiple cores, tubular cores, rod-shaped cores, sheet-like cores, or other aberrant capsid structures. **j** Quantitation of virions belonging to the indicated morphological categories shown in (**d**–**h**). More than 100 virions were counted for each condition. **k** Size distribution of virions produced in the absence or presence of LEN, as measured from the tomogram slices shown in (**d**–**h**). Each dot represents an individual virion (*n* = 200 per group), and horizontal bars indicate the means ± s.d. *P* values were calculated using unpaired two-tailed Welch’s t test. **l** Representative tomogram slices of virions containing mature cone-shaped capsids with gaps. Immature and atypical particles were excluded from this analysis. The arrows indicate visible gaps in the capsid lattice. Scale bars, 100 nm. **m** Quantification of visible gaps in apparently mature cone-shaped capsids after the exclusion of immature and atypical particles. Twenty randomly selected cores were analysed per condition.

To directly assess the structural integrity of capsids within VLPs, we analysed the VLPs produced in the absence and presence of LEN using cryo-electron tomography (cryo-ET) (Fig. 6a–c and Supplementary Movies 1–3). Although VLPs produced without LEN predominantly contained well-encapsulated cone-shaped capsids (Fig. 6a and Supplementary Movie 1), those produced in the presence of LEN frequently displayed abnormal structures, including extended sheet-like lattices that failed to curl into closed cones and tubular structures lacking properly sealed ends (Fig. 6b, c and Supplementary Movies 2 and 3). These defective structures, in stark contrast to the fully closed conical cores observed without LEN, provide direct structural evidence that LEN impairs the ability of the capsid lattice to assemble into an intact, encapsidated core. Next, we determined how these morphological defects depend on the LEN concentration by performing an additional cryo-ET analysis using NL4-3E− virions produced in the presence of 0, 2, 5, 10, or 50 nM LEN (Fig. 6d–h and Supplementary Movies 4–8). The particles were classified into three broad morphological categories: mature, immature, and atypical capsid morphologies (Fig. 6i). The atypical category included particles with multiple cores, tubular cores, rod-shaped cores, sheet-like cores, or other aberrant capsid structures, which were not always clearly distinguishable from one another and were therefore grouped together as capsids with atypical morphologies (Fig. 6i). In the absence of LEN, 69.5% of the virions contained mature cone-shaped capsids, whereas 10.5% and 20% showed immature and atypical morphologies, respectively (Fig. 6j). LEN treatment reduced the proportion of mature cone-shaped capsids and increased the proportion of capsids with atypical morphologies in a concentration-dependent manner (Fig. 6j). Even at the lowest concentration tested, 2 nM LEN, the proportion of capsids with atypical morphologies increased to 40.5%. At a pharmacologically relevant concentration of 5 nM LEN, 52.5% of the virions exhibited atypical morphologies, and this percentage further increased to 74.5% at 50 nM LEN. In addition, LEN treatment increased the virion size distribution, with larger particles appearing more frequently in a LEN concentration-dependent manner (Fig. 6k and Supplementary Table 3). These analyses demonstrate that LEN induces a concentration-dependent increase in atypical capsid structures and alters the virion size distribution, with morphological defects already detectable at 2–5 nM LEN. We further evaluated whether LEN compromises capsid integrity even among particles classified as mature by restricting the analysis to virions containing apparently mature cone-shaped capsids and excluded immature and atypical particles. We then quantified visible gaps in the capsids (Fig. 6l). As a result, the proportion of mature cone-shaped capsids containing gaps increased in a LEN concentration-dependent manner (Fig. 6m). Notably, at 10 nM LEN, approximately 70% of these cone-shaped capsids contained detectable gaps. These visible gaps were frequently observed around high-curvature regions (Fig. 6l), indicating that LEN can compromise capsid integrity even when the overall mature capsid morphology is retained. Together, these findings demonstrate that LEN binding within the viral core induces a flattened lattice geometry that compromises core integrity, thereby providing a mechanistic basis for its late-phase antiviral activity.

### Structural perturbations impair HIV-1 infectivity

**Fig. 7:**
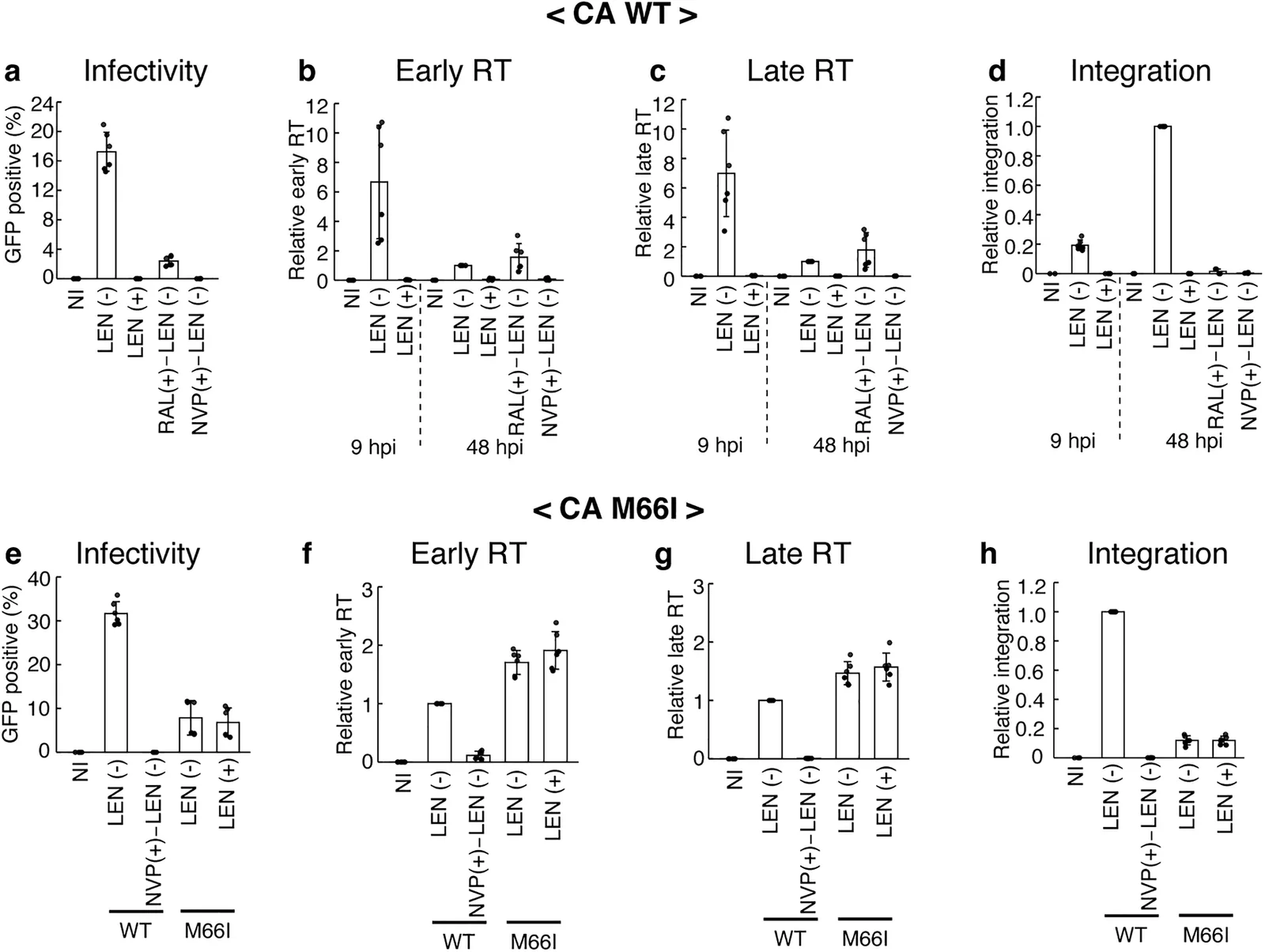
HIV-1 produced in the presence of LEN exhibits reduced infectivity and impaired reverse transcription. **a**–**d** Infectivity, reverse transcription (RT) products, and integration in Jurkat cells infected with HIV-GFP virions produced in the presence or absence of 50 nM LEN. Infectivity was quantified by measuring GFP expression at 48 h postinfection (hpi) (**a**). Early RT (**b**), late RT (**c**), and integration (**d**) products were quantified at 9 or 48 hpi. Raltegravir (RAL, 10 µM) and nevirapine (NVP, 10 µM) were included as controls. **e**–**h** Infectivity, RT products, and integration in Jurkat cells infected with HIV-GFP M66I-mutant virions produced in the presence or absence of 50 nM LEN, alongside WT and NVP controls, as shown in the figure. Infectivity was quantified at 48 hpi (**e**). Early RT (**f**), late RT (**g**), and integration (**h**) products were quantified at 48 hpi. The data are presented as the means ± s.d. from *n* = 6 independently treated cell cultures.

Finally, we sought to determine whether these structural and biochemical perturbations translate to functional defects during HIV-1 replication. First, we investigated the impact of LEN on HIV-1 infectivity using a VSV-G-pseudotyped HIV-GFP virus produced in the presence of LEN. Consistent with the findings of previous studies^27,31^, the percentage of GFP-positive cells decreased to 0.001% following infection with HIV-GFP produced in the presence of LEN, compared with 17% following infection with viruses produced in the absence of LEN, corresponding to an approximately 10,000-fold reduction in infectivity (Fig. 7a). To minimize the possibility that residual free LEN carried over into the infection assay, we subjected the virions to a second sucrose-cushion wash before infection (Supplementary Fig. 10). Even after this additional wash, the virions produced in the presence of LEN exhibited almost no infectivity (Supplementary Fig. 10b). Next, we analysed HIV-1 DNA synthesis, including early reverse transcription (early RT), late reverse transcription (late RT) and integration products (Fig. 7b–d and Supplementary Fig. 11), at 9 and 48 h postinfection (hpi). At both 9 and 48 hpi, the levels of early RT, late RT, and integration products in cells infected with HIV-GFP produced in the presence of LEN were indistinguishable from those in negative control cells infected with virus in the presence of nevirapine, indicating a near complete blockade of viral DNA synthesis (Fig. 7b–d). In contrast, LEN did not alter the infectivity, HIV-1 DNA synthesis, or integration of HIV-GFP bearing the capsid M66I mutation, which confers resistance to LEN^23,27,35^ (Fig. 7e–h). These results support the idea that the antiviral effect of LEN is attributable to its binding to the FG pocket in the capsid within the virion. Notably, the amount of early RT products was markedly reduced in cells infected with LEN-containing HIV-1. These results support the conclusion that LEN-induced lattice flattening leads to RT loss from the core and thereby prevents the efficient initiation of reverse transcription.

## Discussion

LEN is a long-acting HIV-1 capsid inhibitor and represents one of the most impactful antiviral agents currently in clinical use. Most previous studies have focused on the action of the capsid inhibitor LEN during early post-entry steps, where it modulates host factor interactions with the capsid and inhibits reverse transcription, nuclear import, and integration^26–30^. In these studies, the potency of LEN has largely been attributed to its ability to disrupt capsid function after infection. However, how LEN perturbs the capsid during the late phase of HIV-1 replication, specifically during virion maturation, has remained unresolved. Here, we address this key gap by integrating structural, biochemical, and functional analyses to define how LEN incorporation during virion production alters the capsid architecture and compromises viral infectivity. Our results reveal that LEN enforces a flattened lattice geometry by remodeling the intrahexamer NTD–CTD interface and inducing structural rearrangements at the interhexamer trimer interface. This lattice remodeling reduces core integrity and prevents productive reverse transcription and infection. Based on these observations, our data collectively suggest a sequential mechanistic model that links LEN incorporation into assembling virions to subsequent HIV-1 replication failure. Upon incorporation into the maturing virion, LEN binds to the FG pocket at the interface between the CA NTD and CTD, inducing a rearrangement of the interhexamer geometry and enforcing a flattened lattice conformation. This LEN-induced lattice flattening disrupts the precise spatial organization required for capsid closure, thereby compromising core integrity. As a consequence, cores formed in the presence of LEN prematurely lose RT while still retaining IN and viral RNA and thus fail to support reverse transcription and integration. Therefore, the cascade of structural perturbations triggered by LEN incorporation impairs the HIV-1 replication cycle.

In addition to binding the mature capsid, LEN also interacts with the immature Gag lattice in a distinct manner^27,32^. LEN binding induces conformational changes in the CA region and remodels the architecture of the Gag lattice^32^, which may interfere with the maturation process independently of proteolytic processing of Gag and result in the formation of aberrant capsid structures. A structural analysis of the capsid lattice within VLPs produced in the presence of LEN revealed how LEN disrupts the structure of the capsid, demonstrating that LEN binding within the viral core enforces a flattened lattice geometry that compromises capsid integrity. In addition, a previous coarse-grained molecular dynamics study revealed that LEN-treated capsids frequently fail to encapsidate and exhibit impaired maturation^39^. Furthermore, while LEN has been shown to impair the formation of CA pentamers^31^, which are critical for generating a local high curvature and assembling an encapsidated core, our structural analysis indicates that LEN enforces the formation of flattened CA hexamer lattices. Together, these findings suggest that both mechanisms contribute to the production of nonfunctional HIV-1 particles with aberrant capsid structures and thereby inhibit the late steps of HIV-1 replication, providing a mechanistic basis for the profound antiviral efficacy of the clinically approved capsid inhibitor LEN.

Although a pharmacologically relevant concentration of LEN has been reported to be approximately 5 nM^28^, our structural analysis was performed using VLPs produced in the presence of 50 nM LEN (Fig. 1) to ensure near-complete lattice occupancy. Notably, a previous study reported structural abnormalities in HIV-1 virions produced in the presence of 15 nM LEN^27^. Importantly, in our biochemical assays using isolated viral cores, significant RT leakage was observed from cores produced in the presence of 2 nM LEN (Fig. 5f). Consistent with these observations, our cryo-ET analysis of virions revealed capsid morphological defects, including gaps even in the cone-shaped capsids, with detectable effects observed with 2–5 nM LEN (Fig. 6d–m). These findings suggest that even partial LEN occupancy, and consequently partial lattice flattening, is sufficient to compromise capsid integrity.

In addition, LEN treatment increased the fraction of larger particles and broadened the particle size distribution. These findings suggest that LEN may also perturb Gag assembly or particle formation during virion production. However, this effect was heterogeneous and was not observed uniformly across all the particles, in contrast to the more consistent increase in capsid morphological defects. These findings suggest that LEN may affect virion production at multiple levels, with heterogeneous effects on virion size and more consistent effects on the capsid architecture and core integrity. Because LEN was present throughout virus production in our experiments, our data do not completely distinguish whether the observed capsid defects arose during capsid assembly, during cone closure, or immediately after the formation of the mature capsid. Therefore, LEN-induced lattice flattening may impair both capsid closure and the subsequent maintenance of core integrity.

Previous studies using purified mature virion cores have shown that LEN disrupts capsid integrity despite stabilizing the capsid lattice^29,30^. These reports further indicate that capsid disruption is non-uniform. Specifically, the narrow ends of viral cores, which contain clustered pentamers and exhibit a greater intrinsic curvature, were preferentially destabilized by the treatment of LEN^30^. Although LEN is thought to bind only to CA hexamers and not to pentamers^15,40,41^, rupture is predominantly initiated at the narrow ends, suggesting that the LEN-induced perturbation of the interhexamer geometry preferentially affects high-curvature regions of the lattice. A recent study further reported the curvature-dependent destabilization of mature HIV-1 capsids by LEN in post-entry cores^42^. Consistent with these observations, our results show that LEN reduces the local lattice curvature during virion maturation, perturbing curvature establishment prior to complete cone closure. Notably, our cryo-ET analysis further showed that LEN can compromise capsid integrity even when the overall mature cone-shaped morphology is retained, as visible gaps were frequently observed in the capsid lattice, particularly around high-curvature regions, even at subsaturating LEN concentrations. These observations support a model in which partial lattice flattening induced by subsaturating LEN concentrations during viral maturation is sufficient to impair the proper formation of high-curvature regions, thereby preventing complete cone closure and productive encapsidation.

A recent study revealed that LEN allosterically remodels properly matured HIV-1 capsids by altering the noncovalent interactions between capsid subunits and reducing the local lattice curvature^42^. These findings are consistent with our observations that LEN induces capsid lattice flattening and compromises the capsid architecture. However, the two studies address distinct stages of the HIV-1 life cycle. Although the recent study revealed the effects of LEN on preformed mature cores, our work shows that LEN incorporation during virion production alters interhexamer interactions, perturbs the capsid lattice curvature, and prevents the formation of a functional cone-shaped capsid. Therefore, LEN can compromise capsid integrity not only by fracturing already matured capsids but also by preventing the formation of properly closed cores during virion maturation.

Our findings further suggest that capsid curvature is a tuneable geometric parameter that can be pharmacologically regulated. Rather than simply hyperstabilizing the capsid lattice, LEN alters the interhexamer tilt without disrupting hexamer integrity, thereby biasing assembly towards flattened, non-closable capsid intermediates. This geometric derailment model highlights curvature control as a structural vulnerability of retroviral assembly.

## Methods

### Plasmids

pNL4-3 was obtained from the NIH AIDS Reagent Program. pNL4-3 E- was generated from pNL4-3 using a Q5 site-directed mutagenesis kit (NEB). Briefly, two nucleotides were inserted at the NdeI site within the env gene to introduce a frameshift mutation and abolish Env expression. psPAX2 and pMD2.G were gifts from Didier Trono (Addgene plasmids #12260 and #12259). The construct pNL4-3.GFP.R + E-, which was generated from pNL4-3.Luc.R-E- by replacing the luciferase reporter with GFP and restoring Vpr expression, was kindly provided by M. Benkirane.

### Virus production

HIV-1 Env^-^ VLPs were produced by transfecting HEK293T Lenti-X cells (Takara/Clontech, 632180), which were confirmed to be mycoplasma free, with 4–8 µg of psPAX2 using polyethyleneimine (PEI Max) in the presence of 50 nM lenacapavir (LEN; MedChemExpress, HY-111964). HIV-1 Env^-^ virions were produced by transfecting cells with 8 µg of pNL4-3. E- using PEI Max in the presence of 2, 5, 10, or 50 nM LEN. VSV-G-pseudotyped HIV-1-GFP was generated by cotransfecting cells with 4 µg of pNL4-3.GFP.R + E- and 1 µg of pMD2-G using PEI Max in the presence of 50 nM LEN. Sixteen hours later, the medium was replaced with DMEM supplemented with 1% FBS supplemented with 50 nM LEN. In experiments assessing the dependence of the effects on the LEN concentration, the medium was replaced with DMEM supplemented with 1% FBS and 2, 5, 10, or 50 nM LEN. At 48 h post-transfection, the medium containing the VLPs and virions was harvested, filtered through a 0.45-µm filter, and concentrated by ultracentrifugation through a 20% sucrose cushion. The viral pellet was collected and treated with 100 U/mL DNase (Turbo DNase; Thermo Fisher Scientific) at 37 °C for 1 h. For HIV-1 Env^-^ VLPs and HIV-1 Env^-^ virions used for the cryo-EM analysis, VLPs and virions were further purified using an OptiPrep gradient containing 10, 20, and 30% OptiPrep (Serumwerk Bernburg AG) in STE (10 mM Tris-HCl (pH 7.4), 100 mM NaCl, and 1 mM EDTA) from top to bottom. Ultracentrifugation was performed at 246,100 × *g* (SW 55Ti rotor) for 2.5 h at 4 °C. The band corresponding to the VLPs and virions was collected and concentrated by ultracentrifugation through an 8% OptiPrep cushion at 214,400 × *g* (SW 55Ti rotor) for 1 h at 4 °C.

### Quantification of infectivity and viral DNA

Jurkat cells (ATCC, TIB-152), which were confirmed to be mycoplasma free, were infected with VSV-G-pseudotyped HIV-1-GFP produced in the presence or absence of LEN. The multiplicity of infection (MOI) was set to 0.3, as determined by the percentage of GFP-positive Jurkat cells infected with VSV-G-pseudotyped HIV-1-GFP produced in the absence of LEN. For infections with viruses produced in the presence of LEN, equivalent amounts of viral RNA to that of the LEN-free virus were used. After 3 h, the cells were washed twice with PBS, and the culture medium was replaced. At 48 h postinfection (hpi), the cells were collected, fixed with 4% paraformaldehyde, and subjected to a flow cytometry analysis using a BD FACSVerse (BD Biosciences). GFP-positive cells were detected using a 488 nm blue laser and the FITC/GFP detection channel with a 527/32 nm bandpass filter. The data were analysed using BD FACSuite software. Flow cytometry gating strategy was shown in Supplementary Fig. 12. For viral DNA quantification, DNA was extracted from infected cells at 9 or 48 hpi using a NucleoSpin Tissue Kit (Takara/Clontech) and subjected to qPCR. For the quantification of early RT and late RT products, 12.5 ng of DNA (quantified using a Qubit) was used in each reaction^43–45^. Integrated HIV DNA was quantified by the Alu‒PCR method, as described previously^44,46,47^, using 12.5 ng of DNA (quantified using a Qubit). Early RT, late RT and integrated HIV DNA concentrations were normalized to β-globin concentrations. The primers used for viral DNA quantification are listed in Supplementary Table 4.

### Sucrose gradient fractionation of HIV-1 cores

VSV-G pseudotyped HIV-1-GFP particles produced in the presence or absence of LEN were subjected to sucrose gradient fractionation as previously described, with slight modifications^30^. Viral input was normalized across samples based on the viral RNA levels and quantified by qPCR using primers specific for the late RT product prior to loading. Virions were subjected to ultracentrifugation through a 1% Triton X-100 layer into a linear 30–70% sucrose density gradient containing 10 ml of 10 mM HEPES (pH 7.5), 150 mM NaCl, and 200 μM IP6 (Sigma). The samples were applied to the top of the gradient and centrifuged at 129,700 × *g* for 16 h at 4 °C using a Beckman SW40Ti rotor. Following centrifugation, 1 ml fractions were collected sequentially from the top of the gradient. CA, IN, and RT were detected in each fraction by western blotting using serum or antibodies against CA (NIH AIDS Reagent Program, Cat# 4250, 1:1000 dilution), IN (Santa Cruz, sc-69721, 1:500 dilution), and RT (Abcam, ab63911, 1:1000 dilution). For the quantification of viral RNA in each fraction, RNA was extracted using the NucleoSpin RNA Virus Kit (Takara/Clontech). Reverse transcription was then performed, followed by qPCR using primers specific for late RT. RT activity in each fraction was quantified using a qPCR-based product-enhanced reverse transcriptase (PERT) assay, as previously described^48,49^.

### Nuclease sensitivity assay

Five microlitres of the peak fraction from the sucrose gradient was diluted fivefold with 20 µl of buffer used for the sucrose gradient that lacked sucrose and treated with 50 U/ml benzonase (Merck Millipore) in the presence of 5 mM MgCl₂. After an incubation at 37 °C for 30 min, the reaction was stopped by the addition of 10 mM EDTA. Viral RNA was then purified using the NucleoSpin RNA Virus Kit (Takara/Clontech). Reverse transcription was performed, followed by qPCR using primers specific for late RT.

### Cryo-EM structure determination

#### Cryo-EM sample preparation

The VLPs treated with PFO were prepared by incubating the VLPs with 0.1 mg/ml PFO (CUSABIO Technology LLC) and 200 μM IP6 (Sigma) at room temperature for 2 h. The NL4-3E− virions were prepared by fixing them with 1% paraformaldehyde on ice for 1 h. For the preparation of the cryo-EM sample, Quantifoil grids (R2/2, 200-mesh, copper; Quantifoil Micro Tools GmbH) were pretreated with acetone and glow-discharged with a PIB-10 system (Vacuum Device). Two-microlitre samples were applied to the grids, and the grids were then blotted at 100% humidity and 18 °C in a Vitrobot Mark IV System (Thermo Fisher Scientific) or EM GP2 (Leica Microsystems). The blotted grids were immediately plunged into liquid ethane.

#### CryoEM SPA data collection

Cryo-EM images for SPA were acquired on a Krios G4 (Thermo Fisher Scientific) transmission electron microscope operated at 300 kV and equipped with either a K3 direct electron detector (Gatan) and a GIF-BioContinuum energy filter (Gatan) or a Falcon 4i direct electron detector (Thermo Fisher Scientific) and a Selectris energy filter (Thermo Fisher Scientific). Automated data collection was performed with EPU software (Thermo Fisher Scientific). The data collection parameters are summarized in Supplementary Table 2.

#### Cryo-EM SPA image processing

All image processing procedures were performed with cryoSPARC^50^. Micrograph movie frames were aligned, the dose was weighted, and the CTF was estimated using patch motion correction and patch CTF in cryoSPARC. The particle images were automatically picked with a Blob picker. Junk particles were then removed by 2D classification.

For the LEN-bound and LEN-unbound HIV-1 capsid lattices within VLPs treated with PFO, particles were initially extracted with a box size of 270 pixels and binned by a factor of 3. The particles were further classified by one round of heterogeneous refinement with C6 symmetry imposed, using either ab initio 3D templates or the intact mature CA hexamer (EMD-3465) as the initial template. Particles assigned to the best-resolved class were then selected, re-extracted, and subjected to non-uniform refinement. Next, particles exposing the capsid from ruptured virions were manually excluded. The remaining particles were subjected to reference-based motion correction, global and local CTF refinement, and non-uniform refinement with C6 symmetry imposed. The particles were re-extracted with a box size of 384 pixels and subjected to non-uniform refinement with C1 symmetry to determine the structure without imposing symmetry. The heterogeneity in the tilt angles was assessed and the particles were further classified by subjecting the selected particles to 3D classification.

For the LEN-bound HIV-1 capsid lattice within intact VLPs, particles were extracted with a box size of 270 pixels. The structure of the intact mature CA hexamer (EMD-3465) and decoy maps derived from a 100 Å lowpass-filtered CA hexamer map (EMD-3465) were used as the initial 3D model for the first round of 3D heterogeneous refinement. Iterative rounds of heterogeneous refinement and 2D classification were performed to remove junk particles. Then, particles exposing the capsid from ruptured virions were manually excluded. The selected particles were subjected to reference-based motion correction, global CTF refinement, local CTF refinement, and non-uniform refinement.

The global resolutions were based on the gold-standard Fourier shell correlation curve (FSC = 0.143) criteria. The workflows for data processing are shown in Supplementary Fig. 2 (the LEN-bound HIV-1 capsid lattice within VLPs treated with PFO), Supplementary Fig. 4 (the LEN-unbound HIV-1 capsid lattice within VLPs treated with PFO), and Supplementary Fig. 6 (the LEN-bound HIV-1 capsid lattice within intact VLPs).

#### Model building and refinement

The initial atomic models of HIV-1 CA (PDB: 4XFX) were rigid bodies fitted to the cryo-EM map using UCSF Chimera and ChimeraX^51,52^. The models were subjected to automatic refinement with phenix.real_space_refine^53^ and manually edited with Coot^54^. The models were validated with the MolProbity tool^55^.

#### Measurement of the tilt angle of the capsid lattice

Tilt angles were measured with reference to a previously described method^15^. Capsid hexamer models were rigidly fitted to adjacent densities using UCSF Chimera. For each fitted hexamer model, a flat reference plane perpendicular to the symmetry axis was defined using the “Structure Measurements” tool in UCSF Chimera. Tilt angles were then calculated relative to this reference plane using the “angle” command in UCSF Chimera.

#### CryoET data collection

For the VLP data, cryo-ET tilt-series images were acquired using a Krios G4 (Thermo Fisher Scientific) transmission electron microscope operated at 300 kV and equipped with a K3 direct electron detector (Gatan) and a GIF-BioContinuum energy filter (Gatan) with a 20-eV slit width. Each image was collected at a nominal magnification of 26,000 (pixel size of 3.37 Å), with a defocus range of −6 to −8 µm and an electron dose of 2.53 e^-^/Å^2^. Tilt-series data collection was performed with a dose-symmetric scheme starting from 0° with a 3° tilt increment and an angular range of ±60° using Tomography 5 software (Thermo Fisher Scientific).

For the NL4-3E− virion data, cryo-ET tilt-series images were acquired using a Krios G4 (Thermo Fisher Scientific) operated at 300 kV and equipped with a Falcon 4i direct electron detector (Thermo Fisher Scientific) and a Selectris energy filter (Thermo Fisher Scientific) with a 10-eV slit width. Each image was collected at a nominal magnification of 64,000 (pixel size of 1.9 Å), with a defocus range of −2 to −4 µm and an electron dose of 4 e^-^/Å^2^. Tilt-series data were acquired using a dose-symmetric scheme starting from 0°, with a 3° tilt increment over an angular range of ±50°, using Tomography 5 software (Thermo Fisher Scientific).

#### CryoET data processing

For VLP data processing, tilt-series images were processed using AreTomo2 software^56^. The tomograms were binned 4x and reconstructed with weighted back projection. Reconstructed tomograms were also imported and improved using IsoNet software^57^. Reconstructed volumes were visualized with the IMOD package^58^.

For NL4-3E− virion data processing, tilt-series images were processed using AreTomo3 software (https://github.com/czimaginginstitute/AreTomo3). The tomograms were binned 8x and reconstructed with weighted back projection. The reconstructed tomograms were further denoised using cryoCARE software^59^. Reconstructed volumes were visualized using the IMOD package and Fiji^60^.

### Reporting summary

Further information on research design is available in the Nature Portfolio Reporting Summary linked to this article.

## Supplementary information

Supplementary Information

Description of Additional Supplementary Files

Supplementary Movie 1

Supplementary Movie 2

Supplementary Movie 3

Supplementary Movie 4

Supplementary Movie 5

Supplementary Movie 6

Supplementary Movie 7

Supplementary Movie 8

Reporting Summary

Transparent Peer Review file

## Source data

Source Data

## Data Availability

Cryo-EM maps generated in this study have been deposited in the Electron Microscopy Data Bank (EMDB) under the accession codes EMD-81945 (LEN-bound HIV-1 capsid lattice within VLPs treated with PFO, C6 symmetry), EMD-81946 (LEN-bound HIV-1 capsid lattice within VLPs treated with PFO, no symmetry), EMD-81947 (LEN-unbound HIV-1 capsid lattice within VLPs treated with PFO, C6 symmetry), EMD-81948 (LEN-unbound HIV-1 capsid lattice within VLPs treated with PFO, no symmetry) and EMD-67116 (LEN-bound HIV-1 capsid lattice within intact VLPs). The coordinates generated in this study have been deposited in the Protein Data Bank (PDB) under accession codes 43KN (LEN-bound HIV-1 capsid lattice within VLPs treated with PFO), 43KO (LEN-unbound HIV-1 capsid lattice within VLPs treated with PFO), and 9XQH (LEN-bound HIV-1 capsid lattice within intact VLPs). The previously published structural data used in this study are available from the PDB and EMDB under the following accession codes: PDB 4XFX, EMD-3465, EMD-13423, EMD-16703. Source data are provided as a Source Data file. Source data are provided with this paper.

## References

[1] Campbell, E. M. & Hope, T. J. HIV-1 capsid: the multifaceted key player in HIV-1 infection. *Nat. Rev. Microbiol.* **13**, 471–483 (2015). 26179359 10.1038/nrmicro3503 PMC4876022

[2] James, L. C. & Jacques, D. A. The human immunodeficiency virus capsid is more than just a genome package. *Annu. Rev. Virol.* **5**, 209–225 (2018). 30052493 10.1146/annurev-virology-092917-043430

[3] Temple, J., Tripler, T. N., Shen, Q. & Xiong, Y. A snapshot of HIV-1 capsid–host interactions. *Curr. Res. Struct. Biol.* **2**, 222–228 (2020). 34113849 10.1016/j.crstbi.2020.10.002 PMC8189282

[4] Shen, Q., Wu, C., Freniere, C., Tripler, T. N. & Xiong, Y. Nuclear import of HIV-1. *Viruses* **13**, 2242 (2021). 34835048 10.3390/v13112242 PMC8619967

[5] Müller, T. G., Zila, V., Müller, B. & Kräusslich, H.-G. Nuclear capsid uncoating and reverse transcription of HIV-1. *Ann. Rev. Virol.* **9**, 261–284 (2022). 35704745 10.1146/annurev-virology-020922-110929

[6] Briggs, J. A. G. & Kräusslich, H.-G. The molecular architecture of HIV. *J. Mol. Biol.* **410**, 491–500 (2011). 21762795 10.1016/j.jmb.2011.04.021

[7] Pornillos, O. & Ganser-Pornillos, B. K. Maturation of retroviruses. *Curr. Opin. Virol.* **36**, 47–55 (2019). 31185449 10.1016/j.coviro.2019.05.004 PMC6730672

[8] Krebs, A.-S., Mendonça, L. M. & Zhang, P. Structural analysis of retrovirus assembly and maturation. *Viruses* **14**, 54 (2021). 35062258 10.3390/v14010054 PMC8778513

[9] Ganser, B. K., Li, S., Klishko, V. Y., Finch, J. T. & Sundquist, W. I. Assembly and analysis of conical models for the HIV-1 Core. *Science* **283**, 80–83 (1999). 9872746 10.1126/science.283.5398.80

[10] Yu, A., Lee, E. M. Y., Jin, J. & Voth, G. A. Atomic-scale characterization of mature HIV-1 capsid stabilization by inositol hexakisphosphate (IP6). *Sci. Adv.* **6**, eabc6465 (2020). 32938668 10.1126/sciadv.abc6465 PMC7494349

[11] Li, S., Hill, C. P., Sundquist, W. I. & Finch, J. T. Image reconstructions of helical assemblies of the HIV-1 CA protein. *Nature* **407**, 409–413 (2000). 11014200 10.1038/35030177

[12] Briggs, J. A. G., Wilk, T., Welker, R., Kräusslich, H. & Fuller, S. D. Structural organization of authentic, mature HIV-1 virions and cores. *EMBO J.* **22**, 1707–1715 (2003). 12660176 10.1093/emboj/cdg143 PMC152888

[13] Mattei, S., Glass, B., Hagen, W. J. H., Kräusslich, H.-G. & Briggs, J. A. G. The structure and flexibility of conical HIV-1 capsids determined within intact virions. *Science* **354**, 1434–1437 (2016). 27980210 10.1126/science.aah4972

[14] Zhao, G. et al. Mature HIV-1 capsid structure by cryo-electron microscopy and all-atom molecular dynamics. *Nature* **497**, 643–646 (2013). 23719463 10.1038/nature12162 PMC3729984

[15] Stacey, J. C. V. et al. Two structural switches in HIV-1 capsid regulate capsid curvature and host factor binding. *Proc. Natl. Acad. Sci. USA*. **120**, e2220557120 (2023). 37040417 10.1073/pnas.2220557120 PMC10120081

[16] Mallery, D. L. et al. IP6 is an HIV pocket factor that prevents capsid collapse and promotes DNA synthesis. *Elife* **7**, e35335 (2018). 29848441 10.7554/eLife.35335 PMC6039178

[17] Dick, R. A. et al. Inositol phosphates are assembly co-factors for HIV-1. *Nature* **560**, 509–512 (2018). 30069050 10.1038/s41586-018-0396-4 PMC6242333

[18] Forshey, B. M., Schwedler, U. von, Sundquist, W. I. & Aiken, C. Formation of a human immunodeficiency virus type 1 core of optimal stability is crucial for viral replication. *J. Virol.* **76**, 5667–5677 (2002). 11991995 10.1128/JVI.76.11.5667-5677.2002 PMC137032

[19] Fricke, T., Brandariz-Nuñez, A., Wang, X., Smith, A. B. & Diaz-Griffero, F. Human cytosolic extracts stabilize the HIV-1 Core. *J. Virol.* **87**, 10587–10597 (2013). 23885082 10.1128/JVI.01705-13 PMC3807412

[20] Hulme, A. E., Kelley, Z., Okocha, E. A. & Hope, T. J. Identification of capsid mutations that alter the rate of HIV-1 uncoating in infected cells. *J. Virol.* **89**, 643–651 (2014). 25339776 10.1128/JVI.03043-14 PMC4301138

[21] Mamede, J. I., Cianci, G. C., Anderson, M. R. & Hope, T. J. Early cytoplasmic uncoating is associated with infectivity of HIV-1. *Proc. Natl. Acad. Sci.USA* **114**, E7169–E7178 (2017). 28784755 10.1073/pnas.1706245114 PMC5576815

[22] Márquez, C. L. et al. Kinetics of HIV-1 capsid uncoating revealed by single-molecule analysis. *Elife* **7**, e34772 (2018). 29877795 10.7554/eLife.34772 PMC6039174

[23] Segal-Maurer, S. et al. Capsid inhibition with lenacapavir in multidrug-resistant HIV-1 infection. *N. Engl. J. Med.* **386**, 1793–1803 (2022). 35544387 10.1056/NEJMoa2115542

[24] Ogbuagu, O. et al. Efficacy and safety of the novel capsid inhibitor lenacapavir to treat multidrug-resistant HIV: week 52 results of a phase 2/3 trial. *Lancet HIV* **10**, e497–e505 (2023). 37451297 10.1016/S2352-3018(23)00113-3

[25] Bekker, L.-G. et al. Twice-yearly lenacapavir or daily F/TAF for HIV prevention in cisgender women. *N. Engl. J. Med*. 10.1056/nejmoa2407001. (2024) 39046157

[26] Yant, S. R. et al. A highly potent long-acting small-molecule HIV-1 capsid inhibitor with efficacy in a humanized mouse model. *Nat. Med.* **25**, 1377–1384 (2019). 31501601 10.1038/s41591-019-0560-x PMC7396128

[27] Link, J. O. et al. Clinical targeting of HIV capsid protein with a long-acting small molecule. *Nature* **584**, 614–618 (2020). 32612233 10.1038/s41586-020-2443-1 PMC8188729

[28] Bester, S. M. et al. Structural and mechanistic bases for a potent HIV-1 capsid inhibitor. *Science* **370**, 360–364 (2020). 33060363 10.1126/science.abb4808 PMC7831379

[29] Faysal, K. R. et al. Pharmacologic hyperstabilisation of the HIV-1 capsid lattice induces capsid failure. *eLife* **13**, e83605 (2024). 38347802 10.7554/eLife.83605 PMC10863983

[30] Li, C. et al. Lenacapavir disrupts HIV-1 core integrity while stabilizing the capsid lattice. *Proc. Natl. Acad. Sci. USA* **122**, e2420497122 (2025). 40168125 10.1073/pnas.2420497122 PMC12002175

[31] Huang, S.-W. et al. The primary mechanism for highly potent inhibition of HIV-1 maturation by lenacapavir. *PLOS Pathog.* **21**, e1012862 (2025). 39869652 10.1371/journal.ppat.1012862 PMC11892807

[32] Wu, C. et al. Distinct target site of lenacapavir in immature HIV-1 and concurrent binding with the maturation inhibitor bevirimat. *J. Am. Chem. Soc.* **147**, 42685–42700 (2025). 41198571 10.1021/jacs.5c13735 PMC12900427

[33] Tilley, S. J., Orlova, E. V., Gilbert, R. J. C., Andrew, P. W. & Saibil, H. R. Structural basis of pore formation by the bacterial toxin pneumolysin. *Cell* **121**, 247–256 (2005). 15851031 10.1016/j.cell.2005.02.033

[34] Ni, T. et al. Structure of native HIV-1 cores and their interactions with IP6 and CypA. *Sci. Adv.* **7**, eabj5715 (2021). 34797722 10.1126/sciadv.abj5715 PMC8604400

[35] Briganti, L. et al. Structural and mechanistic bases for resistance of the M66I capsid variant to lenacapavir. *mBio* **16**, e03613-24 (2025). 40231850 10.1128/mbio.03613-24 PMC12077090

[36] Kessl, J. J. et al. HIV-1 integrase binds the viral RNA genome and is essential during virion morphogenesis. *Cell* **166**, 1257–1268.e12 (2016). 27565348 10.1016/j.cell.2016.07.044 PMC5003418

[37] Elliott, J. L. et al. Integrase-RNA interactions underscore the critical role of integrase in HIV-1 virion morphogenesis. *Elife* **9**, e54311 (2020). 32960169 10.7554/eLife.54311 PMC7671690

[38] Eschbach, J. E. et al. Capsid lattice destabilization leads to premature loss of the viral genome and integrase enzyme during HIV-1 Infection. *J. Virol*. **95**, 10.1128/jvi.00984-20 (2020). PMC7944438 33115869

[39] Gupta, M. et al. Mechanistic insights into lenacapavir-induced off-pathway HIV-1 capsid assembly. *Proc. Natl. Acad. Sci.USA* **123**, e2524995123 (2026). 41805577 10.1073/pnas.2524995123 PMC12991430

[40] Schirra, R. T. et al. A molecular switch modulates assembly and host factor binding of the HIV-1 capsid. *Nat. Struct. Mol. Biol.* **30**, 383–390 (2023). 36759579 10.1038/s41594-022-00913-5 PMC10023569

[41] Highland, C. M., Tan, A., Ricaña, C. L., Briggs, J. A. G. & Dick, R. A. Structural insights into HIV-1 polyanion-dependent capsid lattice formation revealed by single particle cryo-EM. *Proc. Natl. Acad. Sci.USA* **120**, e2220545120 (2023). 37094124 10.1073/pnas.2220545120 PMC10160977

[42] Santos, N. F. B. dos et al. Lenacapavir allosterically remodels the HIV-1 capsid. *Sci. Adv.* **12**, eaed8384 (2026). 10.1126/sciadv.aed8384 PMC13537256 42685215

[43] Brussel, A. & Sonigo, P. Analysis of early human immunodeficiency virus type 1 DNA synthesis by use of a new sensitive assay for quantifying integrated provirus. *J. Virol.* **77**, 10119–10124 (2003). 12941923 10.1128/JVI.77.18.10119-10124.2003 PMC224570

[44] Machida, S. et al. Exploring histone loading on HIV DNA reveals a dynamic nucleosome positioning between unintegrated and integrated viral genome. *Proc. Natl. Acad. Sci. USA* **117**, 6822–6830 (2020). 32161134 10.1073/pnas.1913754117 PMC7104181

[45] Christensen, D. E., Ganser-Pornillos, B. K., Johnson, J. S., Pornillos, O. & Sundquist, W. I. Reconstitution and visualization of HIV-1 capsid-dependent replication and integration in vitro. *Science* **370**, eabc8420 (2020). 33033190 10.1126/science.abc8420 PMC8022914

[46] Butler, S. L., Hansen, M. S. & Bushman, F. D. A quantitative assay for HIV DNA integration in vivo. *Nat. Med.* **7**, 631–634 (2001). 11329067 10.1038/87979

[47] Thenin-Houssier, S. et al. POLE3 is a repressor of unintegrated HIV-1 DNA required for efficient virus integration and escape from innate immune sensing. *Sci. Adv.* **9**, eadh3642 (2023). 37922361 10.1126/sciadv.adh3642 PMC10624344

[48] Khan, H. et al. HIV-1 Vpr antagonizes innate immune activation by targeting karyopherin-mediated NF-κB/IRF3 nuclear transport. *eLife* **9**, e60821 (2020). 10.7554/eLife.60821 PMC7759385 33300875

[49] Vermeire, J. et al. Quantification of reverse transcriptase activity by real-time PCR as a fast and accurate method for titration of HIV, lenti- and retroviral vectors. *PLoS ONE* **7**, e50859 (2012). 23227216 10.1371/journal.pone.0050859 PMC3515444

[50] Punjani, A., Rubinstein, J. L., Fleet, D. J. & Brubaker, M. A. cryoSPARC: algorithms for rapid unsupervised cryo-EM structure determination. *Nat. Methods* **14**, 290–296 (2017). 28165473 10.1038/nmeth.4169

[51] Pettersen, E. F. et al. UCSF Chimera—a visualization system for exploratory research and analysis. *J. Comput. Chem.* **25**, 1605–1612 (2004). 15264254 10.1002/jcc.20084

[52] Pettersen, E. F. et al. UCSF ChimeraX: structure visualization for researchers, educators, and developers. *Protein Sci.* **30**, 70–82 (2021). 32881101 10.1002/pro.3943 PMC7737788

[53] Afonine, P. V. et al. Real-space refinement in PHENIX for cryo-EM and crystallography. *Acta Crystallogr. Sect. D: Struct. Biol.* **74**, 531–544 (2018). 29872004 10.1107/S2059798318006551 PMC6096492

[54] Emsley, P., Lohkamp, B., Scott, W. G. & Cowtan, K. Features and development of Coot. *Acta Crystallogr. Sect. D: Biol. Crystallogr.* **66**, 486–501 (2010). 20383002 10.1107/S0907444910007493 PMC2852313

[55] Williams, C. J. et al. MolProbity: more and better reference data for improved all-atom structure validation. *Protein Sci.* **27**, 293–315 (2018). 29067766 10.1002/pro.3330 PMC5734394

[56] Zheng, S. et al. AreTomo: an integrated software package for automated marker-free, motion-corrected cryo-electron tomographic alignment and reconstruction. *J. Struct. Biol. X* **6**, 100068 (2022). 35601683 10.1016/j.yjsbx.2022.100068 PMC9117686

[57] Liu, Y.-T. et al. Isotropic reconstruction for electron tomography with deep learning. *Nat. Commun.* **13**, 6482 (2022). 36309499 10.1038/s41467-022-33957-8 PMC9617606

[58] Kremer, J. R., Mastronarde, D. N. & McIntosh, J. R. Computer visualization of three-dimensional image data using IMOD. *J. Struct. Biol.* **116**, 71–76 (1996). 8742726 10.1006/jsbi.1996.0013

[59] Buchholz, T.-O., Jordan, M., Pigino, G. & Jug, F. Cryo-CARE: content-aware image restoration for cryo-transmission electron microscopy data. In *2019 IEEE 16th International Symposium on Biomedical Imaging (ISBI 2019)*, 502–506 (IEEE, 2019).

[60] Schindelin, J. et al. Fiji: an open-source platform for biological-image analysis. *Nat. Methods* **9**, 676–682 (2012). 22743772 10.1038/nmeth.2019 PMC3855844

