# Supplementary material for: Structural basis of lenacapavir-induced HIV-1 capsid defects during virion maturation
Source: Nat Commun. 2026 Sep 22;17:9830. doi: 10.1038/s41467-026-77803-7 (PMC13598136; doi:10.1038/s41467-026-77803-7)
Supplement: Supplementary file 1 [file 41467_2026_77803_MOESM11_ESM.pdf]

nature portfolio

Corresponding author(s): Shinichi Machida

Last updated by author(s): Aug 28, 2026

Reporting Summary

Nature Portfolio wishes to improve the reproducibility of the work that we publish. This form provides structure for consistency and transparency in reporting. For further information on Nature Portfolio policies, see our Editorial Policies and the Editorial Policy Checklist.

Statistics

For all statistical analyses, confirm that the following items are present in the figure legend, table legend, main text, or Methods section.

n/a	Confirmed	
☐	☒	The exact sample size (n) for each experimental group/condition, given as a discrete number and unit of measurement
☐	☒	A statement on whether measurements were taken from distinct samples or whether the same sample was measured repeatedly
☐	☒	The statistical test(s) used AND whether they are one- or two-sided<br>*Only common tests should be described solely by name; describe more complex techniques in the Methods section.*
☒	☐	A description of all covariates tested
☒	☐	A description of any assumptions or corrections, such as tests of normality and adjustment for multiple comparisons
☐	☒	A full description of the statistical parameters including central tendency (e.g. means) or other basic estimates (e.g. regression coefficient) AND variation (e.g. standard deviation) or associated estimates of uncertainty (e.g. confidence intervals)
☒	☐	For null hypothesis testing, the test statistic (e.g. F, t, r) with confidence intervals, effect sizes, degrees of freedom and P value noted<br>*Give P values as exact values whenever suitable.*
☒	☐	For Bayesian analysis, information on the choice of priors and Markov chain Monte Carlo settings
☒	☐	For hierarchical and complex designs, identification of the appropriate level for tests and full reporting of outcomes
☒	☐	Estimates of effect sizes (e.g. Cohen's d, Pearson's r), indicating how they were calculated

Our web collection on statistics for biologists contains articles on many of the points above.

Software and code

Policy information about availability of computer code

Data collection	EPU, Tomography 5
Data analysis	cryoSPARC (v4.4.1 and v5.0.2), Chimera (v1.16), ChimeraX (v1.10), Phenix (v1.21.2-5419), Coot (v0.9.5), AreTomo2 (v1.0), AreTomo3 (v2.2.2), IsoNet (v0.2), IMOD (v4.11.25 and v5.1.9), R (v4.5.0), PyMOL (v3.1.0), cryoCARE (v0.3.0), Fiji (v2.16.0)

For manuscripts utilizing custom algorithms or software that are central to the research but not yet described in published literature, software must be made available to editors and reviewers. We strongly encourage code deposition in a community repository (e.g. GitHub). See the Nature Portfolio guidelines for submitting code & software for further information.

Data

Policy information about availability of data

All manuscripts must include a data availability statement. This statement should provide the following information, where applicable:

- Accession codes, unique identifiers, or web links for publicly available datasets
- A description of any restrictions on data availability
- For clinical datasets or third party data, please ensure that the statement adheres to our policy

Cryo-EM maps have been deposited in the Electron Microscopy Data Bank (EMDB) under the accession numbers EMD-81945 (LEN-bound HIV-1 capsid lattice within VLPs treated with PFO, C6 symmetry), EMD-81946 (LEN-bound HIV-1 capsid lattice within VLPs treated with PFO, no symmetry), EMD-81947 (LEN-unbound HIV-1 capsid lattice within VLPs treated with PFO, C6 symmetry), EMD-81948 (LEN-unbound HIV-1 capsid lattice within VLPs treated with PFO, no symmetry) and

EMD-67116 (LEN-bound HIV-1 capsid lattice within intact VLPs). The coordinates have been deposited in the Protein Data Bank (PDB) under accession numbers 43KN (LEN-bound HIV-1 capsid lattice within VLPs treated with PFO), 43KO (LEN-unbound HIV-1 capsid lattice within VLPs treated with PFO), and 9XQH (LEN-bound HIV-1 capsid lattice within intact VLPs).

Research involving human participants, their data, or biological material

Policy information about studies with human participants or human data. See also policy information about sex, gender (identity/presentation), and sexual orientation and race, ethnicity and racism.

Reporting on sex and gender	not applicable
Reporting on race, ethnicity, or other socially relevant groupings	not applicable
Population characteristics	not applicable
Recruitment	not applicable
Ethics oversight	not applicable

Note that full information on the approval of the study protocol must also be provided in the manuscript.

Field-specific reporting

Please select the one below that is the best fit for your research. If you are not sure, read the appropriate sections before making your selection.

☒ Life sciences ☐ Behavioural & social sciences ☐ Ecological, evolutionary & environmental sciences

For a reference copy of the document with all sections, see nature.com/documents/nr-reporting-summary-flat.pdf

Life sciences study design

All studies must disclose on these points even when the disclosure is negative.

Sample size	No statistical methods were used to predetermine sample size. The effects described here are obvious, reproducible, clear, and homogeneousenough that no statistical methods were required to extract the trait.
Data exclusions	None.
Replication	Described in the relevant figure legend and the replicated experimental data are shown in Supplementary Figure and Source Data File2.
Randomization	Randomization was not applied as experiments used homogeneous cell populations and predefined in vitro conditions.
Blinding	Investigators were not blinded to allocation during the experiments and analysis

Reporting for specific materials, systems and methods

We require information from authors about some types of materials, experimental systems and methods used in many studies. Here, indicate whether each material, system or method listed is relevant to your study. If you are not sure if a list item applies to your research, read the appropriate section before selecting a response.

Materials & experimental systems

n/a	Involved in the study	
☐	☒	Antibodies
☐	☒	Eukaryotic cell lines
☒	☐	Palaeontology and archaeology
☒	☐	Animals and other organisms
☒	☐	Clinical data
☒	☐	Dual use research of concern
☒	☐	Plants

Methods

n/a	Involved in the study	
☒	☐	ChIP-seq
☐	☒	Flow cytometry
☒	☐	MRI-based neuroimaging

Antibodies

Antibodies used	anti-CA (NIH AIDS Reagent Program, Cat# 4250, 1:1000 dilution), anti-IN (Santa Cruz, sc-69721, 1:500 dilution) and anti-RT (Abcam, ab63911, 1:1000 dilution)
Validation	not applicable

Eukaryotic cell lines

Policy information about cell lines and Sex and Gender in Research

Cell line source(s)	HEK293T Lenti-X (Takara/Clontech, 632180), Jurkat (ATCC, TIB-152)
Authentication	Commercially available
Mycoplasma contamination	negative
Commonly misidentified lines (See ICLAC register)	no commonly misidentified cell line was used in the study,

Plants

Seed stocks	not applicable
Novel plant genotypes	not applicable
Authentication	not applicable

Flow Cytometry

Plots

Confirm that:

☐ The axis labels state the marker and fluorochrome used (e.g. CD4-FITC).

☐ The axis scales are clearly visible. Include numbers along axes only for bottom left plot of group (a 'group' is an analysis of identical markers).

☐ All plots are contour plots with outliers or pseudocolor plots.

☒ A numerical value for number of cells or percentage (with statistics) is provided.

Methodology

Sample preparation	Jurkat cells, confirmed to be mycoplasma-free, were infected with VSV-G-pseudotyped HIV-1-GFP produced in the presence or absence of LEN. The multiplicity of infection (MOI) was set to 0.3, as determined by the percentage of GFP-positive Jurkat cells infected with VSV-G-pseudotyped HIV-1-GFP produced in the absence of LEN. For infections with viruses produced in the presence of LEN, equivalent amounts of viral RNA to that of LEN-free virus were used. After 3 hours, the cells were washed twice with PBS, and the culture medium was replaced. At 48 hours postinfection (hpi), the cells were collected and fixed with 4% paraformaldehyde and subjected to flow cytometry analysis using a BD FACSVerse (BD Biosciences).
Instrument	BD FACSVerse (BD Biosciences)
Software	BD FACSuite v1.0.5.3841 (BD Biosciences)
Cell population abundance	The proportion of GFP-positive cells was used to quantify the infected cell population. Under LEN-free conditions at MOI 0.3, approximately 30% of Jurkat cells were GFP^{+} , and this value was used to normalize input virus amounts. For viruses produced in the presence of LEN, equivalent viral RNA levels were applied, and the percentage of GFP^{+} cells at 48 hpi was determined for comparison across conditions.

Gating strategy	Cells were first gated on FSC–SSC to exclude debris, followed by singlet discrimination using FSC-H/FSC-A. GFP fluorescence (FITC channel) was then used to define GFP-positive and GFP-negative populations based on an unstained, uninfected control. Identical gating parameters were applied across all samples.

☒ Tick this box to confirm that a figure exemplifying the gating strategy is provided in the Supplementary Information.
