# Supplementary material for: Structural basis of lenacapavir-induced HIV-1 capsid defects during virion maturation
Source: Nat Commun. 2026 Sep 22;17:9830. doi: 10.1038/s41467-026-77803-7 (PMC13598136; doi:10.1038/s41467-026-77803-7)
Supplement: Supplementary file 2 [file 41467_2026_77803_MOESM12_ESM.pdf]

nature portfolio

Peer Review File

Structural basis of lenacapavir-induced HIV-1 capsid defects during virion maturation

Corresponding Author: Dr Shinichi Machida

This file contains all reviewer reports in order by version, followed by all author rebuttals in order by version.

Version 0:

Reviewer comments:

Reviewer #1

(Remarks to the Author)
Summary
The authors use a combination of cryo-electron microscopy, in vitro experiments with capsids isolated from detergent-treated virions, and cell-based assays to investigate the effect of LEN on the late stage of the HIV-1 life cycle. The study reports that LEN treatment during virus production results in malformed mature capsids that exhibit perturbations in capsid lattice curvature. These structural defects correlate with reduced levels of core-associated reverse transcriptase, decreased reverse transcription and integration, and reduced infectivity. These observations are consistent with previous studies demonstrating that HIV-1 capsids must remain intact to support efficient reverse transcription and productive infection. The authors conclude that LEN binding to the viral core induces a flattened lattice geometry that compromises core integrity and reduces infectivity of virions produced in the presence of LEN.
Critique
Overall, this is a well-designed study to determine the effects of LEN treatment during the late stage of viral replication, which includes virion assembly and budding, on viral core structure in intact virions and isolated cores. The conclusions drawn are largely supported by the data presented and the manuscript is well-written. There are some potential issues that could be potentially resolved quickly and would strengthen the manuscript.
Throughout the manuscript, the authors imply that LEN binds to viral cores, which induces a flattened lattice geometry, resulting in compromised core integrity and reduced infectivity. This interpretation, which is also implied in the title of manuscript, implies that LEN acts primarily after the capsid has formed. An alternative interpretation is that the observed defects arise during capsid assembly. In particular, the reduced formation of pentamers in the presence of LEN may represent the primary cause of the malformed capsids (PMID: 39869652). The authors should more explicitly discuss the potential effects of LEN both before and after formation of the mature capsid during virion maturation and clarify whether their data distinguish between these possibilities. The phrase “capsid disruption” implies that the capsids were formed and then disrupted or broken, and doesn’t acknowledge the possibility that the capsids were malformed as a result of LEN binding to the immature capsid lattice. I suggest a change in the title to something like “Structural basis for lenacapavir-induced HIV-1 capsid defects during virion maturation”.
The manuscript has several Extended Data Figures. It would be helpful to the reader to place some of these data into the main figures, particularly Extended Data Figures 1, 4, 7, and 8.
Minor concerns
Fig. 1: The authors should explicitly state that 50 nM LEN was used for the initial virion-production experiments. The authors should discuss how 50 nM compares to the EC50 for the late-stage effect.
a–c: Is it possible to quantify capsid area in addition to the measurements shown?
f,g: What is the occupancy of LEN at 50 nM? Would similar effects be observed for lower occupancy?
Why was a packaging plasmid psPAX2 used for some experiments? This plasmid does not produce a packageable viral genome.
Fig. 2 b,c: Are the structural rearrangements observed at the CTD–CTD dimer interface and the trimer interface correlated with LEN binding at the adjacent FG pocket? Alternatively, are these allosteric effects arising from LEN binding to more distant FG pockets?
Fig. 3: The difference between peak fractions of control and LEN-treated cores in the sucrose gradient appears modest, and it is not clear whether this difference is reproducible. Based on this difference, the authors suggest that the cores produced in the presence of LEN have structural alterations. The authors show either provide evidence that this difference in migration is reproducible in several independent sucrose gradients or remove this interpretation of the result.

There appears to be more p24 present in the gradient fractions for the LEN-treated cores, consistent with LEN's ability to stabilize the capsid lattice. This could confound the interpretation of the sucrose gradient experiments, particularly when quantifying RT and IN levels (see comment below).
a,b: The authors state that IN and viral RNA are retained within cores produced in the presence of LEN at levels similar to those in control cores. However, this conclusion is not entirely convincing because IN and RNA levels appear similar despite the higher amount of p24 detected in the corresponding fractions. The authors should quantify the p24 levels. These results may indicate that, after normalizing for p24, some of the IN separates from disrupted capsids during the assay.
g: Does sucrose affect RT activity? There is higher RT activity in the initial fractions than in the core fractions, suggesting that many capsids in both the LEN− and LEN+ conditions may be disrupted shortly after entering the sucrose gradient. Alternatively, RT activity in the core fractions may be severely underestimated, particularly if sucrose inhibits RT activity.
Panel g is cited before panels d–f.
Fig. 4: Although the reduced reverse transcription, integration, and infectivity observed after infection with LEN-produced virions are consistent with capsid defects present prior to infection, can the authors rule out carryover of LEN into the target cells, particularly given LEN's high affinity for the capsid?
Is it surprising that the infectivity of the M66I mutant is only 3-fold lower than WT?
Results section "Structural perturbations impair HIV-1 infectivity": The phrasing for one of the sentences is awkward. Instead of "…the infectivity of HIV-GFP produced in the presence of LEN was reduced to 0.003% in GFP-positive cells," it would be clearer to state: "the percentage of GFP-positive cells was reduced to 0.003%."

Reviewer #2

(Remarks to the Author)
This work by Tanaka et al. proposes a structural understanding for the previously reported observation of lenacapavir disruption of the HIV-1 capsid despite hyperstabilisation of the CA protein hexamer. The authors extend discussion of loss of capsid integrity with functional data correlating presence of LEN with integrase, reverse transcriptase, and vRNA and assessing viral replication and DNA synthesis ± LEN. This is an interesting and useful contribution. Some suggestions for consideration by the authors to improve the discussion of structural changes upon lenacapavir addition are listed below.

Major comments:

• How were interhexamer tilt angles calculated (what three points)? The figures seem to suggest this calculation was done based on models. Are there statistics for the model fit for the adjacent (less well resolved) hexamers?
• The reconstructions (both with and without PFO) appear to exhibit anisotropy (Extended Data Figs 2 and 5) in the axis which could skew interhexamer angle measurements. Whether there is diverse enough orientation data to support the argument of altered interhexamer angles will need to be addressed.
• Can the authors justify why they chose to use previously published structures instead of reconstructing a CA lattice structure in-house from VLPs generated without LEN to rule out differences due to methodology?
• Fig 2b,c: Please provide RMSD values for the discussed helices to support the effect of LEN on the respective contacts. If possible, highlight some key contacts observed to be disrupted with addition of LEN. Comment on observed changes resulting in the flattened angle.

Minor comments:

• What are the median VLP areas in the +LEN and -LEN conditions? Perhaps providing mean values would also help support this difference.
• If possible, it would be interesting to speculate a rationale for why the majority of VLPs in the +LEN condition still cluster at the same size as -LEN, but there is a class that seemingly balloons dramatically in size
• What is the frequency of aberrant capsid structure in the +LEN condition? What is the frequency of each type of aberrant structure? It would help reader understanding to produce a chart comparing these distributions ±LEN.
• Please comment on to what extent the 2D and 3D (cryo-ET) data agree with each other
• Page 5, lines 13-14 ("revealed a sixfold symmetric structure") and Extended Data Figure 3: Was a parallel reconstruction produced from the same dataset without symmetry imposed?
• Page 2, line 12: The biochemical data support loss of reverse transcriptase activity, not strictly dissociation of the protein, although this is the most likely cause. The abstract need not be edited, but this distinction should be pointed out in the main text discussion.
• Consider including an annotated schematic of what primers for HIV DNA quantitation are targeting as in https://doi.org/10.1126/science.abc8420 Fig. 1 and refer to the extended data figure at the first mention of these targets.
• Please provide methodological detail for flow cytometry experiments: laser lines and filters
• Page 5, line 1: Citation 6 discusses GS-CA1, not GS-6207
• Page 2, sentence 2 (lines 3-7) is a little wordy and difficult to understand on first reading
• Page 4, end of paragraph 1: capitalize Gag
• Page 4, para. 2 ("alters the curvature parameters required for proper cone closure"): clarify that the question is whether curvature specifically is responsible. Citation 8 shows LEN stabilization of the CA hexamer lattice despite broken capsids not forming cones.
• It would be helpful to the reader to include an indicator of where the LEN binding site is on the structure overview e.g. Fig. 2a
• Include labels on the figures for each subpanel so that readers have an easier time understanding the data without needing to read the details in the legend

• Page 9, line 13 (“early reverse transcription (ERT)”) and subsequent uses of “ERT”: This can be confusing since “ERT” is often used to refer to endogenous reverse transcription. Consider using a different abbreviation.

Reviewer #3

(Remarks to the Author)
The manuscript by Tanaka et al. provides important structural insights into the late-stage effects of lenacapavir on the HIV-1 capsid architecture. The authors utilized complementary cryo-EM, biochemistry and virology assays. The study is generally well structured and follows a logical progression. Structural and functional data are well integrated. However, several key conclusions regarding capsid disruption and the mechanistic role of the lattice flattening require further clarification and additional experimental data. Addressing the points below would significantly strengthen the manuscript.

Major comments
1. Quantification of aberrant capsid structures are needed. While the authors show that LEN induces irregular capsid morphologies, the observed morphological defects (e.g., percentages of intact vs fractured, enlarged, aberrant, or tubular structures in LEN untreated vs treated VLPs) are not quantitated. Furthermore, it would be important to assess how these phenotypes vary with varying LEN concentrations. The authors claim that partial LEN occupancy is sufficient to compromise capsid integrity which need to be supported by quantitation results.

2. Cryo-ET experiments and inter-hexamer tilt angles require additional work. The authors use their own cryo-ET results with the VLPs prepared in the presence of LEN and compare these findings with the published structures of native capsid from other groups. The preparations of native cores and resolutions of the cryo-EM maps in published studies varies. Therefore, I strongly suggest the authors obtain their own cryo-ET data with untreated VLPs to side-by-side compare their own preparations of VLPs in the absence and presence of LEN. Furthermore, quantitative analysis (mean values with deviations) of the tilt angles between VLPs in the absence and presence of LEN are needed (for example see the recent preprint PMID: 41542592). Moreover, the authors need to detail the methodology for calculating inter-hexamer tilt angles. It should be explained how hexamer orientations were defined, how angles were computed, and which programs were used.

3. Information regarding pentamer-hexamer interfaces is lacking. The structural analysis focuses primarily on inter-hexamer interactions and lattice flattening. However, capsid curvature and closure critically depend on pentamers. Given that LEN binds only hexamers, it would be important to address how LEN affects pentamer–hexamer interfaces. Is the lattice flattening alone sufficient to induce rupture or are high-curvature regions (pentamer-rich) preferentially destabilized?

4. The VLPs produced in the presence of LEN appear structurally heterogeneous. It is therefore unclear whether the reconstructed lattice represents a single dominant conformation or an average over multiple structural states. The authors should address the degree of particle heterogeneity and whether multiple classes of capsid architectures exist. A larger box size of the capsid lattice would give a larger cryo-EM map with better resolved adjacent hexamers that surround the central hexamer. Subsequent heterogeneous refinements of the consensus map could delineate the different classes (different curvatures, etc).

Minor comments
5. Cryo-EM processing workflow need to be described better. The cryo-EM data processing is unclear, particularly regarding the sequence of heterogeneous refinement and 2D classification steps. Please clarify the order of processing steps, and the rationale for the chosen workflow. The author used C6 symmetry in a heterogeneous refinement after the initial 2D classification. Also, no ab-initio reconstruction step with C1? Please provide the reasons for doing so.

6. The structural analyses are performed at 50 nM LEN, which exceeds reported pharmacologically relevant concentrations of the antiretroviral (~5 nM). While the authors justify this choice to ensure high occupancy, it raises a possibility that the observed structural effects reflect near-saturating conditions. Clarify how these findings relate to physiological conditions and, if possible, include supporting structural data for lower LEN concentrations.

7. The results showing the RT activity loss and impaired ERT in the target cells are exciting. I suggest supplementing these results by examining RT levels by immunoblotting analysis of sucrose gradient fractions (similar to CA and IN immunoblots shown in Fig 3,a,b,d,e). If RT antibodies are not sensitive enough, the total protein from the peak fractions could be precipitated to assess RT levels by western blot.

8. Unit/formatting issue. The sentence referring to a “5 nM2” concentration appears to contain a formatting error and should be corrected.

9. I suggest that authors discuss preprint PMID: 41542592. The findings in the preprint and the current manuscript are similar in terms of LEN fracturing the capsid, which in turn results in the RT loss. However, the important distinctions between these two studies are that the current manuscript shows that LEN can induce both the lattice enlargement and fracturing during HIV-1 particle assembly, whereas the preprint demonstrates that LEN can additionally fracture the properly matured cores.

Reviewer #4

(Remarks to the Author)
I co-reviewed this manuscript with one of the reviewers who provided the listed reports. This is part of the Nature

Communications initiative to facilitate training in peer review and to provide appropriate recognition for Early Career Researchers who co-review manuscripts.

Reviewer #5

(Remarks to the Author)
I co-reviewed this manuscript with one of the reviewers who provided the listed reports. This is part of the Nature Communications initiative to facilitate training in peer review and to provide appropriate recognition for Early Career Researchers who co-review manuscripts.

Version 1:

Reviewer comments:

Reviewer #1

(Remarks to the Author)
The authors have addressed all of the concerns and comments that we raised in our review of the original manuscript. As a result, the manuscript is significantly improved and makes a strong contribution to our understanding of how lenacapavir influences HIV-1 capsid structure and infectivity.
In their response to the reviewer comments, the authors have added results of two additional sucrose gradient fractionation experiments, and measured RT activity in the presence of increasing concentrations of sucrose to show that sucrose levels did not influence RT activity. These data should be included as supplemental data in the revised manuscript, so that other readers who may have the same questions will have the data available.
In addition, the authors purified virions through a second sucrose cushion to remove residual free lenacapavir to show that the infectivity of the purified particles was not affected by carryover of free lenacapavir. If possible, the authors should add these data as a supplemental figure.

Reviewer #2

(Remarks to the Author)
Tha authors have satisfactorily addressed all of my comments and concerns.

Reviewer #3

(Remarks to the Author)
Our comments have been addressed adequately.

Reviewer #4

(Remarks to the Author)
I co-reviewed this manuscript with one of the reviewers who provided the listed reports. This is part of the Nature Communications initiative to facilitate training in peer review and to provide appropriate recognition for Early Career Researchers who co-review manuscripts.

Reviewer #5

(Remarks to the Author)
I co-reviewed this manuscript with one of the reviewers who provided the listed reports. This is part of the Nature Communications initiative to facilitate training in peer review and to provide appropriate recognition for Early Career Researchers who co-review manuscripts.

Reviewer #6

(Remarks to the Author)
I co-reviewed this manuscript with one of the reviewers who provided the listed reports. This is part of the Nature Communications initiative to facilitate training in peer review and to provide appropriate recognition for Early Career Researchers who co-review manuscripts.

Open Access This Peer Review File is licensed under a Creative Commons Attribution 4.0 International License, which permits use, sharing, adaptation, distribution and reproduction in any medium or format, as long as you give appropriate credit to the original author(s) and the source, provide a link to the Creative Commons license, and indicate if changes were made.
In cases where reviewers are anonymous, credit should be given to 'Anonymous Referee' and the source.
The images or other third party material in this Peer Review File are included in the article's Creative Commons license, unless indicated otherwise in a credit line to the material. If material is not included in the article's Creative Commons license and your intended use is not permitted by statutory regulation or exceeds the permitted use, you will need to obtain permission directly from the copyright holder.
To view a copy of this license, visit https://creativecommons.org/licenses/by/4.0/

Point-by-point responses

Reviewer #1 (Remarks to the Author):

Summary

The authors use a combination of cryo-electron microscopy, in vitro experiments with capsids isolated from detergent-treated virions, and cell-based assays to investigate the effect of LEN on the late stage of the HIV-1 life cycle. The study reports that LEN treatment during virus production results in malformed mature capsids that exhibit perturbations in capsid lattice curvature. These structural defects correlate with reduced levels of core-associated reverse transcriptase, decreased reverse transcription and integration, and reduced infectivity. These observations are consistent with previous studies demonstrating that HIV-1 capsids must remain intact to support efficient reverse transcription and productive infection. The authors conclude that LEN binding to the viral core induces a flattened lattice geometry that compromises core integrity and reduces infectivity of virions produced in the presence of LEN.

Critique

Overall, this is a well-designed study to determine the effects of LEN treatment during the late stage of viral replication, which includes virion assembly and budding, on viral core structure in intact virions and isolated cores. The conclusions drawn are largely supported by the data presented and the manuscript is well-written. There are some potential issues that could be potentially resolved quickly and would strengthen the manuscript.

Our response: We thank the reviewer for the careful evaluation of our manuscript and for the positive assessment of this study. We also appreciate the constructive and helpful comments, which allowed us to improve the clarity and strength of the manuscript. We believe that the revisions have improved the clarity and strength of the manuscript.

Throughout the manuscript, the authors imply that LEN binds to viral cores, which induces a flattened lattice geometry, resulting in compromised core integrity and reduced infectivity. This interpretation, which is also implied in the title of manuscript, implies that LEN acts primarily after the capsid has formed. An alternative interpretation is that the observed defects arise during capsid assembly. In particular, the reduced formation of pentamers in the presence of LEN may represent the primary cause of the malformed capsids (PMID: 39869652). The authors should more explicitly discuss the potential effects of LEN both before and after formation of the mature capsid during virion maturation and clarify whether their data distinguish between these possibilities. The phrase “capsid disruption” implies

that the capsids were formed and then disrupted or broken, and doesn't acknowledge the possibility that the capsids were malformed as a result of LEN binding to the immature capsid lattice. I suggest a change in the title to something like "Structural basis for lenacapavir-induced HIV-1 capsid defects during virion maturation".

Our response: We agree with the reviewer's important comment. We have therefore changed the title to "Structural basis of lenacapavir-induced HIV-1 capsid defects during virion maturation," as suggested. In addition, we revised the Discussion to explicitly state the following: "Because LEN was present throughout virus production in our experiments, our data do not completely distinguish whether the observed capsid defects arose during capsid assembly, during cone closure, or immediately after the formation of the mature capsid. Therefore, LEN-induced lattice flattening may impair both capsid closure and the subsequent maintenance of core integrity." (lines 343–348).

The manuscript has several Extended Data Figures. It would be helpful to the reader to place some of these data into the main figures, particularly Extended Data Figures 1, 4, 7, and 8.

Our response: We thank the reviewer for this helpful suggestion. We have reorganized the figures and moved the key data from Extended Data Figs. 1, 4, 7, and 8 into the main figures (new Figs. 1d, 2b–g, and 5f) to improve readability and better support the flow of the manuscript.

Minor concerns

Fig. 1: The authors should explicitly state that 50 nM LEN was used for the initial virion-production experiments. The authors should discuss how 50 nM compares to the EC50 for the late-stage effect.

Our response: We thank the reviewer for this suggestion. We now state the following: "For this structural analysis, we used VLPs produced in the presence of 50 nM LEN to ensure near-complete lattice occupancy" (Results section: lines 98–100). In addition, we added "Cryo-EM images of the VLPs produced in the absence (a) or presence of 50 nM LEN (b, c)" (Fig. 1 legend: lines 760–761).

We also added a new concentration-dependent cryo-ET analysis showing that capsid defects are already observed at 2–5 nM LEN. Since the pharmacologically relevant concentration of LEN has been reported to be approximately 5 nM, these data indicate that LEN-induced capsid morphological defects occur within a low-nanomolar concentration range (new Fig. 6d–m, Results section: lines 223–253).

a–c: Is it possible to quantify capsid area in addition to the measurements shown?
Our response: We thank the reviewer for this helpful suggestion. We considered quantifying capsid area; however, under the LEN+ condition, capsid structures were highly heterogeneous, including incompletely closed, tubular, sheet-like, and fragmented capsids. Because these structures often lacked clearly defined capsid boundaries, reliable and consistent area measurements were not feasible.
Instead, we quantified the capsid morphology by classifying the particles into mature, immature, and atypical categories and added these data to the revised manuscript (Fig. 6i, j; Results section: lines 226–236). This analysis more appropriately captures the heterogeneous capsid defects induced by LEN.

f,g: What is the occupancy of LEN at 50 nM? Would similar effects be observed for lower occupancy?
Our response: We thank the reviewer for these important questions. Although the exact LEN occupancy cannot be quantitatively determined from the current cryo-EM map, the clear density at the FG binding pocket indicates substantial occupancy after treatment with 50 nM LEN. We used this concentration to obtain a sufficiently homogeneous LEN-bound CA lattice for the SPA-based structural determination.
We performed a concentration-dependent cryo-ET analysis to assess whether similar effects occur at lower LEN concentrations (new Fig. 6d–m, Results section: lines 223–253). This analysis showed that capsid morphological defects are already observed after treatment with 2–5 nM LEN, indicating that complete occupancy is not required to perturb capsid maturation.

Why was a packaging plasmid psPAX2 used for some experiments? This plasmid does not produce a packageable viral genome.
Our response: We thank the reviewer for noting this issue. psPAX2 was used only to produce Gag-Pol VLPs for structural analyses, where a packageable viral genome was not needed. We chose this system because it allowed us to analyse native capsid structures without additional biosafety barriers or chemical fixation, such as PFA treatment. For infectivity and viral DNA synthesis assays (Fig. 7), we used VSV-G-pseudotyped HIV-1 particles generated from pNL4-3.GFP E- and pMD2.G. In addition, we performed the cryo-ET analysis using NL4-3E- virions treated with PFA and observed LEN-dependent capsid morphological defects (new Fig. 6d–m, Results section: lines 223–253), confirming that the phenotype is not specific to psPAX2-derived VLPs.

Fig. 2 b,c: Are the structural rearrangements observed at the CTD–CTD dimer interface and the trimer interface correlated with LEN binding at the adjacent FG pocket? Alternatively, are these allosteric effects arising from LEN binding to more distant FG pockets?

Our response: We thank the reviewer for these important questions. As suggested by Reviewers 2 and 3, we determined an in-house LEN-unbound CA lattice structure from PFO-treated VLPs acquired under the same imaging conditions as the LEN-bound dataset (Supplementary Figs. 3 and 4). We then directly compared the LEN-unbound and LEN-bound lattices under matched structural conditions (Fig. 2a).

This side-by-side comparison showed conformational changes around the FG pocket, which is the LEN binding site (Fig. 4a, b). Compared with the LEN-unbound CA hexamer, the LEN-bound CA hexamer showed closer positioning of NTD helix 4 and CTD helix 9 from neighbouring CA subunits within the same hexamer, indicating a local rearrangement of the intrahexamer NTD–CTD interface (Fig. 4b). This interpretation is consistent with previous structural work showing that LEN binds across two adjoining CA subunits and directly contacts both the NTD and the CTD, including the CTD helix 9 region (Bester *et al*. *Science*, 2020, PMCID: PMC7831379).

Based on these observations, we consider that LEN binding at the adjacent FG pocket likely locally alters CTD positioning, which contributes to the rearrangement of the CTD-mediated interhexamer trimer interface formed primarily by helix 10. However, because LEN binds repeatedly throughout the hexameric lattice, our current structures do not allow us to exclude additional allosteric contributions from more distant LEN-bound FG pockets. We have revised the relevant text (Results section: lines 147–164) and added a new Fig. 4.

Fig. 3: The difference between peak fractions of control and LEN-treated cores in the sucrose gradient appears modest, and it is not clear whether this difference is reproducible. Based on this difference, the authors suggest that the cores produced in the presence of LEN have structural alterations. The authors show either provide evidence that this difference in migration is reproducible in several independent sucrose gradients or remove this interpretation of the result.

Our response: We thank the reviewer for this important comment. We have added the results from two additional independent experiments using sucrose gradient fractionation below, resulting in a total of three independent experiments. These experiments showed a

similar trend, with LEN-treated cores migrating slightly towards heavier fractions than control cores did.

However, we agree that the difference in peak migration is modest. We therefore removed the statement that “the shift to a heavier fraction for the cores produced in the presence of LEN suggests structural alterations, which is consistent with the cryo-EM images of the VLPs”, and revised the text to avoid overinterpreting this result.

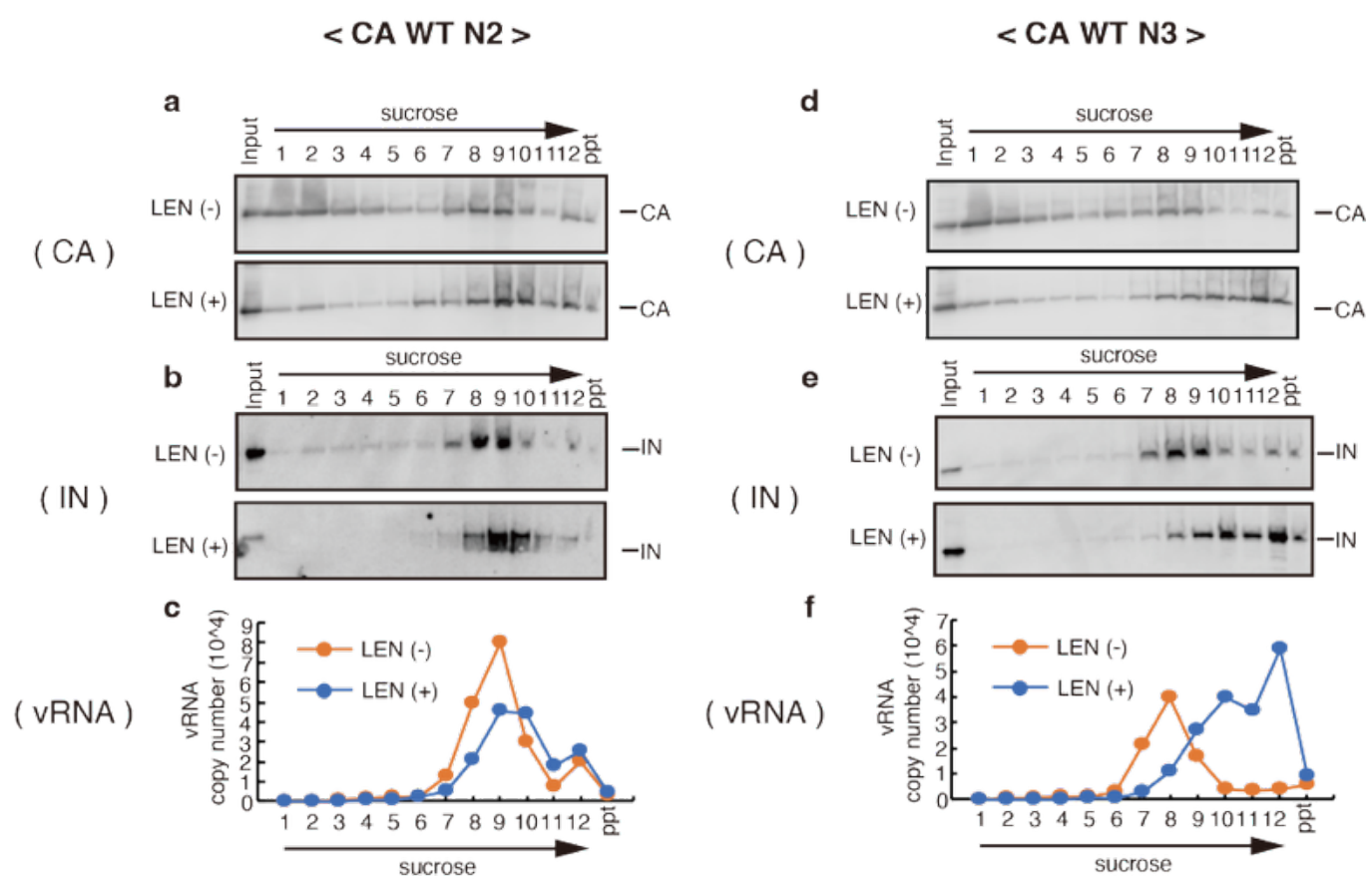

Sucrose gradient fractionation of WT HIV-GFP virions produced in the presence or absence of LEN. CA (a, d) and IN (b, e) levels were detected by western blotting. The amount of viral RNA in each fraction was quantified by RT–qPCR (c, f). The first experiment is shown as representative data in Fig. 5a–c, and the results of the two additional independent experiments are provided.

There appears to be more p24 present in the gradient fractions for the LEN-treated cores, consistent with LEN’s ability to stabilize the capsid lattice. This could confound the interpretation of the sucrose gradient experiments, particularly when quantifying RT and IN levels (see comment below).

a,b: The authors state that IN and viral RNA are retained within cores produced in the presence of LEN at levels similar to those in control cores. However, this conclusion is not entirely convincing because IN and RNA levels appear similar despite the higher amount of p24 detected in the corresponding fractions. The authors should quantify the p24 levels. These results may indicate that, after normalizing for p24, some of the IN separates from disrupted capsids during the assay.

Our response: We thank the reviewer for this important comment. We agree that differences in p24 levels could affect the interpretation of IN and RT retention in the sucrose gradient fractions.

To address this concern, we used the LEN– and LEN+ peak fractions and adjusted the input so that equivalent amounts of p24 were loaded. We then detected RT and IN levels by western blotting. Under these p24-normalized conditions, IN remained detectable in the LEN+ peak fraction, whereas the level of RT was markedly reduced. These results support

our conclusion that LEN-containing cores preferentially lose RT while retaining substantial amounts of IN.
We have added these data (new Fig. 5e) to the revised manuscript.

g: Does sucrose affect RT activity? There is higher RT activity in the initial fractions than in the core fractions, suggesting that many capsids in both the LEN– and LEN+ conditions may be disrupted shortly after entering the sucrose gradient. Alternatively, RT activity in the core fractions may be severely underestimated, particularly if sucrose inhibits RT activity.
Our response: We thank the reviewer for raising this important concern. We tested whether sucrose affects the SG-PERT assay using fraction 2 from the LEN– sucrose gradient, diluted it 10-fold with buffers containing 30%, 50%, or 70% sucrose, and measured RT activity (see the figure attached below). RT activity was not substantially reduced under these conditions, indicating that the low RT activity detected in the core-containing fractions was not due to sucrose-dependent inhibition of the assay.

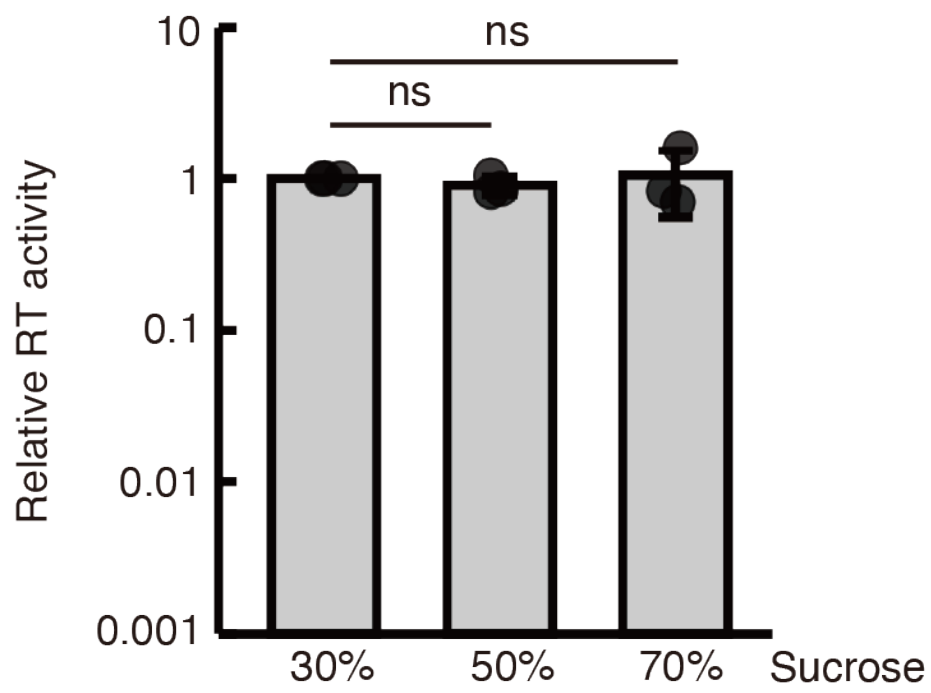

Reverse transcriptase (RT) activity was measured in the presence of 30%, 50%, and 70% sucrose. RT activity was normalized to that of the 30% sucrose sample. The data are shown as the means ± s.d. (n = 3 independent experiments).

Panel g is cited before panels d–f.
Our response: We thank the reviewer for noting this issue. We have reorganized the new Fig. 5; in the revised figure, the WT and M66I results are presented separately and in a more logical order so that each panel is cited sequentially in the text (new Figure 5d, j).

Fig. 4: Although the reduced reverse transcription, integration, and infectivity observed after infection with LEN-produced virions are consistent with capsid defects present prior to infection, can the authors rule out carryover of LEN into the target cells, particularly given LEN's high affinity for the capsid?
Our response: We thank the reviewer for raising this important concern. In the experiments presented in the original manuscript, LEN-produced virions were pelleted through a 20% sucrose cushion before infection to minimize the possibility of LEN carryover. In the revised

study, we further performed a second wash through a sucrose cushion to more rigorously remove any residual free LEN (Supplementary Fig. 8). Even after this additional wash, LEN-produced virions showed almost no infectivity.

These results indicate that the reduced infectivity is not explained by the carryover of free LEN into target cells. We have added the results of this washing experiment to the revised manuscript (Supplementary Fig. 8, Results section: lines 265–269).

Is it surprising that the infectivity of the M66I mutant is only 3-fold lower than WT?

Our response: We thank the reviewer for raising this point. In our experiment, the infectivity of the M66I mutant was reduced compared with that of the WT virus, with the percentage of GFP-positive cells decreasing from 31.6% for WT to 7.86% for M66I, corresponding to 24.8% of the infectivity of the WT virus (Fig. 7e). This finding is broadly consistent with a previous report (Briganti *et al*. *MBio*, 2025, https://doi.org/10.1128/mbio.03613-24; Fig. 6E), in which M66I infectivity was approximately 10–20% that of the WT virus.

The modest difference may be due to differences in the assay systems, including the target cells used, namely, Jurkat cells in our study and MOLT-4 cells in the study by Briganti *et al*., as well as the readout, GFP-positive cells in our study and luciferase activity in the study by Briganti *et al*. In addition, Briganti *et al*. showed that the infectivity of the M66I virus was strongly impaired when cell division was blocked by aphidicolin, suggesting that M66I has a defect in nuclear import through the nuclear pore complex and is therefore more sensitive to the growth state of the target cells.

Importantly, despite its reduced baseline infectivity, M66I retained resistance to LEN-induced defects in infectivity, reverse transcription, and integration. Thus, M66I serves as a specificity control, suggesting that the observed LEN-dependent phenotype is mediated through the CA FG binding pocket.

Results section “Structural perturbations impair HIV-1 infectivity”: The phrasing for one of the sentences is awkward. Instead of “…the infectivity of HIV-GFP produced in the presence of LEN was reduced to 0.003% in GFP-positive cells,” it would be clearer to state: “the percentage of GFP-positive cells was reduced to 0.003%.”

Our response: We thank the reviewer for this helpful suggestion. We have revised the text to clarify this point. (Results section: lines 262–265)

Reviewer #2 (Remarks to the Author):

This work by Tanaka et al. proposes a structural understanding for the previously reported observation of lenacapavir disruption of the HIV-1 capsid despite hyperstabilisation of the CA protein hexamer. The authors extend discussion of loss of capsid integrity with functional data correlating presence of LEN with integrase, reverse transcriptase, and vRNA and assessing viral replication and DNA synthesis ± LEN. This is an interesting and useful contribution. Some suggestions for consideration by the authors to improve the discussion of structural changes upon lenacapavir addition are listed below.

Our response: We thank the reviewer for the positive assessment of our work and for the constructive suggestions. We have revised the manuscript accordingly, as described in our point-by-point responses below.

Major comments:

• How were interhexamer tilt angles calculated (what three points)? The figures seem to suggest this calculation was done based on models. Are there statistics for the model fit for the adjacent (less well resolved) hexamers?
Our response: We thank the reviewer for this helpful comment. We have revised the Methods to clarify that interhexamer tilt angles were calculated using the approach described by Briggs and colleagues (doi.org/10.1073/pnas.2220557120) (Methods: lines 530–536). Briefly, atomic models of adjacent CA hexamers were fitted into the cryo-EM density maps, and a plane was defined for each hexamer using the plane function in UCSF Chimera. The interhexamer tilt angle was then calculated as the angle between the planes of neighbouring hexamers.
In addition, we evaluated the quality of model fit for the adjacent hexamers (Supplementary Figs. 1f and 3f).

• The reconstructions (both with and without PFO) appear to exhibit anisotropy (Extended Data Figs 2 and 5) in the axis which could skew interhexamer angle measurements. Whether there is diverse enough orientation data to support the argument of altered interhexamer angles will need to be addressed.
Our response: We addressed this concern by determining a LEN-unbound CA lattice structure from PFO-treated VLPs in house, with images acquired under the same conditions as those used for the LEN-bound dataset (Supplementary Figs. 3 and 4). We

then directly compared the LEN-unbound and LEN-bound lattices using the same reconstruction and angle measurement procedures. This side-by-side comparison showed that LEN-bound lattices exhibited a reduced interhexamer tilt compared with the LEN-unbound control lattices (Fig. 2a).
Because both datasets were prepared, imaged, processed, and analysed under the same conditions, this comparison reduces the possibility that the observed difference in interhexamer tilt is explained solely by anisotropic reconstruction artefacts or differences in orientation sampling.

• Can the authors justify why they chose to use previously published structures instead of reconstructing a CA lattice structure in-house from VLPs generated without LEN to rule out differences due to methodology?
Our response: We thank the reviewer for this important suggestion. To enable a direct comparison under matched experimental and processing conditions, we newly determined both LEN-unbound and LEN-bound CA lattice structures from VLPs prepared under the same conditions (Supplementary Figs. 1-4). A direct comparison between this LEN-unbound structure and the LEN-bound structure confirmed that LEN reduces the interhexamer tilt and induces a flatter lattice geometry. We have added this comparison with the in-house structure to the revised manuscript (Figs. 2a, 3, and 4).

• Fig 2b,c: Please provide RMSD values for the discussed helices to support the effect of LEN on the respective contacts. If possible, highlight some key contacts observed to be disrupted with addition of LEN. Comment on observed changes resulting in the flattened angle.
Our response: We thank the reviewer for this helpful suggestion. We calculated the RMSD values for the helices discussed in the manuscript and added these data as a new supplementary figure (Supplementary Fig. 7). The RMSD analysis supports a local rearrangement of the intrahexamer NTD–CTD interface and structural changes at the interhexamer trimer interface.
We agree that identifying specific disrupted contacts would be informative. However, although the helical densities at the CTD-mediated trimer interface are sufficiently resolved to compare helix-level rearrangements, the side-chain densities in this region are not sufficiently clear to assign changes in specific amino-acid contacts with confidence. We therefore avoided discussing residue-level disrupted contacts and instead focused on helix-level rearrangements.

We have also revised the text to comment more explicitly on how the observed structural changes may contribute to the flattened interhexamer angle. As suggested by Reviewers 2 and 3, we determined an in-house LEN-unbound CA lattice structure under the same experimental conditions as the LEN-bound structure and directly compared the two lattices. This matched comparison revealed a local rearrangement of the intrahexamer NTD–CTD interface (Fig. 4b, Supplementary Fig. 7a) and structural rearrangement at the CTD-mediated interhexamer trimer interface (Fig. 4d, Supplementary Fig. 7c). We therefore propose that LEN binding locally alters CTD positioning around the FG pocket, which contributes to a rearrangement of the trimer interface and reduces the relative tilt between neighbouring hexamers, resulting in a flatter lattice geometry. We have revised the relevant text (Results section: lines 145–164) and added a new Fig. 4 and Supplementary Fig. 7.

Minor comments:

• What are the median VLP areas in the +LEN and -LEN conditions? Perhaps providing mean values would also help support this difference.
Our response: We thank the reviewer for this helpful suggestion. We have now added both median and mean particle area values for psPAX2-derived VLPs and NL4-3E– virions produced in the absence or presence of LEN (Supplementary Table 1 and Supplementary Table 3).

• If possible, it would be interesting to speculate a rationale for why the majority of VLPs in the +LEN condition still cluster at the same size as -LEN, but there is a class that seemingly balloons dramatically in size
Our response: We thank the reviewer for this helpful suggestion. We have added a brief discussion of this point in the revised manuscript (Results section: lines 337–343).

• What is the frequency of aberrant capsid structure in the +LEN condition? What is the frequency of each type of aberrant structure? It would help reader understanding to produce a chart comparing these distributions ±LEN.
Our response: We thank the reviewer for this helpful suggestion. We have now quantified capsid morphologies in the cryo-ET datasets using NL4-3E– virions and added a chart comparing the distributions among LEN-free and 2, 5, 10, and 50 nM LEN conditions (Fig. 6).
In the revised analysis, the particles were classified into mature, immature, and atypical capsid morphologies. The atypical category included particles with multiple cores, tubular

cores, rod-shaped cores, sheet-like cores, or other aberrant capsid structures. Because these aberrant morphologies were not always clearly distinguishable from one another in the tomograms, we grouped them as atypical morphologies rather than subdividing them further. This quantification showed that LEN treatment markedly increased the frequency of capsids with atypical structures, with the number of abnormal capsid morphologies already increasing in the presence of 2–5 nM LEN. In addition, LEN can compromise capsid integrity even when the overall mature cone-shaped morphology is retained, as visible gaps are frequently observed in cone-shaped capsids. We have added these quantitative data to the revised manuscript (Fig. 6d–m) and revised the Results accordingly (lines 223–253).

• Please comment on to what extent the 2D and 3D (cryo-ET) data agree with each other
Our response: The 2D cryo-EM images (Figure 1a–d) showed that VLPs produced in the presence of LEN frequently contain irregular capsid morphologies and display a broader particle size distribution. The cryo-ET data (Figure 6a–c and Supplementary Movies 1–3) further supported these observations in three dimensions, directly revealing atypical capsid architectures, including incompletely closed, tubular, and sheet-like structures. Thus, the results of the 2D and 3D analyses are consistent, with cryo-ET providing direct 3D validation of the morphological defects observed in 2D images.

• Page 5, lines 13-14 ("revealed a sixfold symmetric structure") and Extended Data Figure 3: Was a parallel reconstruction produced from the same dataset without symmetry imposed?
Our response: We thank the reviewer for noting this issue. We now state that "The reconstruction of the LEN-bound lattice clearly resolved the central CA hexamer (Fig. 1f), and neighbouring hexamers were also discernible (Fig. 1e)" (Results section: lines 102–104).
In addition, we performed C1 refinement using a larger box size that included neighbouring hexamers, followed by heterogeneous classification (Fig. 3 and Supplementary Fig. 1-4). These analyses still resolved recognizable CA lattice features, allowing us to assess the arrangement of neighbouring hexamers without imposing sixfold symmetry.

• Page 2, line 12: The biochemical data support loss of reverse transcriptase activity, not strictly dissociation of the protein, although this is the most likely cause. The abstract need not be edited, but this distinction should be pointed out in the main text discussion.

Our response: We thank the reviewer for this important clarification. We agree that the original biochemical data primarily demonstrated a loss of RT activity, rather than directly showing the dissociation or loss of the RT protein.
In the revised manuscript, we performed a western blot analysis of the LEN− and LEN+ peak sucrose gradient fractions normalized to the p24 levels and confirmed that RT protein was markedly reduced in the LEN+ peak fraction (Fig. 5e). Thus, the reduced RT activity is supported by a corresponding reduction in the level of the core-associated RT protein.

• Consider including an annotated schematic of what primers for HIV DNA quantitation are targeting as in https://doi.org/10.1126/science.abc8420 Fig. 1 and refer to the extended data figure at the first mention of these targets.
Our response: We thank the reviewer for this helpful suggestion. We have added an annotated schematic of the reverse transcription process showing the positions of the primers used to quantify early and late reverse transcription products. We now refer to this schematic at the first mention of these qPCR targets in the revised manuscript and have included it as a new Supplementary Fig. 9.

• Please provide methodological detail for flow cytometry experiments: laser lines and filters
Our response: We have added the methodological details for the flow cytometry experiments, including the laser line and filter used for GFP detection, to the revised Methods section (Methods section: lines 429–432).

• Page 5, line 1: Citation 6 discusses GS-CA1, not GS-6207
Our response: We thank the reviewer for noting this issue. We have removed citation 6 from this sentence.

• Page 2, sentence 2 (lines 3-7) is a little wordy and difficult to understand on first reading
Our response: We have revised the sentence by splitting it into two sentences and simplifying the wording to improve readability (lines 23–27).

• Page 4, end of paragraph 1: capitalize Gag
Our response: We have corrected “gag” to “Gag” in the revised manuscript.

• Page 4, para. 2 (“alters the curvature parameters required for proper cone closure”): clarify that the question is whether curvature specifically is responsible. Citation 8 shows LEN stabilization of the CA hexamer lattice despite broken capsids not forming cones.

Our response: We thank the reviewer for this helpful suggestion. We have revised the text to clarify this point. (Introduction section: lines 77–80)

• It would be helpful to the reader to include an indicator of where the LEN binding site is on the structure overview e.g. Fig. 2a

Our response: We have added an indicator of the LEN binding site to the structural overview in the new Fig. 4a.

• Include labels on the figures for each subpanel so that readers have an easier time understanding the data without needing to read the details in the legend

Our response: We thank the reviewer for this helpful suggestion. We have added labels to the subpanels of the figures to make the data easier to understand without relying solely on the figure legends.

• Page 9, line 13 ("early reverse transcription (ERT)") and subsequent uses of "ERT": This can be confusing since "ERT" is often used to refer to endogenous reverse transcription. Consider using a different abbreviation.

Our response: We have replaced "early reverse transcription (ERT)" with "early reverse transcription (early RT)" throughout the revised manuscript to avoid confusion with endogenous reverse transcription.

Reviewer #3 (Remarks to the Author):

The manuscript by Tanaka et al. provides important structural insights into the late-stage effects of lenacapavir on the HIV-1 capsid architecture. The authors utilized complementary cryo-EM, biochemistry and virology assays. The study is generally well structured and follows a logical progression. Structural and functional data are well integrated. However, several key conclusions regarding capsid disruption and the mechanistic role of the lattice flattening require further clarification and additional experimental data. Addressing the points below would significantly strengthen the manuscript.

Our response: We thank the reviewer for the careful evaluation of our manuscript and for the positive comments on the structural and functional aspects of our study. We also appreciate the constructive suggestions, and we have revised the manuscript and added

additional experimental data where appropriate, as described in our point-by-point responses below.

Major comments

1. Quantification of aberrant capsid structures are needed. While the authors show that LEN induces irregular capsid morphologies, the observed morphological defects (e.g., percentages of intact vs fractured, enlarged, aberrant, or tubular structures in LEN untreated vs treated VLPs) are not quantitated. Furthermore, it would be important to assess how these phenotypes vary with varying LEN concentrations. The authors claim that partial LEN occupancy is sufficient to compromise capsid integrity which need to be supported by quantitation results.

Our response: We thank the reviewer for this important suggestion. We addressed this point by performing an additional cryo-ET analysis using NL4-3E– virions produced in the presence of 0, 2, 5, 10, and 50 nM LEN. We classified the particles into mature, immature, and atypical capsid morphologies and quantified the distribution of each class (Fig. 6d–j). In addition, we determined whether LEN compromises capsid integrity even in apparently mature particles by analysing only virions containing mature cone-shaped capsids and quantified visible gaps or fractures (Fig. 6l, m).

These analyses showed capsid morphological defects, including gaps, even in cone-shaped capsids, with detectable effects observed in the presence of 2–5 nM LEN. These results support the conclusion that capsid defects occur in the presence of low nanomolar concentrations of LEN and that complete LEN occupancy is not required to perturb proper capsid formation.

We have added these quantitative cryo-ET data to the revised manuscript (Fig. 6d–m) and revised the text accordingly (Results section: lines 223–253).

2. Cryo-ET experiments and inter-hexamer tilt angles require additional work. The authors use their own cryo-ET results with the VLPs prepared in the presence of LEN and compare these findings with the published structures of native capsid from other groups. The preparations of native cores and resolutions of the cryo-EM maps in published studies varies. Therefore, I strongly suggest the authors obtain their own cryo-ET data with untreated VLPs to side-by-side compare their own preparations of VLPs in the absence and presence of LEN. Furthermore, quantitative analysis (mean values with deviations) of the tilt angles between VLPs in the absence and presence of LEN are needed (for example see the recent preprint PMID: 41542592). Moreover, the authors need to detail the

methodology for calculating inter-hexamer tilt angles. It should be explained how hexamer orientations were defined, how angles were computed, and which programs were used.

Our response: We thank the reviewer for this important suggestion. We addressed this concern by determining our own LEN-unbound CA lattice structure from VLPs prepared under the same experimental conditions (Supplementary Figs. 2 and 4, Results section: lines 100–105) and directly compared it with the LEN-bound CA lattice. This side-by-side comparison confirmed that LEN reduces the interhexamer tilt compared with the untreated control (Fig. 2a).

In addition, we performed further cryo-EM analyses using a larger box size that included adjacent hexamers surrounding the central hexamer. The CA lattice was reconstructed in C1 without imposing symmetry (Fig. 3a–f and Supplementary Figs. 1-4), followed by a heterogeneous classification of the particles (Supplementary Fig. 2 and 4). This analysis resolved multiple classes of capsid lattice conformations. Quantification of the interhexamer tilt angles in each class revealed that LEN-containing lattices consistently exhibited reduced tilt angles along the curvature axis compared with LEN-unbound lattices (Fig. 3h). In contrast, little difference was observed along the flat axis (Fig. 3g). These results indicate that LEN-induced lattice flattening is not restricted to a single averaged reconstruction but represents a general structural feature of LEN-containing capsid lattices (Results section: lines 126–143).

We have added the results of the quantitative analysis of the interhexamer tilt angles, including the mean values and deviations, to the revised manuscript (Fig 3g, h). We have also revised the Methods section to describe how hexamer orientations were defined, how angles were computed, and which programs were used (Methods section: lines 530–536).

3. Information regarding pentamer-hexamer interfaces is lacking. The structural analysis focuses primarily on inter-hexamer interactions and lattice flattening. However, capsid curvature and closure critically depend on pentamers. Given that LEN binds only hexamers, it would be important to address how LEN affects pentamer–hexamer interfaces. Is the lattice flattening alone sufficient to induce rupture or are high-curvature regions (pentamer-rich) preferentially destabilized?

Our response: We thank the reviewer for this important comment. We agree that pentamer–hexamer interfaces are critical for capsid curvature and closure. However, in the current dataset, we were not able to reconstruct a pentamer-containing lattice at sufficient resolution to directly analyse pentamer–hexamer interfaces. One possible explanation is

that LEN may impair CA pentamer formation, as suggested in a recent study (Huang, *et al.*, PLOS Pathog., 2025; PMID: 39869652), thereby limiting the number of suitable pentamer-containing lattice particles available for reconstruction.

Although we could not directly resolve pentamer–hexamer interfaces, we sought to assess whether high-curvature regions of capsids are preferentially affected by LEN. To this end, we added a cryo-ET analysis of apparently cone-shaped capsids produced in the presence of low LEN concentrations (Fig. 6d–m, related to Comment 2 from Reviewer 3). The results of this analysis showed that visible gaps tended to occur in high-curvature regions of the cone-shaped capsid under sub-nano LEN conditions (Fig. 6l). These observations suggest that high-curvature regions may be particularly susceptible to LEN-induced lattice perturbation, although we cannot directly resolve the pentamer–hexamer interface in the present analysis.

We have added this information to the revised Results and Discussion (lines 223–253 and lines 351–367).

4. The VLPs produced in the presence of LEN appear structurally heterogeneous. It is therefore unclear whether the reconstructed lattice represents a single dominant conformation or an average over multiple structural states. The authors should address the degree of particle heterogeneity and whether multiple classes of capsid architectures exist. A larger box size of the capsid lattice would give a larger cryo-EM map with better resolved adjacent hexamers that surround the central hexamer. Subsequent heterogeneous refinements of the consensus map could delineate the different classes (different curvatures, etc).

Our response: We thank the reviewer for this important suggestion. This point is related to Comment 2, and the additional cryo-EM analyses described above also address this concern. Briefly, we performed additional analyses using a larger box size that included adjacent hexamers surrounding the central hexamer. The CA lattice was reconstructed in C1 without imposing symmetry during refinement, followed by a heterogeneous classification of the particles (Fig. 3, Supplementary Figs. 1–4).

This analysis resolved multiple classes of capsid lattice conformations with different degrees of curvature (Fig. 3g, h; Supplementary Figs. 2 and 4). Importantly, compared with LEN-unbound lattices, LEN-bound lattices consistently showed reduced interhexamer tilt along the curvature axis across these classes, whereas little difference was observed along the flat axis (Fig. 3g, h). These results indicate that the reconstructed LEN-bound lattice is not simply an average of unrelated structural states but that lattice flattening is a common structural feature among heterogeneous LEN-containing capsid lattices.

We have incorporated these analyses into the revised manuscript and added the corresponding data to Fig. 3 and Supplementary Figs. 1–4. (Results section: lines 126–143).

Minor comments

5. Cryo-EM processing workflow need to be described better. The cryo-EM data processing is unclear, particularly regarding the sequence of heterogeneous refinement and 2D classification steps. Please clarify the order of processing steps, and the rationale for the chosen workflow. The author used C6 symmetry in a heterogeneous refinement after the initial 2D classification. Also, no ab-initio reconstruction step with C1? Please provide the reasons for doing so.

Our response: We thank the reviewer for this helpful comment. We have revised the Methods section (Methods section: lines 495–509) and Supplementary Figs. 2, 4 and 6 to describe the cryo-EM processing workflow more clearly.

C6 symmetry was applied during heterogeneous refinement because the particles used for high-resolution reconstruction corresponded to local CA hexameric lattice units. After the initial 2D classification, the selected particles showed clear sixfold hexameric features, and our primary aim in this reconstruction was to resolve the local LEN-bound CA hexameric lattice. We therefore used a previously determined mature CA hexamer structure as the initial reference. Similar C6-symmetry-based strategies have been used in previous cryo-EM studies of HIV-1 CA hexameric lattices (Representative studies: Stacey *et al.*, Proc Natl Acad Sci U S A., 2023; PMCID:PMC10120081, Zhu *et al.*, Nat Commun., 2025; PMCID:PMC12402280), supporting the validity of this approach for improving particle alignment and resolving local hexameric CA lattice features. For this reason, an initial ab initio C1 reconstruction was not used for the main reconstruction.

We agree that C6 symmetry could mask heterogeneity in the surrounding lattice geometry. We addressed this concern by performing an additional C1 analysis using a larger box size that included neighbouring hexamers, followed by heterogeneous classification and C1 refinement. This analysis confirmed that the LEN-bound lattice adopts a flattened interhexamer geometry and allowed us to assess the surrounding lattice arrangement without imposing C6 symmetry (Fig. 3).

6. The structural analyses are performed at 50 nM LEN, which exceeds reported pharmacologically relevant concentrations of the antiretroviral (~5 nM). While the authors justify this choice to ensure high occupancy, it raises a possibility that the observed

structural effects reflect near-saturating conditions. Clarify how these findings relate to physiological conditions and, if possible, include supporting structural data for lower LEN concentrations.

Our response: We performed an additional concentration-dependent cryo-ET analysis using NL4-3E– VLPs produced in the presence of 0, 2, 5, 10, and 50 nM LEN to address the relevance of lower LEN concentrations (Fig. 6d–m). The results of this analysis revealed capsid morphological defects, including visible gaps even in apparently cone-shaped capsids, with detectable effects already observed in the presence of 2–5 nM LEN (Fig. 6d–m). In addition, RT leakage was observed in the presence of 2 nM LEN (Fig. 5f). Since the pharmacologically relevant concentration of LEN is approximately 5 nM, these data indicate that LEN can perturb the capsid morphology and compromise capsid integrity within a low-nanomolar concentration range. We have added this point to the revised Results and Discussion sections (lines 223–253 and lines 332–334).

7. The results showing the RT activity loss and impaired ERT in the target cells are exciting. I suggest supplementing these results by examining RT levels by immunoblotting analysis of sucrose gradient fractions (similar to CA and IN immunoblots shown in Fig 3,a,b,d,e). If RT antibodies are not sensitive enough, the total protein from the peak fractions could be precipitated to assess RT levels by western blot.
Our response: We thank the reviewer for this helpful suggestion. We have now examined RT protein levels by western blotting using the LEN– and LEN+ peak sucrose-gradient fractions (Fig. 5e). The input samples were normalized to equivalent amounts of p24 before loading to enable a direct comparison. Under these p24-normalized conditions, the level of RT was markedly reduced in the LEN+ peak fraction, whereas IN remained detectable (Fig. 5e).
These data support the SG-PERT results and indicate that the reduced RT activity reflects a reduction in core-associated RT protein. We have added this point to the revised Results section (lines 186–192).

8. Unit/formatting issue. The sentence referring to a “5 nM2” concentration appears to contain a formatting error and should be corrected.
Our response: We thank the reviewer for noting this issue. The “2” above 5 nM was intended to indicate reference 2 (now updated to reference 28), not a unit. We have revised the text to clarify this notation.

9. I suggest that authors discuss preprint PMID: 41542592. The findings in the preprint and the current manuscript are similar in terms of LEN fracturing the capsid, which in turn results in the RT loss. However, the important distinctions between these two studies are that the current manuscript shows that LEN can induce both the lattice enlargement and fracturing during HIV-1 particle assembly, whereas the preprint demonstrates that LEN can additionally fracture the properly matured cores.

Our response: We thank the reviewer for this helpful suggestion. We have added a discussion of the recent preprint (Santos *et al*., BioRxiv, 2026; PMID: 41542592) in the revised manuscript. (Discussion section; lines 368–378)

We agree that the preprint and our study are complementary. Both studies support the idea that LEN can compromise capsid integrity by reducing the local lattice curvature. However, the experimental contexts are distinct: the preprint shows that LEN can fracture properly matured cores, whereas our study focuses on virions produced in the presence of LEN and shows that LEN induces capsid defects, including an abnormal lattice organization and incomplete capsid formation, during HIV-1 particle assembly and maturation.

We have revised the Discussion to emphasize this distinction and to place our findings in the context of LEN-induced capsid defects both during virion maturation and after mature core formation. (Discussion section; lines 368–378)

Reviewer #4 (Remarks to the Author):

I co-reviewed this manuscript with one of the reviewers who provided the listed reports. This is part of the Nature Communications initiative to facilitate training in peer review and to provide appropriate recognition for Early Career Researchers who co-review manuscripts.

Our response: We thank the reviewer for the careful evaluation of our manuscript

Reviewer #5 (Remarks to the Author):

I co-reviewed this manuscript with one of the reviewers who provided the listed reports. This is part of the Nature Communications initiative to facilitate training in peer review and to provide appropriate recognition for Early Career Researchers who co-review manuscripts.

Our response: We thank the reviewer for the careful evaluation of our manuscript

Point-by-point responses

Reviewer #1 (Remarks to the Author):

The authors have addressed all of the concerns and comments that we raised in our review of the original manuscript. As a result, the manuscript is significantly improved and makes a strong contribution to our understanding of how lenacapavir influences HIV-1 capsid structure and infectivity.

Response)

We thank the reviewer for their positive assessment of our revised manuscript.

In their response to the reviewer comments, the authors have added results of two additional sucrose gradient fractionation experiments, and measured RT activity in the presence of increasing concentrations of sucrose to show that sucrose levels did not influence RT activity. These data should be included as supplemental data in the revised manuscript, so that other readers who may have the same questions will have the data available.

Response)

We thank the reviewer for this suggestion. We have now included the results of the two additional sucrose gradient fractionation experiments in Supplementary Fig. 8 and the analysis of RT activity in the presence of increasing concentrations of sucrose in Supplementary Fig. 9 of the revised manuscript.

In addition, the authors purified virions through a second sucrose cushion to remove residual free lenacapavir to show that the infectivity of the purified particles was not affected by carryover of free lenacapavir. If possible, the authors should add these data as a supplemental figure.

Response)

We thank the reviewer for this comment. The data showing the infectivity of virions purified through a second sucrose cushion to remove residual free lenacapavir are already included in Supplementary Fig. 10 of the revised manuscript.
