# Supplementary material for: Structural basis of lenacapavir-induced HIV-1 capsid defects during virion maturation
Source: Nat Commun. 2026 Sep 22;17:9830. doi: 10.1038/s41467-026-77803-7 (PMC13598136; doi:10.1038/s41467-026-77803-7)
Supplement: Supplementary file 3 [file 41467_2026_77803_MOESM1_ESM.pdf]

Supplementary Information

Structural basis of lenacapavir-induced HIV-1 capsid defects during virion maturation

Hiroki Tanaka[1], Reina Morita[1], Tomomasa Oka[1], Minoru Fukushima[1], Shunsuke Kita[2], Mina Sasaki[2], Katsumi Maenaka[2,3,4,5], and Shinichi Machida[1,6,*]

[1]Department of Structural Virology, National Institute of Global Health and Medicine, Japan Institute for Health Security, 1-21-1 Toyama, Shinjuku-ku, Tokyo, 162-8655, Japan.

[2]Laboratory of Biomolecular Science, Faculty of Pharmaceutical Sciences, Hokkaido University, Sapporo 060-0812, Japan.

[3]Center for Research and Education on Drug Discovery, Faculty of Pharmaceutical Sciences, Hokkaido University, Sapporo, 060-0812, Japan.

[4]Global Station for Biosurfaces and Drug Discovery, Hokkaido University, Sapporo, 060-0812, Japan.

[5]Hokkaido University Institute for Vaccine Research & Development, Sapporo, 060-0812, Japan.

[6]Graduate School of Medical Sciences, Kumamoto University, Kumamoto, Japan.

*Corresponding author. Email: machida.s@jihs.go.jp

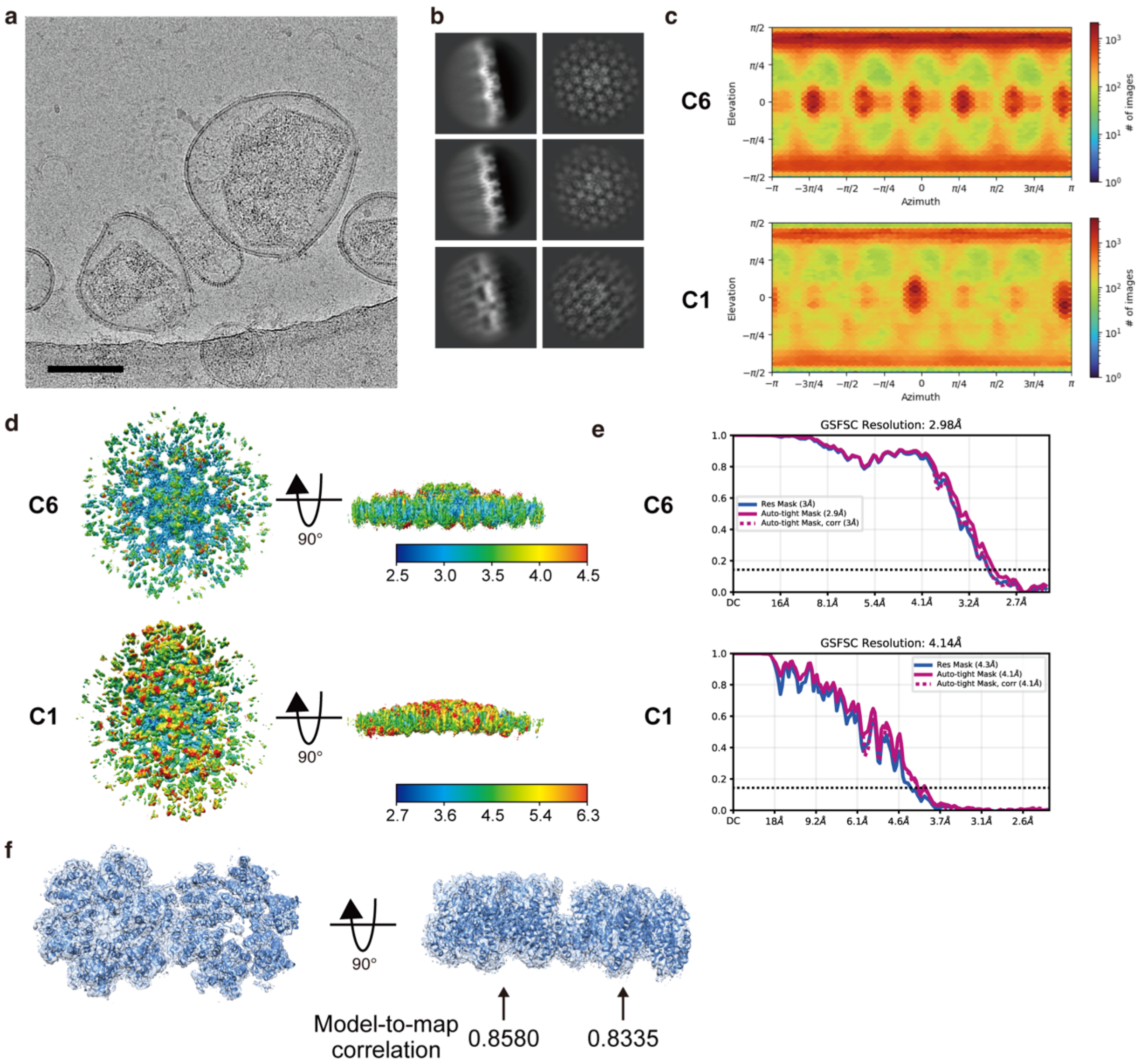

Supplementary Fig. 1. Cryo-EM analysis of the LEN-bound HIV-1 capsid lattice within VLPs treated with PFO.

a, Representative micrograph from the cryo-EM imaging dataset. Scale bar, 100 nm. **b**, Selected 2D class averages. **c**, Euler angle distribution plots of the particles of the LEN-bound CA lattice contributing to the final reconstruction. **d**, Local resolution map of the LEN-bound CA lattice. **e**, Fourier shell correlation (FSC) curve for the LEN-bound CA lattice. The dashed line indicates the FSC threshold of 0.143. **f**, Model-to-map fit of

adjacent CA hexamer models in the C6-symmetric LEN-bound CA lattice map. Model-to-map correlation values were calculated using “fit in map” tool in UCSF Chimera.

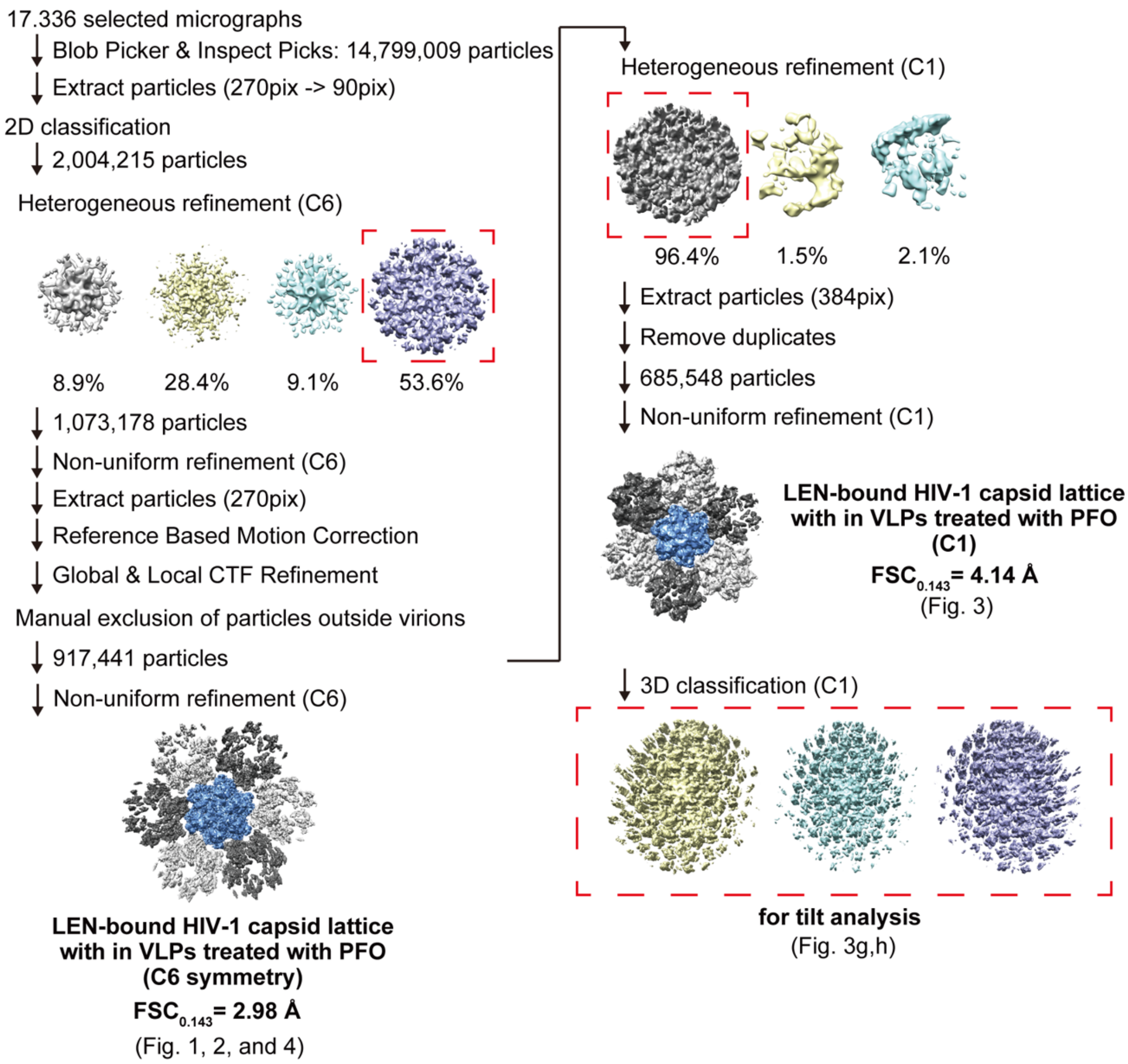

Supplementary Fig. 2. Data processing workflow for the LEN-bound HIV-1 capsid lattice within VLPs treated with PFO.

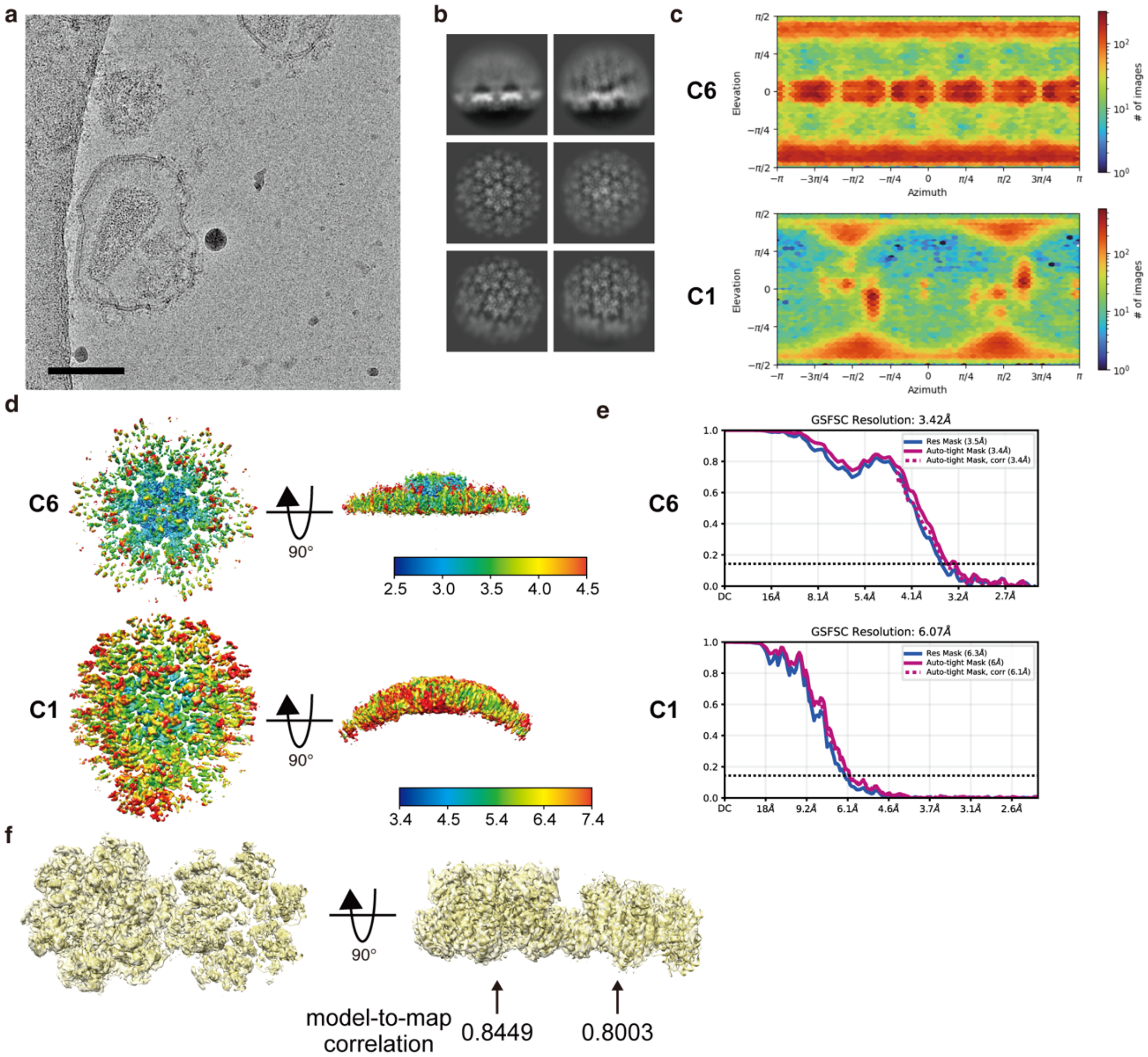

Supplementary Fig. 3. Cryo-EM analysis of the LEN-unbound HIV-1 capsid lattice within VLPs treated with PFO.

a, Representative micrograph from the cryo-EM imaging dataset. Scale bar, 100 nm. **b**, Selected 2D class averages. **c**, Euler angle distribution plots of the particles of the LEN-unbound CA lattice contributing to the final reconstruction. **d**, Local resolution map of the LEN-unbound CA lattice. **e**, Fourier shell correlation (FSC) curve for the LEN-unbound CA lattice. The dashed line indicates the FSC threshold of 0.143. **f,** Model-to-map fit of adjacent CA hexamer models in the C6-symmetric LEN-unbound CA lattice

map. Model-to-map correlation values were calculated using “fit in map” tool in UCSF Chimera.

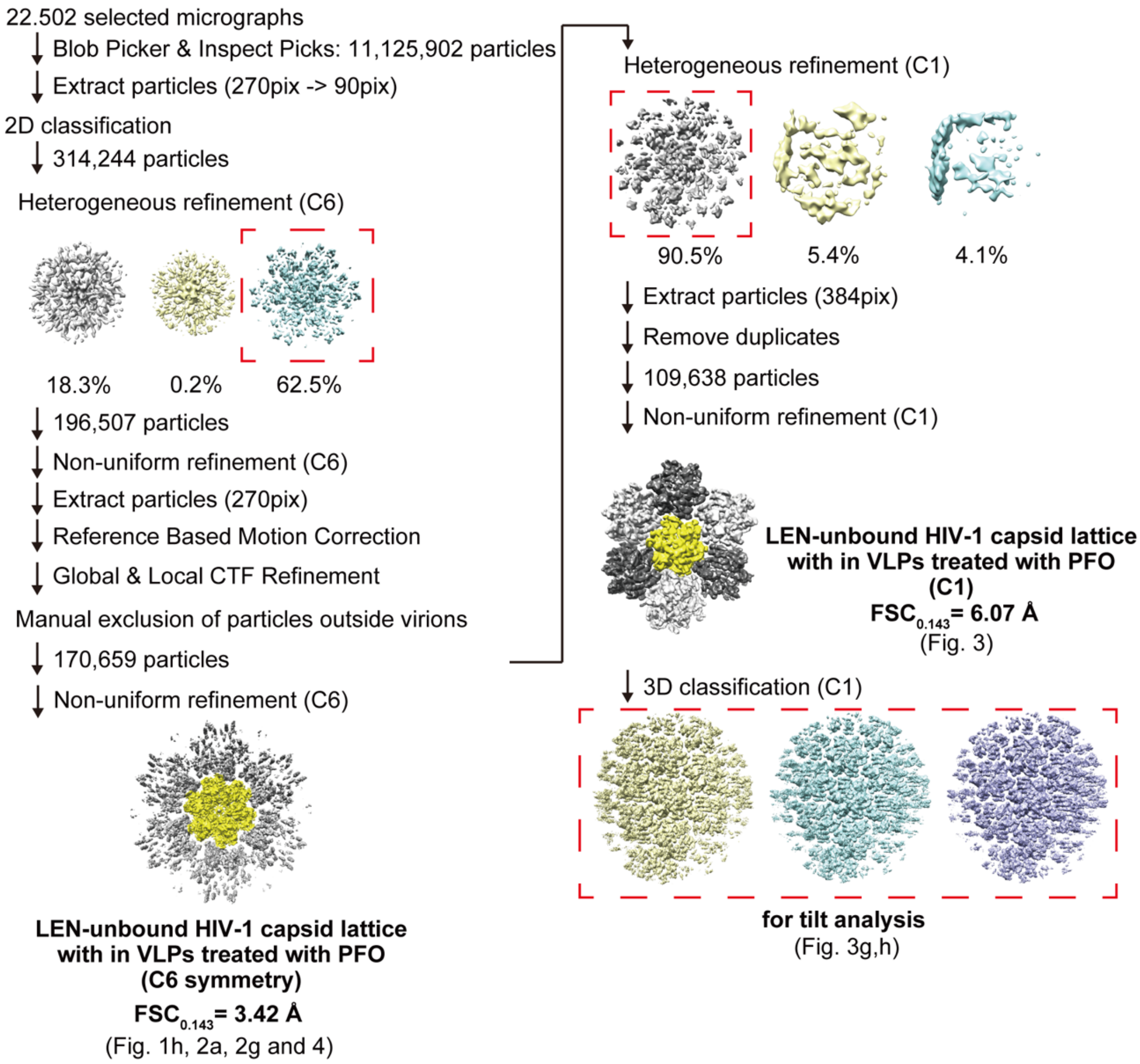

Supplementary Fig. 4. Data processing workflow for the LEN-unbound HIV-1 capsid lattice within VLPs treated with PFO.

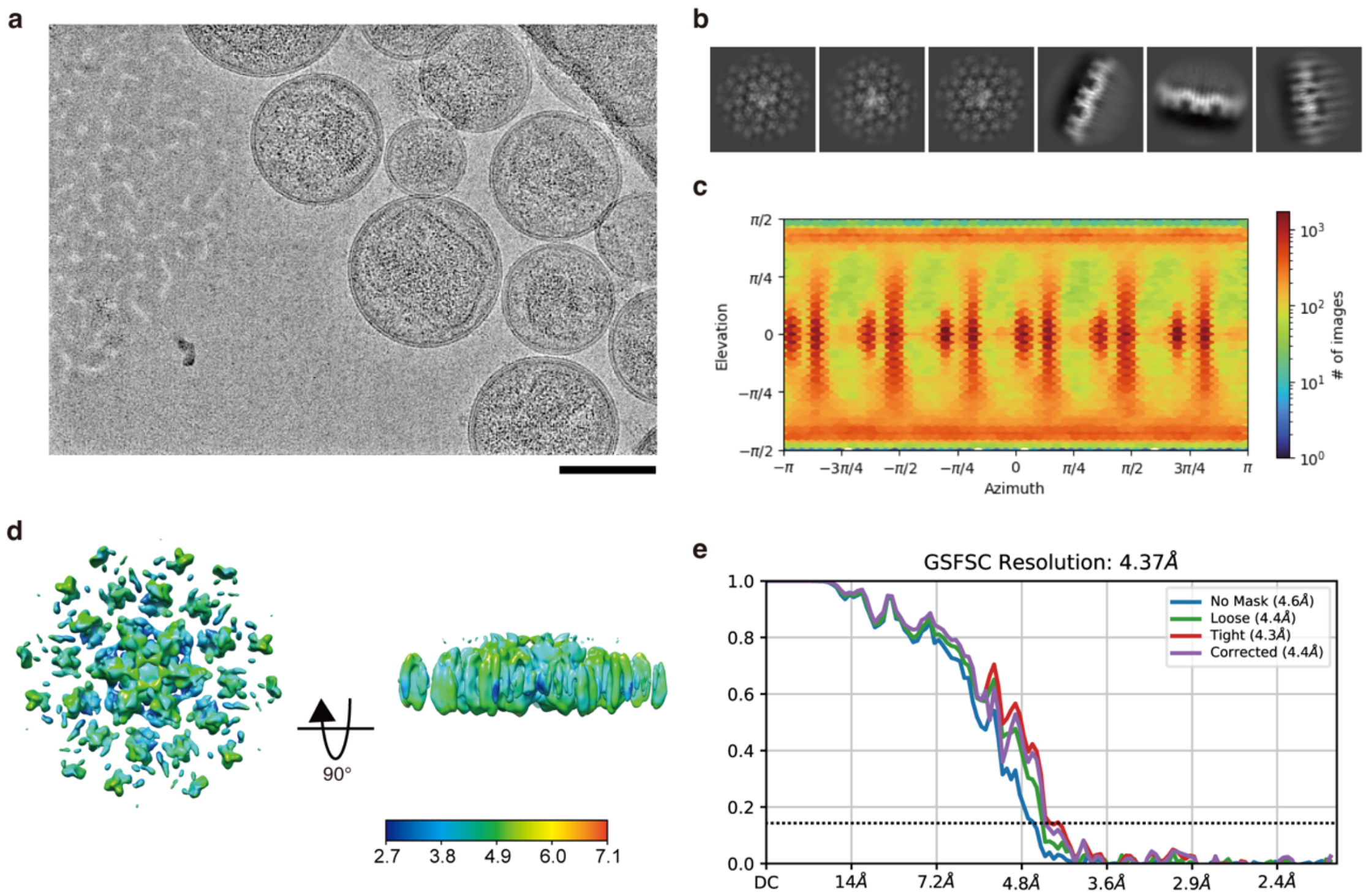

Supplementary Fig. 5. Cryo-EM analysis of the LEN-bound HIV-1 capsid lattice within intact VLPs.

a, Representative micrograph from the cryo-EM imaging dataset. Scale bar, 100 nm. **b**, Selected 2D class averages. **c**, Euler angle distribution plots of the particles of the LEN-bound CA lattice contributing to the final reconstruction. **d**, Local resolution map of the LEN-bound CA lattice. **e**, Fourier shell correlation (FSC) curve for the LEN-bound CA lattice. The dashed line indicates the FSC threshold of 0.143.

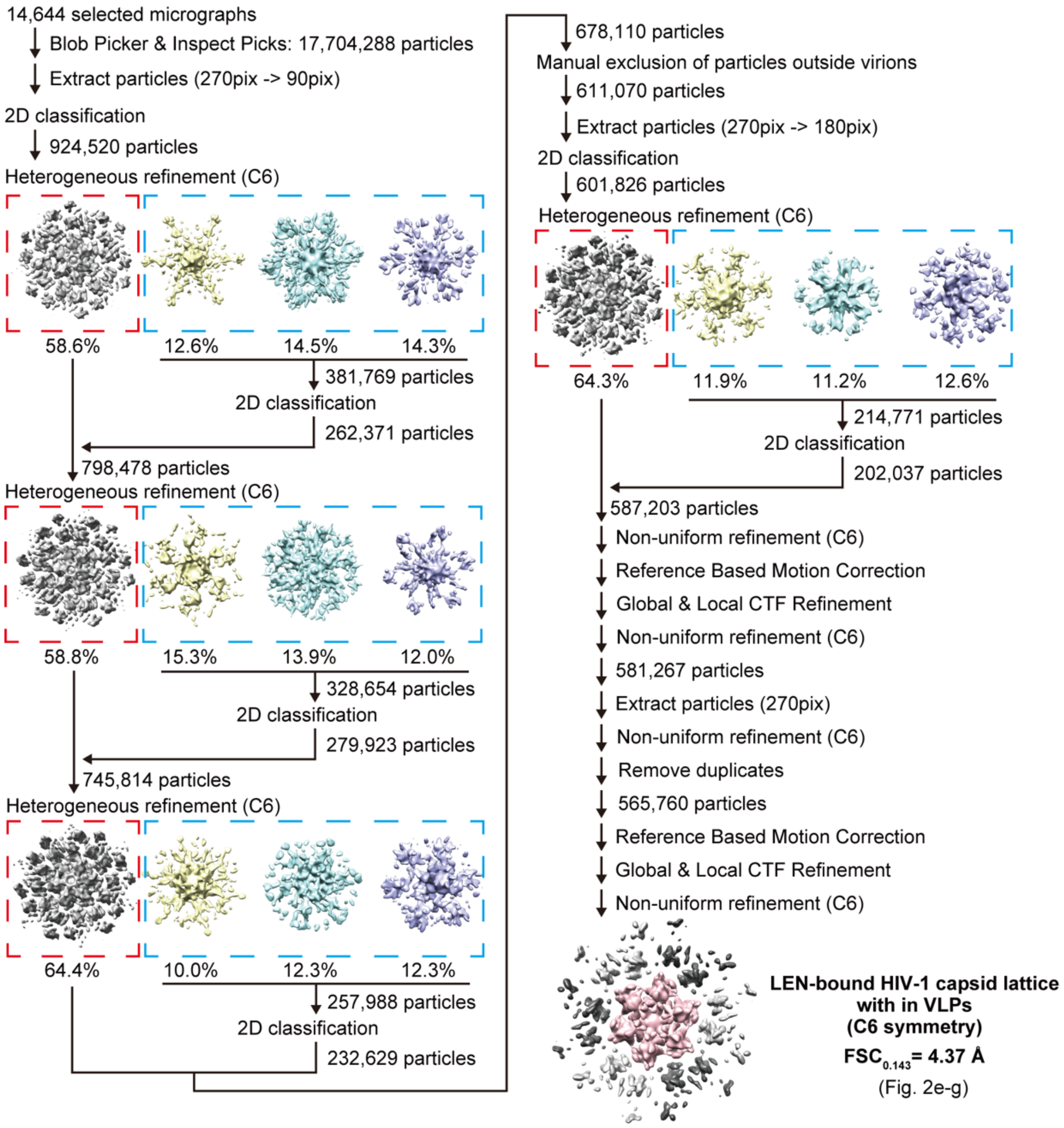

Supplementary Fig. 6. Data processing workflow for the LEN-bound HIV-1 capsid lattice within intact VLPs.

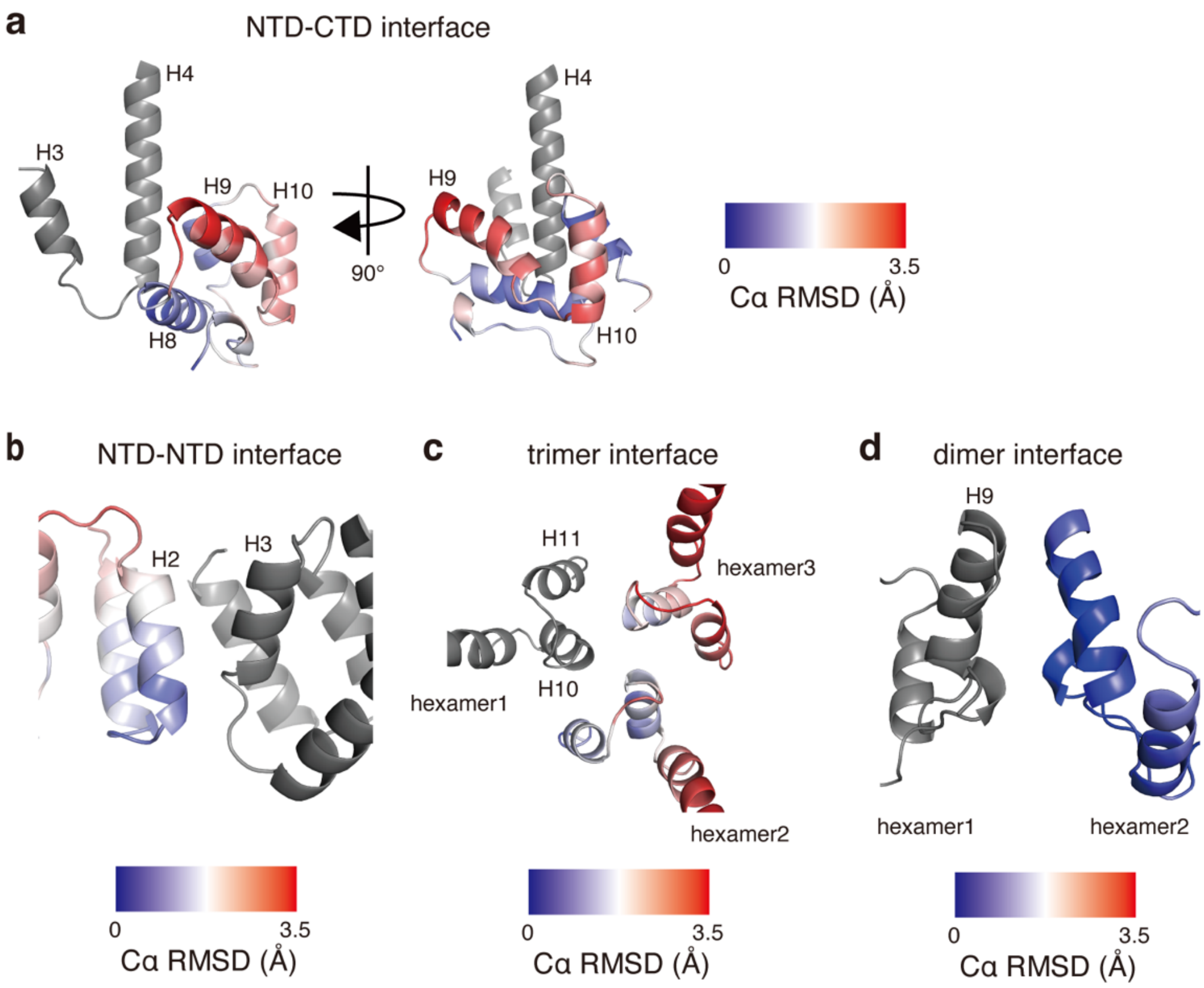

Supplementary Fig. 7. RMSD-based comparison of intra- and interhexamer interfaces within the LEN-unbound and LEN-bound capsid lattices.

a, Comparison of the NTD–CTD interface between CA molecules in the region surrounding the FG pocket based on the cryo-EM structures of the LEN-bound and LEN-unbound CA lattices. This panel corresponds to Fig. 4b. **b,** Comparison of the NTD–NTD interface between CA molecules mediated by helices 2 and 3 based on the cryo-EM structures of the LEN-bound and LEN-unbound CA lattices. This panel corresponds to Fig. 4c. **c,** Comparison of the CTD-mediated trimer interface between CA molecules mediated by primary helix 10 based on the cryo-EM structures of the LEN-bound and LEN-unbound CA lattices. This panel corresponds to Fig. 4d. **d,** Comparison of the CTD–CTD dimer interface between CA molecules mediated by helix 9 based on the cryo-EM structures of the LEN-bound and LEN-unbound CA lattices. This panel corresponds to Fig. 4e. Models are coloured according to the RMSDs between the LEN-bound and LEN-unbound CA structures, from blue (0 Å RMSD) to red (3.5 Å RMSD).

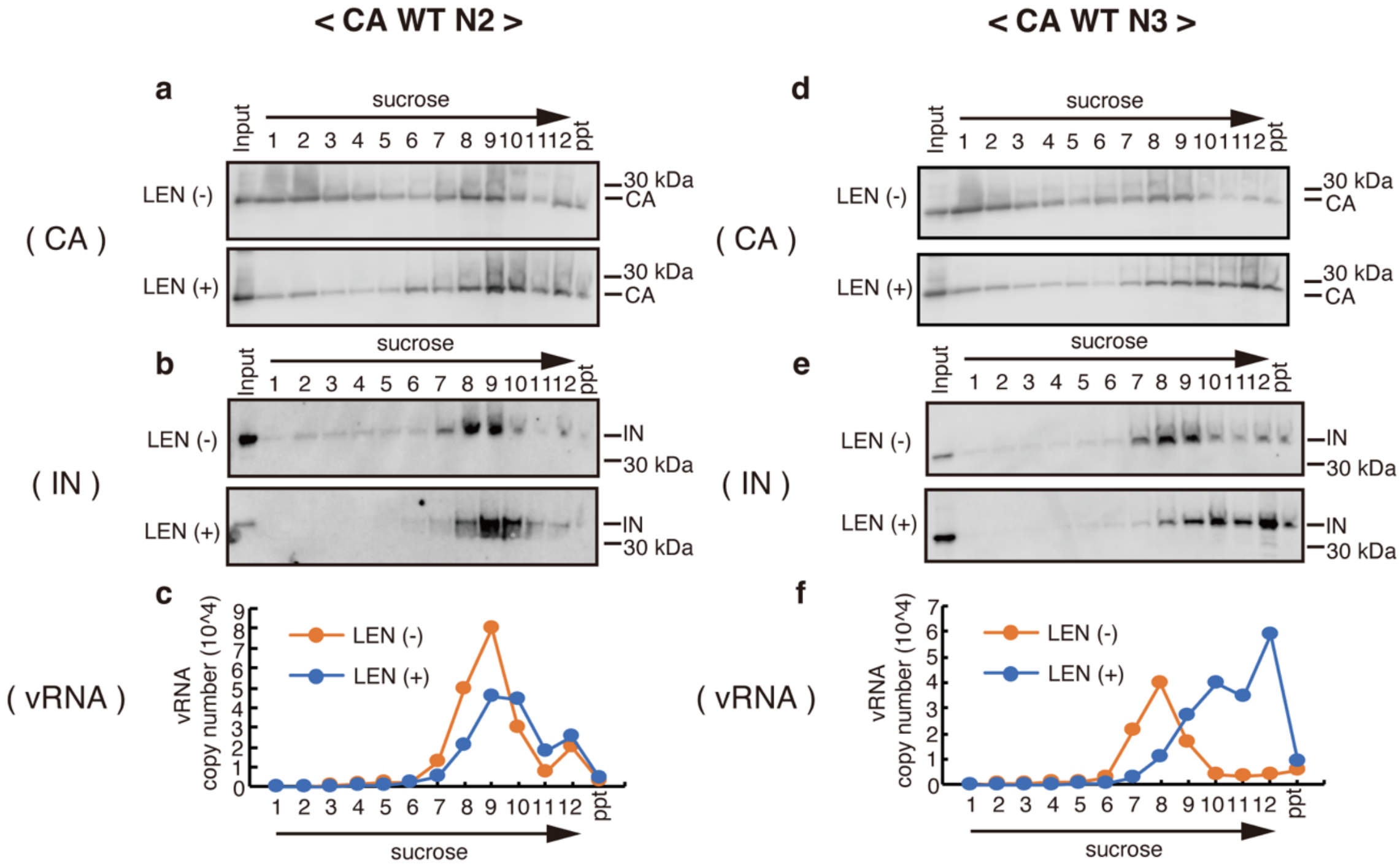

Supplementary Fig. 8. Reproducibility of sucrose gradient fractionation of WT HIV-GFP virions produced in the presence or absence of LEN.

CA (a, d) and IN (b, e) levels were detected by western blotting. The amount of viral RNA in each fraction was quantified by RT–qPCR (c, f). The first experiment is shown as representative data in Fig. 5a–c, and the results of the two additional independent experiments are provided.

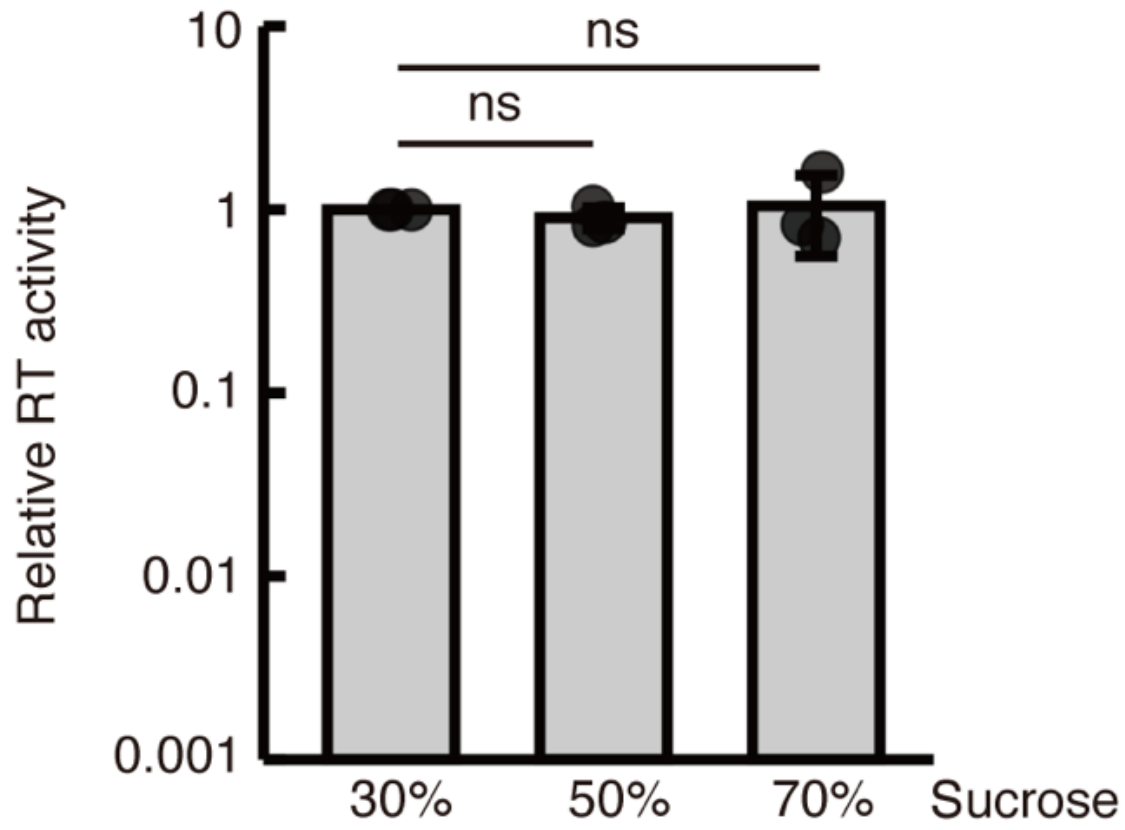

Supplementary Fig. 9. Effect of sucrose concentration on reverse transcriptase activity.

Reverse transcriptase (RT) activity was measured in the presence of 30%, 50%, and 70% sucrose. RT activity was normalized to that of the 30% sucrose sample. The data are shown as the means ± s.d. (n = 3 independent experiments). Statistical significance was assessed using a paired two-tailed Student's t-test. (30% vs 50%, P = 0.296; 30% vs 70%, P = 0.883)

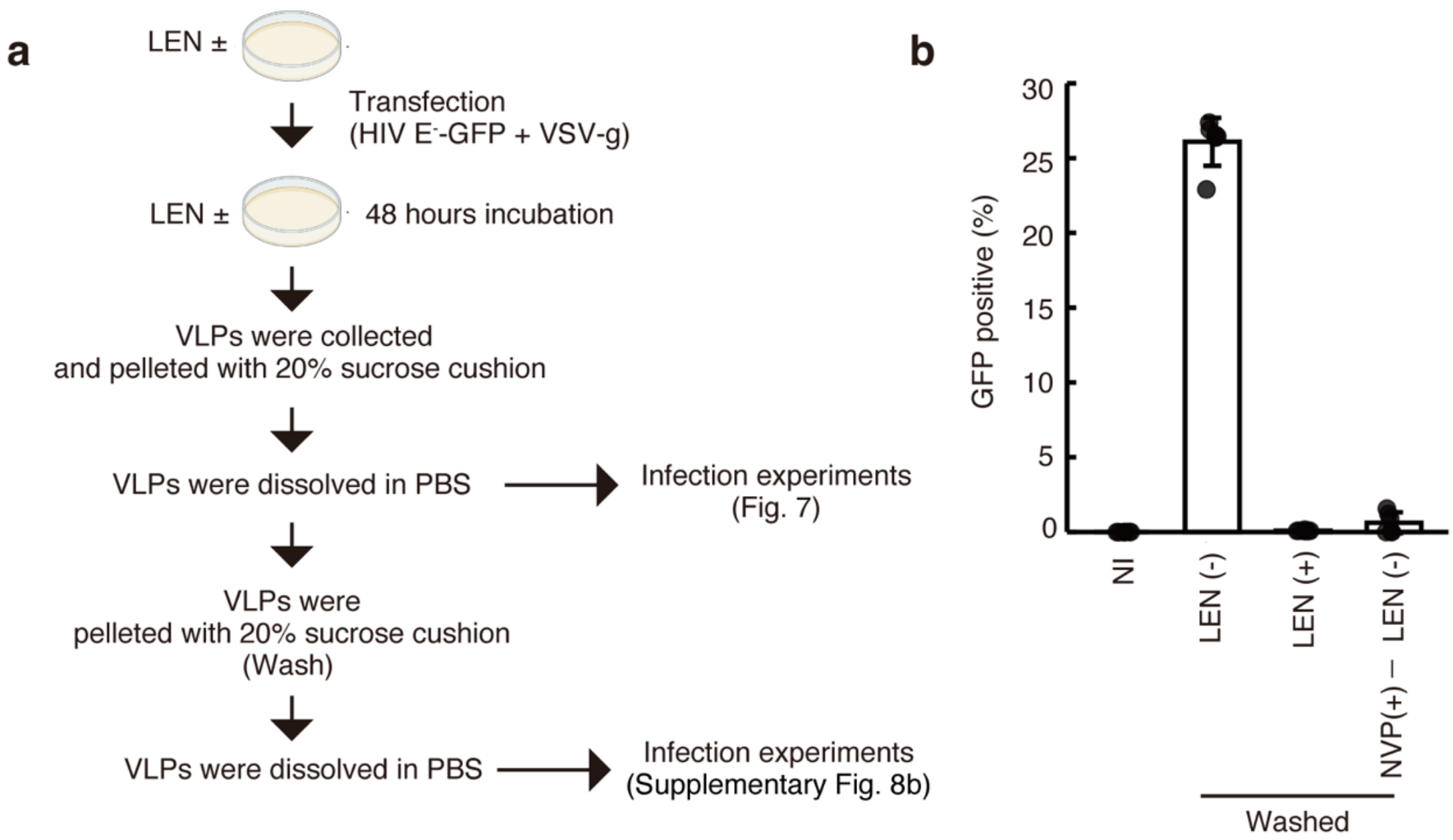

Supplementary Fig. 10. HIV-1 produced in the presence of LEN exhibits reduced infectivity.

a. Schematic of the experimental design. HIV-GFP virions were produced in the presence of DMSO or 50 nM LEN and harvested at 48 h post-transfection. Virions were pelleted through a 20% sucrose cushion to remove the residual free LEN before being used for the infectivity assay shown in Fig. 7. Virions were washed with PBS and pelleted again through a 20% sucrose cushion before being used for the infectivity assay shown in (b) to further minimize LEN carryover. **b.** Infectivity in Jurkat cells infected with HIV-GFP virions produced in the presence or absence of 50 nM LEN after an additional wash through a sucrose cushion. The data are presented as the means ± s.d.; n = 6 independently treated cell cultures.

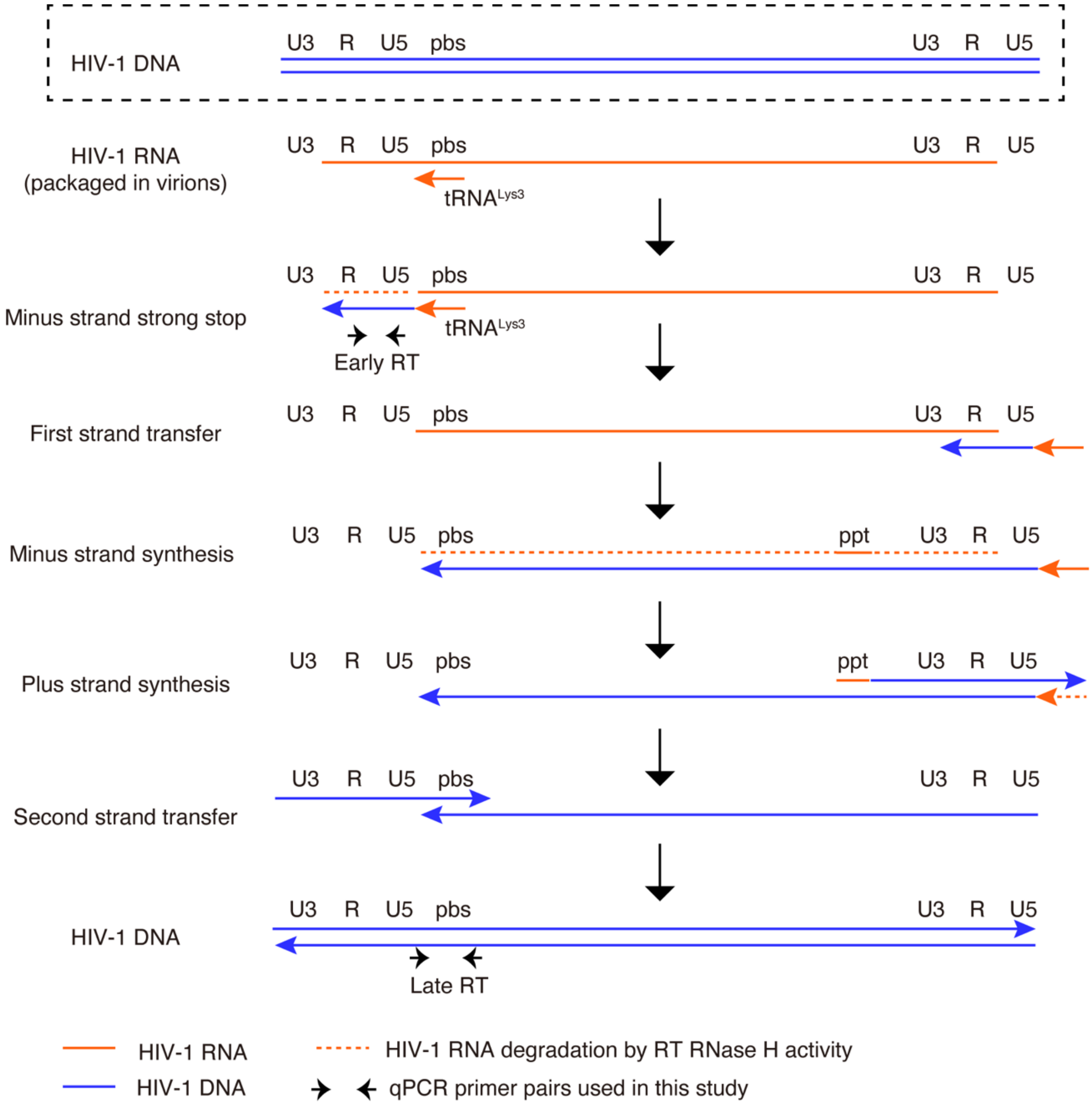

Supplementary Fig. 11. Schematic of HIV-1 reverse transcription and the positions of the primers used for qPCR in this study.

Early RT primers amplify minus-strand strong stop DNA (early reverse transcription products), whereas late RT primers amplify late reverse transcription products after plus-strand synthesis. Red and blue lines indicate HIV-1 RNA and DNA, respectively. Dashed orange lines indicate RNA degraded by the RNase H activity of reverse transcriptase. The U3, R, and U5 regions in the LTRs and the ppt (polypurine tract) are indicated. The schematic was adapted from Christensen *et al.*[45].

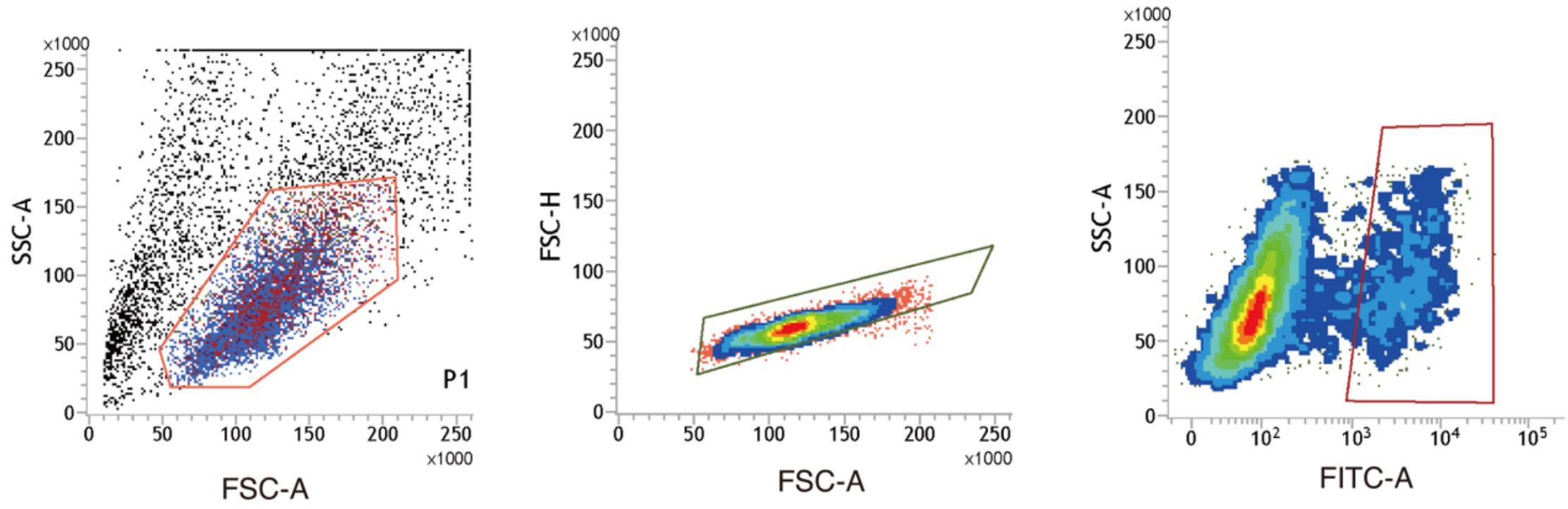

Supplementary Fig. 12. Flow cytometry gating strategy.

The first gate (FSC-A versus SSC-A) selects cells and excludes debris. The second gate (FSC-A versus FSC-H) selects singlets. The third gate (FITC-A versus SSC-A) identifies GFP-positive cells.

Supplementary Table 1. Median and mean areas of virus-like particles produced in the presence or absence of LEN.

	DMSO	50 nM LEN
Median (nm^2)	13525	15415
Mean (nm^2)	13607	20188

Supplementary Table 2. Cryo-EM data collection, refinement and validation statistics.

	LEN-bound CA lattice within VLPs treated with PFO (C6 sym.)	LEN-bound CA lattice within VLPs treated with PFO (C1)	LEN-unbound CA lattice within VLPs treated with PFO (C6 sym.)	LEN-unbound CA lattice within VLPs treated with PFO (C1)	LEN-bound CA lattice within VLPs (C6 sym.)
**Data collection**					
Microscope	Krios G4		Krios G4		Krios G4
Source	E-CFEG		E-CFEG		X-FEG
Detector	Falcon 4i		Falcon 4i		K3
Energy Filter	Selectris		Selectris		BioContinuum
Slit Width (eV)	10		10		20
Magnification	105,000		105,000		81,000
Voltage (kV)	300		300		300
Total dose (e–/Å^2)	53.2		48.4		50.8
Defocus range (μm)	-0.8 to -2.4		-0.8 to -2.4		-0.8 to -2.4
Pixel size (Å)	1.2		1.2		1.06
**Data processing**					
Final particle images (no.)	917,441	685,548	170,659	109,638	565,760
Map resolution (Å)	2.98	4.14	3.42	6.07	4.37
FSC threshold	(FSC=0.143)	(FSC=0.143)	(FSC=0.143)	(FSC=0.143)	(FSC=0.143)
Symmetry imposed	C6	C1	C6	C1	C6
EMDB ID	81945	81946	81947	81948	67116
**Refinement**					
Initial model used (PDB code)	4XFX		4XFX		4XFX
Model composition					
Non-hydrogen atoms	9744		9744		4992
Protein residues	1248		1248		1248
Ligands	0		0		0
R.m.s. deviations					
Bond lengths (Å)	0.005		0.004		0.005
Bond angles (°)	0.945		0.925		1.143
Validation					
MolProbity score	1.66		1.75		0.89
Clashscore	11.37		14.09		0.95
Poor rotamers (%)	0		0		0
Ramachandran plot					
Favored (%)	97.55		97.55		97.47
Allowed (%)	2.45		2.45		2.53
Disallowed (%)	0		0		0
PDB ID	43KN		43KO		9XQH

Supplementary Table 3. Median and mean areas of HIV-1 virions produced in the presence or absence of LEN.

	DMSO	2 nM LEN	5 nM LEN	10 nM LEN	50 nM LEN
Median (nm^2)	12035	11844	12081	12815	13461
Mean (nm^2)	12514	12330	13046	14092	17014

Supplementary Table 4. Primers used for the quantification of HIV DNA.

Name	Sequence (5'–3')	Target	References
Early RT_F	GGCTAACTAGGGAACCCACTGC	Early RT product	
Early RT_R	CAACAGACGGGCACACACTACT	Early RT product	
Late RT_F	TGTGTGCCCGTCTGTTGTGT	Late RT product	([45])
Late RT_R	CTTCAGCAAGCCGAGTCCTG	Late RT product	([45])
L-M667	ATGCCACGTAAGCGAAACTCT GGCTAACTAGGGAACCCACTG	Integrated HIV (Alu-PCR (1st round of PCR))	([44,46,47])
Alu 1	TCCCAGCTACTGGGGAGGCTGAGG	Integrated HIV (Alu-PCR (1st round of PCR))	([44,46,47])
Alu 2	GCCTCCCAAAGTGCTGGGATTACAG	Integrated HIV (Alu-PCR (1st round of PCR))	([44,46,47])
Lambda T	ATGCCACGTAAGCGAAACT	Integrated HIV (Alu-PCR (2nd round of PCR))	([44,46,47])
AA55M	GCTAGAGATTTTCCACACTGACTAA	Integrated HIV (Alu-PCR (2nd round of PCR))	([44,46,47])
β-globin-F	CCCTTGGACCCAGAGGTTCT	β-globin	([44,46,47])
β-globin-R	CGAGCACTTTCTTGCCATGA	β-globin	([44,46,47])
