# Supplementary material for: Structural basis of lenacapavir-induced HIV-1 capsid defects during virion maturation
Source: Nat Commun. 2026 Sep 22;17:9830. doi: 10.1038/s41467-026-77803-7 (PMC13598136; doi:10.1038/s41467-026-77803-7)
Supplement: Supplementary file 4 [file 41467_2026_77803_MOESM2_ESM.pdf]

Description of Additional Supplementary Files

Supplementary Movie 1. | Cryo-ET of HIV-1 VLPs produced in the absence of LEN, corresponding to Fig. 6a.

Supplementary Movie 2. | Cryo-ET of HIV-1 VLPs produced in the presence of LEN, showing aberrant sheet-like lattices, corresponding to Fig. 6b.

Supplementary Movie 3. | Cryo-ET of HIV-1 VLPs produced in the presence of LEN, showing tubular or incompletely closed capsid structures, corresponding to Fig. 6c.

Supplementary Movie 4. | Cryo-ET of HIV-1 virions produced in the absence of LEN, corresponding to Fig. 6d.

Supplementary Movie 5. | Cryo-ET of HIV-1 virions produced in the presence of 2 nM LEN, corresponding to Fig. 6e.

Supplementary Movie 6. | Cryo-ET of HIV-1 virions produced in the presence of 5 nM LEN, corresponding to Fig. 6f.

Supplementary Movie 7. | Cryo-ET of HIV-1 virions produced in the presence of 10 nM LEN, corresponding to Fig. 6g.

Supplementary Movie 8. | Cryo-ET of HIV-1 virions produced in the presence of 50 nM LEN, corresponding to Fig. 6h.
